# Increased diversity of egg-associated bacteria on brown trout (*Salmo trutta*) at elevated temperatures

**DOI:** 10.1038/srep17084

**Published:** 2015-11-27

**Authors:** Laetitia G. E. Wilkins, Aude Rogivue, Frédéric Schütz, Luca Fumagalli, Claus Wedekind

**Affiliations:** 1 Department of Ecology and Evolution, Biophore, University of Lausanne, 1015 Lausanne, Switzerland; 2 WSL Swiss Federal Research Institute, Zürcherstrasse 111, 8903 Birmensdorf, Switzerland; 3 Center for Integrative Genomics, Génopode, University of Lausanne, 1015 Lausanne, Switzerland; 4 SIB Swiss Institute of Bioinformatics, Génopode, 1015 Lausanne, Switzerland

## Abstract

The taxonomic composition of egg-associated microbial communities can play a crucial role in the development of fish embryos. In response, hosts increasingly influence the composition of their associated microbial communities during embryogenesis, as concluded from recent field studies and laboratory experiments. However, little is known about the taxonomic composition and the diversity of egg-associated microbial communities within ecosystems; *e.g.,* river networks. We sampled late embryonic stages of naturally spawned brown trout at nine locations within two different river networks and applied 16S rRNA pyrosequencing to describe their bacterial communities. We found no evidence for a significant isolation-by-distance effect on the composition of bacterial communities, and no association between neutral genetic divergence of fish host (based on 11 microsatellites) and phylogenetic distances of the composition of their associated bacterial communities. We characterized core bacterial communities on brown trout eggs and compared them to corresponding water samples with regard to bacterial composition and its presumptive function. Bacterial diversity was positively correlated with water temperature at the spawning locations. We discuss this finding in the context of the increased water temperatures that have been recorded during the last 25 years in the study area.

Fish embryos are increasingly being used to study the interaction between a vertebrate and its associated bacteria, including for example: colonization by pathogenic bacteria^1^, maternal microbial transmission^2^, or the influence of micro-ecological factors on virulence of microbial symbionts^3^,^4^. Salmonid embryos, in particular, represent a powerful vertebrate model in ecology and evolution because they allow bridging laboratory experiments with field studies on a scale that can lead to quantitative conclusions^5^,^6^. Full-factorial *in vitro* breeding experiments using gametes collected from wild fish, and raising embryos under field conditions^7^ or under highly controlled conditions in the laboratory^8^,^9^,^10^ allow estimating the variance components (additive and non-additive genetic variance and maternal environmental effects) of important characteristics and hence the evolutionary potential of natural populations to adapt to environmental changes. Studies based on such an approach lead to a better understanding of maternal and genetic effects on (i) host susceptibility to pathogens at this early life stage^8^,^9^,^10^, (ii) different life-history strategies of embryos^8^,^10^,^11^, or (iii) the interaction of embryo hosts and their associated microbial communities^3^,^4^. This progress stands in sharp contrast to what is known about the diversity of host-associated bacteria in their natural environment. Here, we applied 16S amplicon sequencing to characterize the community structure of egg-associated bacteria and to identify factors that correlate with the composition of such communities.

We concentrated on brown trout (*Salmo trutta*) and sampled eggs from various natural spawning places. Most of these spawning places are located within the sub-alpine Aare river system that is characterized by different habitats and by significant genetic and morphological differentiation among brown trout populations^12^. Most of these populations have been markedly declining in abundance over the last decades^13^. Brown trout within the Aare river system have therefore been the focus of much research in the past^11^,^12^,^13^,^14^,^15^,^16^. We also sampled eggs from a spawning place that belongs to the Inn (>200 km away, Fig. 1), an alpine river that feeds into a different basin.

Brown trout bury their eggs in the gravel where they remain open for colonization by microbes. Among the factors that could potentially influence egg-associated bacteria and hence contribute to variation in symbiont bacterial communities on a regional scale are geographic distance between sampling locations (isolation by distance^17^,^18^), population-specific differences in maternal environmental effects (*i.e.,* egg characteristics^19^), variation in host genetics^7^,^11^,^15^ or water temperature. Water temperatures in the Aare river system have been monitored since 1970^13^,^14^. They have been increasing, in parallel to regionally increasing air temperatures^14^. Temperature is likely to affect the composition of bacterial community composition through its direct effects on metabolisms, indirect effects on generation time and mutation rates^20^, or accelerations and shifts in metabolic pathways^21^. Changes in temperature over time could therefore have affected the composition of bacterial communities on fish eggs.

We used 16S rRNA gene pyrosequencing to characterize the bacterial communities on naturally spawned eggs at their late-eyed stage; *i.e.,* on eggs that have been developing in their natural habitat over several weeks. We looked for correlations of the phylogenetic relationship of bacterial communities and geographic distances among different spawning locations, genetic differentiation of the brown trout host, and water temperature as measured during incubation of the eggs.

## Results

### Quality control

Ninety-one percent of raw sequence reads (38,049 out of 41,681) could be retained for downstream analysis after the different steps of quality control (Supplementary Table S1). A mean of 4,780 (SD = 1,165) sequences per spawning location was used to estimate bacterial alpha diversities and pairwise UniFrac bacterial community distances on brown trout eggs. After blasting these sequences against the Greengenes reference, a mean of 226 (SD = 105) operational taxonomic units (OTUs) could be assigned to each spawning location (Supplementary Table S1). Seventy-eight OTUs were found in the negative control of only beads and buffers (Supplementary Table S2). They were removed for downstream analysis. Raw read data are archived at the NCBI sequence read archive (SRA) under BioProject number PRNJA293374 and BioSample number SAMN04002383.

### Bacterial communities on brown trout eggs

**Figure 1:**
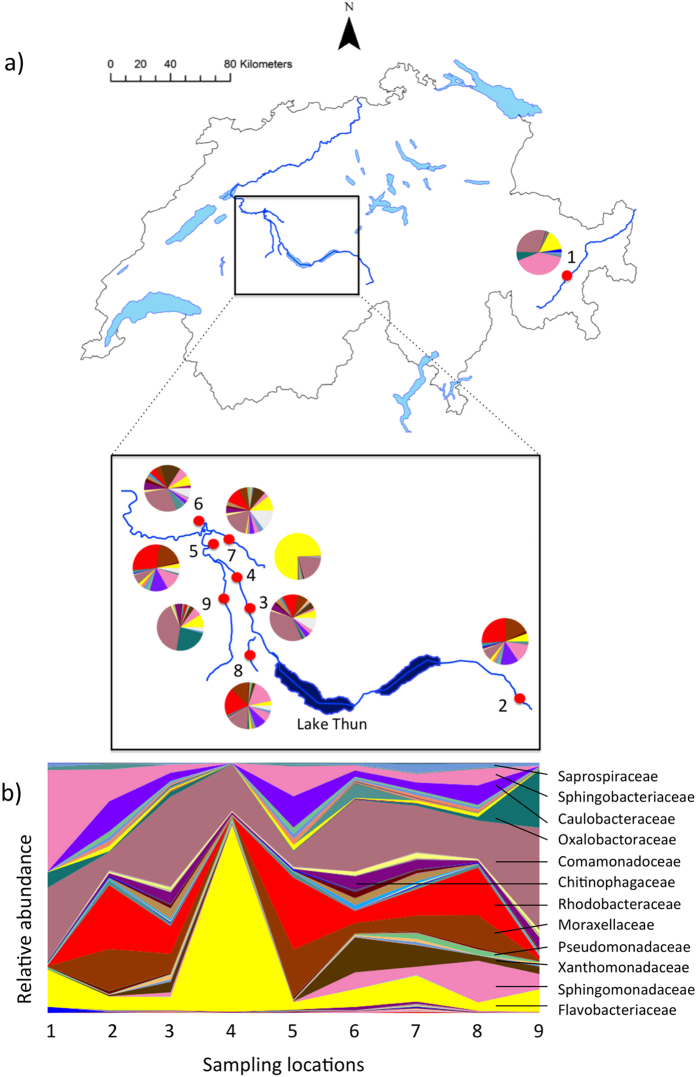
Sampling locations of bacterial communities on brown trout eggs at natural spawning places. Brown trout eggs were collected in two river systems within Switzerland: at Sils/Maria in the river Inn (sample-ID 1), as well as at eight spawning locations in the river Aare system. Five of these locations are in the main river Aare (2 = Aare-Innertkirchen, 3 = Aare-Wichtrach, 4 = Aare-Belp, 5 = Aare-Lerbestrasse, and 6 = Aare-Zehndermätteli) and three locations are tributaries of the river Aare (7 = Worble, 8 = Amletebach, and 9 = Gürbe). (**a**) Different colors in pie charts present the most common families of bacterial taxa at each spawning location. Identities of bacterial taxa for each color are shown in Fig. 2. Brown trout eggs were sampled at the late-eyed developmental stage, which is exemplified with one egg in this figure. This figure was drawn in ARCGIS 10.0^56^. (**b**) Bacterial composition on trout eggs at each spawning location is represented as relative abundances of the 12 most common bacterial families.

**Figure 2:**
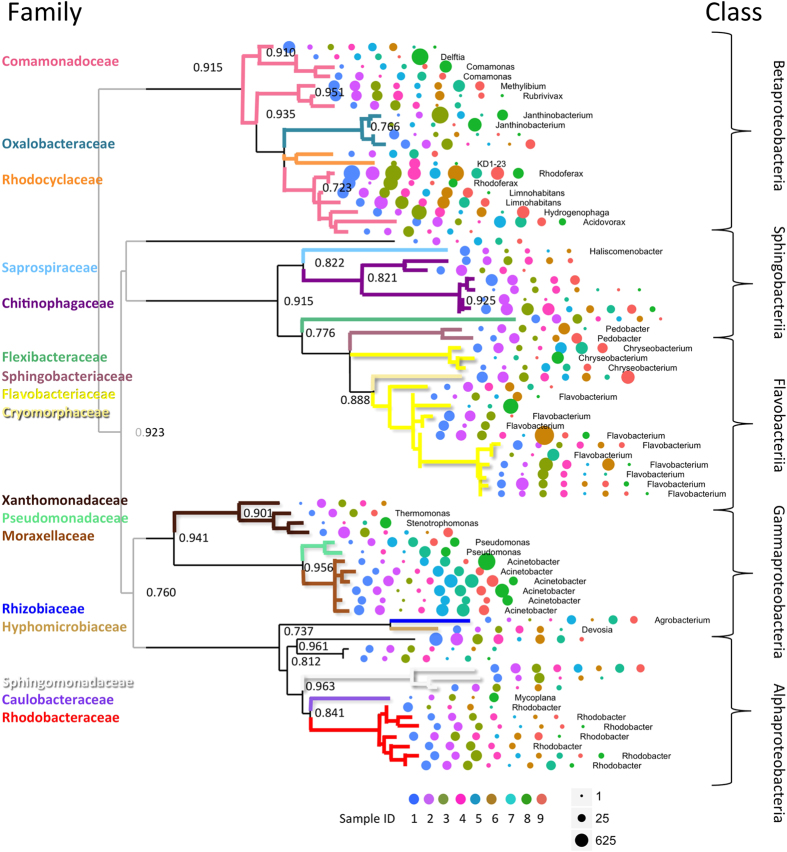
Neighbor-joining tree of core bacterial communities on naturally spawned brown trout eggs. The identity of each OTU is given at the genus level. Some bacterial taxa could not be identified at the genus level therefore the family identity is shown in different colors for each branch. Colors correspond to families in pie charts of Fig. 1. Multiples of the same names represent different bacterial sequences that matched to the same OTU reference in the online database (open blast search, see Material & Methods). Differently colored dots describe the nine spawning locations, while the size of the dot is relative to the abundance of a specific bacterial taxon at this location (see Fig. 1 and Table 1 for sample-IDs). Bootstrapping values (n = 10,000) >0.7 are shown at the nodes.

Three hundred seventy different OTUs could be differentiated in total. They were predominantly represented by *Bacteroidetes, Proteobacteria,* and *Firmicutes*. The eight most common families of bacteria on fertilized brown trout eggs at the late-eyed developmental stage were *Flavobacteriaceae, Comamonadaceae, Moraxellaceae, Enterobacteriaceae, Streptococcaceae, Oxalobacteraceae, Xanthomonadaceae, and Rhodobacteraceae* (Fig. 1, Supplementary Figs S1 and S2). Identities of all bacterial taxa after comparing each sequence to an online reference (Greengenes and RDP classifier) are listed in Supplementary Table S3. The most common bacterial families at each spawning location are shown in different colors in pie charts and area charts in Fig. 1. Rarefaction curves of three different bacterial alpha diversity measures at each spawning location (Chao 1, number of observed species, and phylogenetic distance) are shown in Supplementary Fig. S3 (rarefaction curves of bacterial alpha diversity measures without removal of sequences that had been found in the negative control of only beads and buffers are shown in Supplementary Fig. S4). In contrast to unfiltered alpha diversity measures of bacterial diversity on brown trout eggs, rarefaction curves of alpha diversity measures that went through a stringent quality control did not reach saturation. Mean bacterial alpha diversity measures on brown trout eggs at each spawning location are listed in Table 1 (unfiltered alpha diversity measures are shown in the Supplementary Table S4). Core bacterial taxa (n = 75) that were found at seven out of nine natural brown trout spawning locations were built into a neighbor-joining tree (Fig. 2). The number of bacterial taxa that could be found at different fractions of samples is shown in Supplementary Fig. S5. Contrasts in the putative, functional potential between bacterial communities on brown trout eggs and corresponding water samples are shown in Supplementary Fig. S6. KEGG pathways that were significantly more abundant in brown trout egg-associated bacterial communities (*p* < 0.05 after Benjamini – Hochberg multiple testing correction controlling the FDR) are listed in Table 2. Prokaryotic metabolic pathways in this list included for example folate biosynthesis, carbon fixation, biosynthesis of antibiotics, and amino sugar and nucleotide sugar metabolism (Table 2).

### Spatial pattern of bacterial community composition

**Figure 3:**
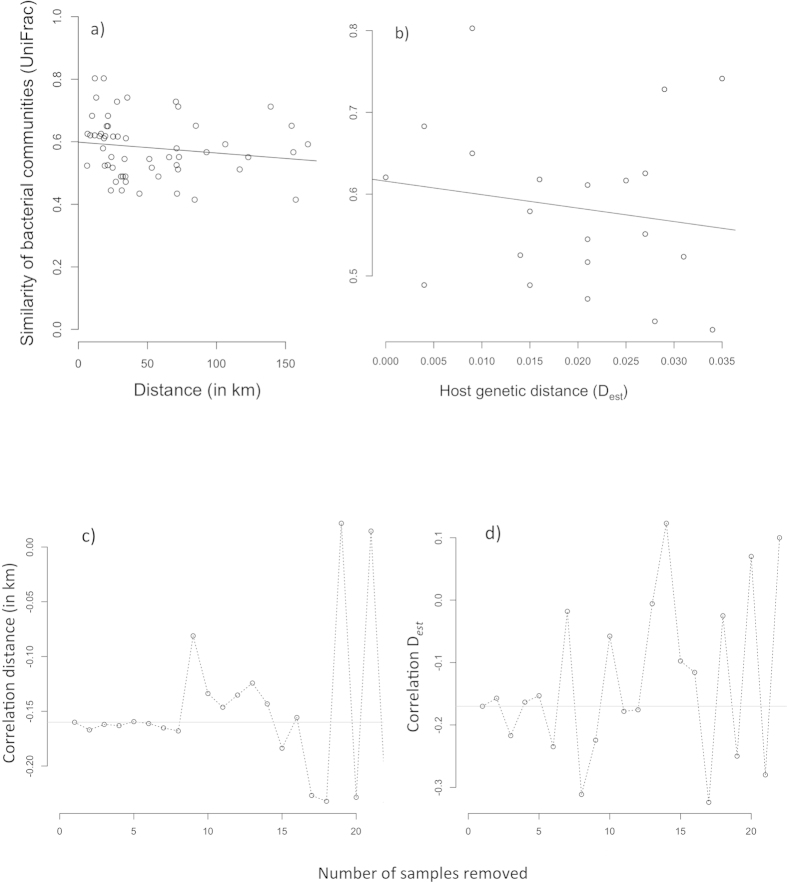
Spatial pattern of bacterial community composition on brown trout eggs in the Aare system. Relationship of pairwise UniFrac bacterial community phylogenetic distances on brown trout eggs at natural spawning places in the Aare system with (a) corrected geographic distances (km) or (b) host genetic differentiation (*D*_*est*_). We did not find evidence for a significant relationship in both cases with *p* ≤0.05. (**c, d**) We sequentially decreased the number of pairwise comparisons (randomly taking out one sample, than randomly two samples, then randomly three samples, and so on) and calculated the resulting slope of the relationship in order to examine the effect of sample size on correlation estimates for the similarity of bacterial communities (UniFrac) with (**a**) corrected geographic distance (in km) or (**b**) host genetic distance (D_*est*_).

**Figure 4:**
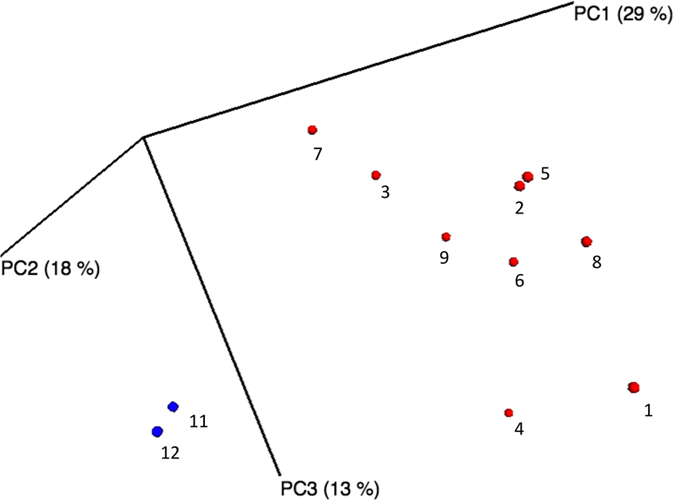
Three-dimensional PCoA plot of UniFrac distances between bacterial communities on water samples and brown trout eggs. A Principal Coordinate Analysis plot was drawn in order to visualize phylogenetic distances between bacterial communities on brown trout eggs and two corresponding water samples. Numbers correspond to spawning locations in Fig. 1, Table 1, and Supplementary Table S1; blue = water samples, red = brown trout egg samples.

Geographic distances among spawning locations within the Aare system were not correlated with phylogenetic distances of bacterial communities (UniFrac) on brown trout eggs using neither Mantel statistics (*r* = −0.39, *p* = 0.19) nor partial Mantel statistics while controlling for variation in water temperature (*r* = −0.17, *p* = 0.21; Fig. 3a). No sign of isolation-by-distance could be detected. Corrected geographic distances and UniFrac distances of bacterial communities did not result in any significant improvement of the spatial relationship (*r* = −0.16, *p* = 0.25). This result did not change when unfiltered UniFrac distance measures were used (see Supplemental Information and Supplementary Fig. S7a). However, slope estimates for the correlation of corrected geographic distances with UniFrac distances of bacterial communities were robust using the number of pairwise comparisons we did (Fig. 3c). Correspondence analysis of genetic differentiation among different brown trout populations (*D*_*est*_) and UniFrac bacterial community distances did not result in a significant association (*r* = −0.17, *p* = 0.29; Fig. 3b). Again, results did not change when unfiltered UniFrac distance measures were used (see Supplemental Information and Supplementary Fig. S7b). Here, slope estimates for the correlation of host genetic distance with UniFrac distances of bacterial communities were highly variable and did not converge given the number of pairwise distances we used (Fig. 3d). All distance matrices are listed in Supplementary Table S6 (including unfiltered UniFrac distances that had been estimated without the removal of sequences from the negative control). The composition of bacteria at spawning locations in the main river Aare was not significantly different from the bacterial communities on brown trout eggs in three of its tributaries (adonis: *p* = 0.79, R^2^ = 13%; ANOSIM: *p* = 0.7, R^2^ = 14%), while there were no deviations from the assumption of equal variances of bacterial composition between the two groups (*F* = 0.15, d.f. = 1, *p* = 0.72). Increasing the number of samples within the two groups sequentially using a bootstrapping approach did not change this result (Supplementary Methods, Supplementary Table S7). The bacterial community on brown trout eggs at a spawning location in the river Inn represented a subsample of the bacterial taxa found in the river Aare system (Fig. 1, Table 1, Supplementary Fig. S1, S2). This bacterial community differed in a mean UniFrac bootstrapping distance of 0.573 (95% CI: 0.558; 0.588) from the bacterial communities in the river Aare, while the observed UniFrac distance was 0.583. The probability of obtaining the observed distance in the simulated river Inn bacterial communities was 0.42. Principal Coordinate Analysis (PCoA) plots were created in EMPeror v.0.9.3^22^ in order to visualize the bootstrapping analysis comparing one bacterial community on brown trout eggs in the river Inn with bacterial communities of the river Aare (Supplementary Fig. S8), and to contrast phylogenetic distances between bacterial communities on brown trout eggs and corresponding water samples (Fig. 4). The same PCoA plot of variation in bacterial communities in water samples and on brown trout eggs was drawn without the removal of sequences that had been found in the negative control of only beads and buffers (Supplementary Fig. S9). In the river Gürbe (location 9), the bacterial community of the water sample differed in a UniFrac distance of 0.715 from the bacterial community of its corresponding brown trout egg samples, while the bootstrapping analysis resulted in a mean UniFrac distance of 0.573 (95% CI: 0.558; 0.588). The probability of obtaining the observed UniFrac distance was less than 0.001. At Aare-Lerbestrasse (location 5) the bacterial community of its water sample differed in a UniFrac distance of 0.765 from the bacterial community of its corresponding brown trout egg samples, while the bootstrapping analysis resulted in a mean UniFrac distance of 0.533 (95% CI: 0.516; 0.549). The probability of obtaining the observed UniFrac distance was again less than 0.001.

### Water temperature and bacterial community composition

**Figure 5:**
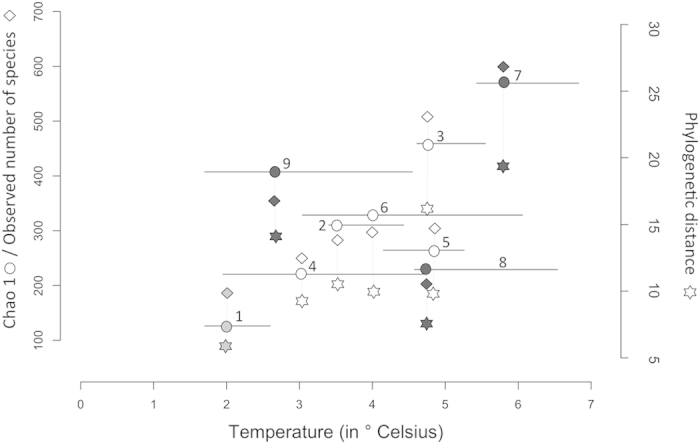
Relationship of water temperature and bacterial alpha diversities on brown trout eggs at natural spawning places. Circles = Chao 1, diamonds = observed number of species, and stars = phylogenetic distance (grey = river Inn, black = tributaries of the river Aare, and white = spawning locations in the main river Aare; see Fig. 1 and Table 1 for location names). The three different alpha diversity measures at each spawning location are connected by a dotted line while the mean incubation water temperature goes through the Chao 1 value. A solid horizontal line shows the upper and lower quartiles in mean water temperature. The range of Chao 1 and observed number of species is shown on the left y-axis and the range of phylogenetic distance is shown on the right y-axis. All spawning locations from both river systems are included.

**Figure 6:**
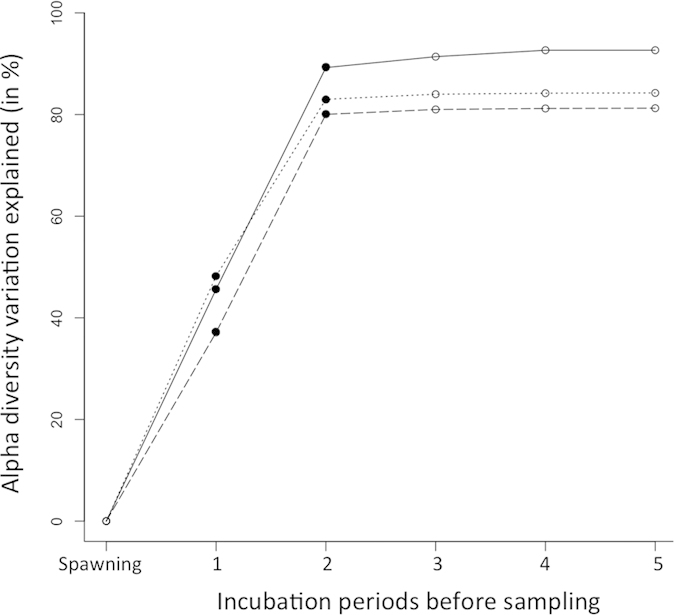
Results of multiple regression analysis on the association of average water temperature during five consecutive time periods of incubation and bacterial alpha diversities on brown trout eggs. Variation in alpha diversity explained by adding the average water temperature of five time periods between spawning and sampling of the eggs to a forward stepwise regression (black circles: models significantly improved by adding the latest covariate, white circles: no significant model improvement; *F* always >2 and p always <0.01). The alpha diversity measures are Chao 1 (solid line), observed number of species (dotted line), and phylogenetic distance (dashed line). The whole dataset including all nine spawning locations was used for this analysis.

All three alpha diversity measures of brown trout egg-associated bacterial communities significantly increased with the average water temperature at the spawning locations during the whole incubation time: Chao 1: *τ* = 0.61, *z* = 2.05, *p* = 0.03; observed number of species: *τ* = 0.56, *z* = 1.79, *p* = 0.04; and phylogenetic distance: *τ* = 0.67, *z* = 2.38, *p* = 0.02 (Fig. 5). The same significant pattern was found using alpha diversity measures of brown trout egg-associated bacterial communities without removal of sequences that had been found in the negative control of only beads and buffers (see Supplemental Information and Supplementary Fig. S10). Bacterial community distances (UniFrac) were significantly correlated with variations in water temperature while controlling for geographic distances among spawning locations within the Aare river system (*r* = 0.381, *p* = 0.002). When the incubation time of the eggs was divided into five equal time periods from the day of spawning until the day of sampling, the average water temperatures of the first two periods or a variable that correlates with it, was sufficient to explain most of the variation in alpha diversity of bacterial communities (Chao 1 *F*_2,6_ = 24.88, *p* = 0.001; observed number of species *F*_2,6_ = 12.32, *p* = 0.007; and phylogenetic distance, *F*_2,6_ = 15.3, *p* = 0.004; Supplementary Table S8, Fig. 6). Specific associations of bacterial taxa with their salmonid hosts were inferred with a search in Web of Knowledge^TM^ v.5.12 (Thomson Reuters) or according to Austin & Austin^23^.

## Discussion

In this study we characterized typical bacterial communities at natural spawning places of brown trout. We found no evidence for a correlation of bacterial phylogenetic distances among spawning places to either geographic distances or genetic differentiation of the brown trout populations. Instead, bacterial diversity was positively correlated to the water temperature during the incubation time of the eggs.

If bacterial communities are specifically associated with environmental variables they are expected to differ between habitats^17^. Such ecological specializations can come into play even on a small geographic scale^24^. We did not find evidence for significant differences in the bacterial community composition of the main river Aare and its tributaries. Moreover, naturally spawned eggs at one brown trout spawning location in the river Inn harbored the same main bacterial groups as eggs in the Aare although the two rivers belong to different basins (Danube and Rhine), and the spawning places differ with regard to altitude. On the contrary, the bacterial composition of two water samples that had been collected simultaneously at two spawning places in the Aare was markedly different from its corresponding brown trout egg-associated bacterial communities. Consequently, the consistent composition of core bacteria on brown trout eggs from various populations and differing habitats supports the idea of a substrate-specific microbiome^25^. Bacterial members of this core community seem to represent a subsample of mainly ubiquitous environmental bacteria from the specific spawning habitat in the riverbed. Although we did not find evidence for a significant correlation of geographic and phylogenetic distance of bacterial communities between spawning locations, our slope estimates for this relationship converged; *i.e.,* bacterial communities differed maximally by 5% when 150 km apart from each other. This is a relatively low isolation-by-distance pattern when compared to other bacterial ecosystems^26^. Distinct microbiomes are shaped by local environmental or biological factors that result in similar bacterial communities over large geographic scales^17^. For naturally spawned trout eggs these factors could include early maternal effects, such as antimicrobial compounds that trout females allocate to their eggs before spawning^19^, as well as specific interactions between the bacterial communities and their trout embryo host at later developmental stages; *e.g.,*^11^,^15^. Additionally, environmental variables that are shared among trout spawning places, such as oxygen dynamics or sediment deposition^27^ might drive the bacterial community composition on trout eggs.

Members of this core community of bacterial taxa on brown trout eggs that matched to a previously reported reference comprised different genera of the bacterial families of Flavobacteriaceae and Comamonadaceae. The former family was mostly represented by the genus *Flavobacterium*. Flavobacteria are commonly found in freshwater systems^23^. At warmer temperatures we found 16S sequences that matched to *Flavobacterium psychrophilum,* a fish pathogen that causes bacterial cold water disease (BCWD) in different adult salmonid species, and rainbow trout fry disease (RTFS) in *O. mykiss*^23^. Within the family of Commamonadaceae, bacterial sequences of the genus *Comamonas* were highly represented. Other genera of the same bacterial family contributed also with plenty of sequences, such as *Rhodoferax*, *Hydrogenophaga*, *Limnohabitans*, and *Delftia*. *Rhodoferax* species are frequently found in aquatic systems that are exposed to light, but also in ditchwater and activated sludge^28^. They are members of purple non-sulfur bacteria, a polyphyletic group of phototrophic bacteria. *Hydrogenophaga* are ubiquitous free-living freshwater planktonic bacteria^29^. *Delftia* is a bacterial genus that has formerly been classified within the family of Pseudomonadaceae^28^. It is strictly aerobic and a common water and soil saprophyte of limited virulence. However, it can develop into an opportunistic freshwater fish pathogen^30^. Another opportunistic freshwater fish pathogen that was found in the bacterial community composition of naturally fertilized brown trout eggs was the genus *Pseudomonas*. This genus harbors several species that may act as fish pathogens^23^, such as *P. fluorescens*, an opportunistic brown trout pathogen that has been shown to reduce embryonic survival and performance^11^,^15^. However, *P. fluorescens* can also be found in fish and environmental samples without any sign of disease^28^. Moraxellaceae was represented by different species or strains of the genus of *Acinetobacter*. Species of this genus have been described to be strictly aerobic, non-fermentative bacilli^28^. They are oxidase-negative, non-motile, and usually nitrate-negative. In water they form aggregates^28^, a property that could explain why they were so common in bacterial communities associated with brown trout eggs in our dataset. Another bacterial group that contributed many 16S sequences was *Janthinobacterium*, a genus from the family Oxalobacteraceae. This genus of commensalists comprises different species that can inhabit the gastrointestinal tract of vertebrates, including freshwater fish^23^. They have a distinctive dark-purple color due to a compound called violacein^31^. This compound has anti-fungal properties. *J. lividum* is found on the skin of amphibians including the red-backed salamander (*Plethodon cinereus*), where it prevents infection by the chytrid fungus (*Batrachochytrium dendrobatidis*)^31^. Cryomorphaceae is a family of filamentous bacteria^28^. Their optimal growth occurs at low water temperatures (−2 °C–22 °C)^23^. The ability to grow optimally at this temperature range has also been described for the genus *Devosia* of the bacterial family Hyphomicrobiaceae^32^. Caulobacteraceae are common in oligotrophic freshwater lakes^23^. They have a dimorphic life cycle and can build prosthecae that they use to attach to a specific substrate, such as for example fish eggs.

Many bacterial sequence reads that we found on naturally fertilized brown trout eggs seem to represent ubiquitous bacteria that are typically associated with freshwater environments^33^. They included bacteria of the genus *Stenotrophomonas*, *Leuconostoc*, the bacterial families Chitinophagaceae and Flexibacteraceae, the genera *Haliscomenobacter, Pedobacter*, *Rhodobacter*, and the family Rhodocyclaceae. Raw counts of sequences for each bacterial group are shown in the Supplemental Information. However, these numbers cannot be taken as direct estimates of bacterial abundance because we did not estimate cell counts for any bacterial group. While traditionally fish embryo-associated bacteria have been investigated in the light of pathogen infection and disease transmission, there is recent evidence that bacterial communities on fish eggs can also act as a protective barrier^4^. Following-up studies targeting specific, egg-associated bacterial groups and applying controlled incubation experiments are needed to determine the impact of host-associated bacteria and their potentially beneficial roles. Here, we used PICRUSt to predict the functional potential of brown trout egg-associated core bacterial communities. This is not a perfect substitute for metagenomic sequencing or experimental studies, but it allows the development of initial hypotheses. Predicted, synthetic metagenomes of brown trout eggs harbored markedly enriched genes of folate biosynthesis, carbon fixation, biosynthesis of antibiotics, biosynthesis of essential amino acids, and micro pollutant degradation. All of these pathways could turn out beneficial for developing trout embryo hosts. Yet, the goal of this study was not to explore the explicit effects of brown trout egg-associated bacterial communities but to describe their core composition and their spatial distribution in a river system.

Bacterial sequences in our dataset were confined to a length of 311-bp long reads, which limited our ability to determine many bacteria to a species level. Nevertheless, we set a starting point for the investigation of an interaction between naturally spawned brown trout embryos and their associated bacterial communities. We applied a stringent quality control to raw 454 sequencing reads. Briefly, sequences needed to: (i) have a perfect match to their barcode and the 16S rRNA primer, (ii) be at least 300-bp long, (iii) have no more than two undetermined bases, (iv) have at least a 60% match to a 16S rRNA gene sequence from the reference database. Furthermore, sequences were filtered out that represented chimeras. As a last step of quality control, all sequences were removed that had been detected in the blank control sample of only beads and buffers. Our stringent quality control resulted in fewer reads to characterize bacterial communities and alpha diversity measures. Accordingly, rarefaction curves of filtered alpha diversity measures did not reach saturation in contrast to unfiltered alpha diversity measures. However, unfiltered estimates of bacterial alpha diversity measures and phylogenetic distances among samples revealed the same general pattern as filtered estimates. When we specifically looked at bacterial sequences that had been found in the negative control of only beads and buffers, we found many bacterial taxa that are commonly found on humans. These taxa included for example a vast array of sequences that are specifically found in milk (*e.g., Lactococcus* spp.) or human commensalists (*e.g., Enterococcus faecalis, Prionibacterium acnes, Staphylococcus epidermis, Streptococcus pseudopneumonia,* and *Veillonella* spp.). Consequently, given the congruence of filtered and unfiltered results, and the possible human origin of contaminants, we considered it appropriate to rely on the most stringent quality control chosen.

Bacterial alpha diversity at the different spawning locations was positively correlated to local water temperature. All three alpha diversities consistently increased with incubation temperature. Direct effects of temperature or a covariate of temperature on metabolism and indirect effects on generation time and mutation rates are likely responsible for the distribution of bacterial diversity^21^ on brown trout eggs in this study in two very different, not connected river systems: a moderately warm, sub-alpine region and a cold alpine location at higher altitude.

When the incubation time was split up into five consecutive time periods, the average temperature of the first two periods after spawning was sufficient to explain most of the variation in bacterial diversity on brown trout eggs. This finding suggests that temperature or a factor that correlates with temperature at the time of bacterial colonization relates to bacterial community composition on naturally fertilized brown trout eggs. It has been shown in other systems that bacteria form a core community shortly after colonization, which is quite resilient to disturbances thereafter^34^. This might apply to the trout egg-associated bacteria as well.

The water temperature in the Aare system has increased over the last 40 years^14^. With regard to the host, warming can result in an altitudinal upward shift of optimal habitats^35^. Migration to higher altitudes is restricted by physical barriers in the Aare system, therefore a temperature increase implies a habitat reduction for many fish populations^14^. Studies in wild salmonid populations have shown that naturally occurring pathogenic bacteria can maintain diversifying selection pressure on their host’s immune system^36^. This selection pressure can vary in intensity based on pathogen richness, pathogen virulence, and the length of the cohabitation period. All of these factors tend to increase with temperature^37^.

Repeatable patterns in microbial community composition have been revealed in rivers, lakes, and oceans, providing evidence of a consistent core microbial community in specific habitats^24^,^25^,^34^. Here, we add the characterization of core bacterial communities on naturally spawned brown trout eggs at the late-eyed developmental stage. Egg-associated bacteria were notably different from the bacteria in the water surrounding them. We did not find evidence of a correlation between bacterial community composition and geographic distance among spawning places or host genetic differentiation. Yet, bacterial diversity on naturally spawned brown trout eggs increased with incubation water temperature. It remains to be tested whether such temperature-linked changes in bacterial communities can contribute to the decline of host populations.

## Material and Methods

### Sample acquisition

Naturally spawned brown trout eggs were collected from five different locations within the river Aare (belonging to the Rhine basin that feeds the North Sea), three locations in tributaries of the river Aare (Amletebach, Gürbe, and Worble), and one location in the river Inn (belonging to the Danube Basin that feeds the Black sea; Fig. 1, Table 1). Spawning locations in the Aare system were all located between 500 to 600 m.a.sl., whereas the one spawning location in the river Inn lies above 1800 m.a.s.l. The two river systems do not only differ with regard to altitude but accordingly also with regard to mean water temperature (Table 1). Sampling of eggs in the field was performed in accordance with all relevant guidelines and regulations. Fieldwork and all experimental protocols were approved by the Fishery Inspectorates of the Bern canton and the Graubünden canton. Additional authorizations from the cantonal veterinary offices were not required as all manipulations on embryos were performed prior to yolk sac absorption. Eggs were extracted from the gravel using the same rake for the whole study. After excavation, eggs were collected with sterilized sieves and plastic pipettes (Sigma-Aldrich, Buchs). After collection, eggs were immediately rinsed with 2 L of autoclaved and filtered (0.2 μm, Millipore, Zug, Switzerland) water. Embryo developmental stage was determined on a sterile glass slide under a field light microscope (Motic Microscopes 1820, Wetzlar, Germany). Eggs were then frozen in liquid nitrogen and later stored at −80 °C without a storage buffer. Only fertilized eggs at the late-eyed stage were used for further analysis (sampled between 12.02. - 02.03.2010, Table 1). In order to sample different host types, eggs from at least four different trout spawning nests (redds) were included. Consequently, we sampled eight eggs from each spawning location for individual DNA extractions. Details about sampling are elaborated in the Supplemental Information. At two spawning locations (Aare-Lerbestrasse and Gürbe; *i.e.,* locations 5 and 9; Fig. 1, Table 1), 1.5 L water was collected in a sterile glass bottle as close as possible to the location where eggs had been found. This water was filtered (0.2 μm, Millipore, Zug, Switzerland), and the filters were stored at −80 °C (without storage buffer) until bacterial DNA extraction. Temperature loggers (HOBO Water Temperature Pro v2 Data Logger – U22-001, Bakrona Zürich) were installed at every location around the beginning of the spawning season. Temperature was measured continuously at intervals of 10 minutes.

### Molecular genetic analyses

DNA extractions and PCR conditions are described in detail in the Supplemental Information. Briefly, a bead beating protocol was applied to extract total bacterial DNA, and PCR was performed with a bacterial-specific primer pair, 27F and 338R, that amplifies a 311-bp long fragment of the V1-V2 hypervariable region of bacterial 16S rRNA^38^. The 338R primer included a unique 10-bp long sequence tag to barcode each sampled location (Supplementary Table S1). Eight individual bacterial DNA extractions were pooled for every spawning location resulting in a total of nine PCR reactions representing the nine different spawning locations, two PCR reactions for water samples, and one PCR reaction for the negative control that consisted of all reagents except the extracted DNA. Eight different bacterial DNA extractions representing different redds were pooled in an attempt to capture bacterial diversity within a spawning location. All PCR reactions were pooled in equimolar amounts (at a concentration of 15 ng/μL DNA) into a 10 mM Tris-HCl buffer at pH 8.5 and sent for 454 pyrosequencing (Microsynth, Balgach, Switzerland) of the 311-bp fragment on a Genome Sequencer FLX System (Roche, Basel, Switzerland).

### Bacterial community comparison

The quality control pipeline, that was applied to raw 454 pyrosequencing reads, is described in the Supplemental Information (see also^39^). Particularly noteworthy is the filtering step where we removed all bacterial sequences that had been found in the negative control of only beads and buffers from the remaining samples (Supplementary Table S2). All resulting reads (Supplementary Table S3) were used for further analysis. Statistical analyses were done in the QIIME framework v.1.6.0^40^. Operational taxonomic units (OTUs) were picked using the Usearch algorithm 5.2.236^41^ with default parameters. This algorithm assigns similar bacterial sequences to OTUs by clustering them based on a user-defined similarity threshold (sequence similarity was set to 0.97, roughly corresponding to species-level OTUs^42^). In every OTU cluster the most abundant sequence was chosen and then assigned to a reference using an open blast search with the RDP classifier 2.2^43^ and the Greengenes reference database v.12.10^44^. This approach enables the identification of previously undescribed bacterial sequences^40^. The composition of bacterial taxa on trout eggs and in water samples at natural spawning places was represented using barplots and heatmaps showing relative bacterial abundances at different resolutions (n of bacterial taxa in barplots = 37, n of bacterial taxa in heatmaps = 120) using the R package ‘phyloseq’ v.1.7.12^45^ in the R environment v.3.1.3^46^. Bacterial community samples in heatmaps were ordered according to non-metric multidimensional scaling^47^. In order to inspect the effect of removing sequences that had been found in the negative control of only beads and buffers from all the remaining samples, bacterial compositions were also illustrated without the removal of sequences.

The following three alpha diversity measures were calculated for each spawning location: Chao 1, number of observed species, and phylogenetic distance^48^. Alpha diversities were visualized using rarefaction curves. More than one alpha diversity index was included in the analysis because they quantify diversity based on different assumptions^48^.

UniFrac distances were calculated to quantify differences in alpha diversity measures and phylogenetic distances among bacterial communities^49^. Unweighted UniFrac distances were applied for the downstream analysis using only OTUs presence or absence information. Quantitative measures (weighted UniFrac) are suited for revealing community differences that are due to changes in relative taxon abundance; *e.g.,* when a particular set of taxa flourish because a limiting nutrient source becomes abundant^49^. Qualitative measures (unweighted UniFrac) are most informative when communities differ primarily by what can live in them; *e.g.,* at high temperatures or on different hosts, in part because abundance information can obscure significant patterns of variation in which taxa are present. Alpha diversity measures, rarefaction curves, and UniFrac distances were also estimated for unfiltered bacterial communities.

To build a phylogenetic dendrogram, OTUs were aligned using the PyNAST algorithm v.1.2.2^50^ and a phylogenetic tree was built with FastTree v.2.0^51^, in the QIIME framework using default parameters.

In order to calculate a core microbiome of brown trout eggs at their natural spawning places, bacterial taxa were identified that are present in >77% of all samples; *i.e.,* at seven out of nine spawning places. We used the QIIME script compute_core_microbiome.py for this selection. To graphically represent typical egg-associated bacterial communities, a phylogenetic neighbor-joining tree was built with phyloseq in R. Based on the core microbiome on brown trout eggs, a synthetic metagenome was generated with PCIRUSt v.1.0.0^52^ using the online Galaxy version. Briefly, there is evidence for a correlation between the evolutionary relatedness of two bacterial taxa and the similarity of their genomic functions^52^. In cases where metagenomic sequencing is unfeasible due to the overrepresentation of host DNA relative to bacterial DNA, as it is the case here with trout eggs, bacterial taxa can be inferred by 16S rRNA sequences, which will then be used to predict their functional potential. This predictive approach has recently been implemented in the software PICRUSt. This software uses the phylogenetic placement of a 16S rRNA sequence within a phylogeny to infer the content of the genome of an organism of interest hat is only represented by a 16S rRNA sequence.

First, the core microbiome was normalized with respect to inferred 16S rRNA gene copy numbers. This normalized OTU table was then used to predict microbial metagenomes on trout eggs. The same procedure was applied to two corresponding water samples. Predicted metagenomes of the two groups were analyzed with STAMP v.2.1.2 to visualize presumable functions of the synthetic metagenome^53^. Gene functional categories were derived from the KEGG database. To test for significant differences between the predicted, synthetic metagenomes of brown trout eggs and corresponding water samples, we used Welch’s two-sample t-test for unequal sample sizes and variances. We applied a Benjamini – Hochberg multiple testing correction controlling the FDR and the threshold was set as FDR value <0.05. As the number of water samples was much lower than brown trout egg samples, we only compared gene copies that were present in both synthetic metagenomes.

Non-parametric analyses of variance were performed in order to investigate if bacterial communities on brown trout eggs from the main river Aare downstream Lake Thun (locations 3–6, Fig. 1) were different from the bacterial communities of three of its tributaries (locations 7–9, Fig. 1) with regard to pairwise UniFrac distances using the ‘vegan’ package v.2.2.1^54^ with the implemented functions ‘adonis’ and ‘ANOSIM’ in R. These analysis of variance methods are non-parametric but they assume equal variances among groups of samples. These tests were two-sided. They are described in further detail in the Supplemental Information. The function ‘Permdisp’ in the same package was applied to test for equal variances of bacterial composition among spawning locations. Significance and *p*-values were obtained by permutation (n = 99,999; see Supplemental Information). *P* was considered significant if ≤0.05.

### Bootstrapping analysis

If one bacterial community is compared to several other bacterial communities, the assumption of equal variances among groups is violated^55^. Therefore we adapted a second approach to compare bacterial communities that is based on bootstrapping^39^. Applying the statistical procedure of re-sampling with replacement, we tested if the bacterial community at the spawning location in the river Inn was different from the bacterial communities at spawning places in the main river Aare system (locations 3–6, Fig. 1). Bootstrapping was done in R and is further described in the Supplemental Information. The same analysis was applied to compare bacterial communities of water samples to their corresponding bacterial communities on brown trout egg samples. Bootstrapping was also applied to estimate the power of the non-parametric analysis of variance test between bacterial communities on trout eggs in the main river Aare and its tributaries with regard to sample size within groups; *i.e.,* to assess whether the observation we made was representative of the results we can expect in general (Supplemental Information).

### Spatial pattern of bacterial community composition

Mantel tests and partial Mantel tests were used to investigate correlations of geographic distances between the different spawning locations and pairwise UniFrac bacterial community distances, as implemented in the ‘ecodist’ package v.1.2.9^55^. *P*-values were obtained from permutation tests (99,999 permutations). *P* was considered significant if ≤0.05, and these tests were one-sided. Only spawning locations from the river Aare were included in this analysis (n = 8). Using Mantel testing^55^ we assessed if there was a linear relationship between pairwise UniFrac distances of egg-associated bacterial community and geographic distances. These geographic distances were extracted from geographic information system (GIS) data and measured in ArcGIS 10.0^56^. Partial mantel tests were used to test for associations of geographic distances and UniFrac bacterial community distances among different spawning places in the river Aare system, while controlling for mean local water temperature. All analyses on spatial relationships were applied to spawning locations in the Aare river network only (locations 2–9). Additionally, the same analyses on spatial relationships were also applied to pairwise UniFrac bacterial community distances without removing sequences that had been found in the negative control of only beads and buffers from all the remaining samples.

Geographic distances between spawning places might not be an accurate distance estimate due to the variable water flow in a river system^57^. The impact of water flow in the river may typically be strong enough that it affects the energy investment of a migrating fish. To account for this problem we applied a correction to geographic distances. The correction is explained in the Supplemental Information.

A previous study on the genetic diversity of brown trout revealed significant genetic and phenotypic variation in the same river system^12^. The correspondence of genetic differentiation among different brown trout populations (*D*_*est*_) and pairwise UniFrac bacterial community distances was calculated with a Mantel test. *P* was considered significant if ≤0.05, and these tests were one-sided. Briefly, global genetic diversity and pairwise population differentiation (*F*_*ST*_) were calculated according to Weir & Cockerham^58^. They were based on 11 microsatellites (in n = 20–36 individuals for each sampling site/population; for detailed information see Stelkens *et al.*^12^). Since *F*_*ST*_ estimates have been shown to depend on the level of mean heterozygosity within populations and accordingly decline with increasing polymorphisms^59^,^60^, we used the ‘actual differentiation’ estimator *D*_*est*_^59^ in order to estimate genetic differentiation of trout populations among different sampling sites. The measure of *D*_*est*_ integrates a weighting treatment where all populations are scaled by both their size and their average within-subpopulation heterozygosity. Moreover, the correspondence of D_*est*_ among different brown trout populations and bacterial community distances was also calculated analogously using unfiltered pairwise UniFrac bacterial community distances without removing sequences that had been found in the negative control.

In order to examine the effect of sample size on correlation estimates, we sequentially decreased the number of pairwise comparisons (randomly taking out one sample, than randomly two samples, then randomly three samples, and so forth) and calculated the resulting slope of the relationship between phylogenetic distances of bacterial communities on brown trout eggs (UniFrac) and corrected geographic distances or host genetic distances respectively.

### Relationship between temperature and bacterial community composition

Kendall’s rank-order correlation coefficient (*τ)* was used to investigate the relationship between local water temperature at the spawning locations and alpha diversity measures of egg-associated bacterial communities. Partial Mantel tests were used to test for an association of the variation in temperature and UniFrac bacterial community distances while controlling for geographic distances. *P* was considered significant if ≤0.05, and these tests were one-sided. All spawning locations were included in these analyses (n = 9). The same analyses on the relationship between local water temperature at the spawning locations and alpha diversity measures of egg-associated bacterial communities were also applied without removing sequences that had been found in the negative control. A multiple regression analysis was applied relating bacterial diversity to the average water temperature on the sampling date and to previous temperature averages during the incubation time of the eggs. The time of incubation from spawning until sampling of the eggs was therefore divided into five consecutive periods (equal length in terms of calendar days) that were used as explanatory variables in the model. We also performed a forward stepwise regression analysis predicting alpha diversity measures of brown trout egg-associated bacterial diversities on the basis of the average water temperature during those five consecutive time periods.

## Additional Information

**How to cite this article**: Wilkins, L. G. E. *et al.* Increased diversity of egg-associated bacteria on brown trout (*Salmo trutta*) at elevated temperatures. *Sci. Rep.* **5**, 17084; doi: 10.1038/srep17084 (2015).

## Supplementary Material

Supplementary Information

## Figures and Tables

**Table 1:** Spawning locations and alpha diversity measures of bacterial communities on naturally spawned brown trout eggs.

Sample-ID	Location	GPS coordinates	Elevation (m. a. s. l.)	Mean T^a^	PD^b^	Chao 1^c^	OS^d^
1	Inn	46°26′04.05”	1802	2.00/0.60	8.59	126.2	91
		9°45′19.21”					
2	Aare-Innertkirchen	46°42′16.71”	636	3.51/0.22	14.88	310.33	205
		8°14′02.38”					
3	Aare-Wichtrach	46°50′02.54”	532	4.75/0.29	24.24	459	340
		7°33′53.73”					
4	Aare-Belp	46°53′50.61”	517	4.73/0.30	11.68	229	133
		7°31′05.93”					
5	^*^Aare-Lerbestrasse	46°56′59.62”	503	4.85/1.40	15.22	264.54	187
		7°27′25.61”					
6	Aare-Zehndermätteli	46°58′50.91”	511	4.00/1.91	15.22	328.56	190
		7°26′20.79”					
7	Worble	46°57′30.28”	560	5.79/0.73	29.54	569.26	422
		7°30′56.50”					
8	Amletebach	46°47′04.77”	550	3.01/2.11	12.45	220.68	171
		7°34′18.86”					
9	^*^Gürbe	46°53′22.54”	522	2.67/1.94	18.53	407.29	294
		7°30′05.42”					

^a^Mean water temperature during the incubation of the eggs at natural spawning places and its standard deviation.

^b^Mean alpha diversity measure “phylogenetic distance”.

^c^“Chao 1”, and

^d^“observed number of species”^47^. ^*^At these two locations water samples had been collected simultaneously.

**Table 2:** KEGG Pathways with significant differences between bacterial communities of brown trout eggs and corresponding water samples as predicted by PICRUSt.

KEGGOrthologs	KEGG genes/enzymes	KEGG pathways	*p*-values(corrected)	Difference
K11754	[EC:6.3.2.12/6.3.2.17]	Folate biosynthesis	1.85E-02	0.13
K02032	Peptide/nickel transport system ATP-binding protein	Carbon fixation in photosynthetic organisms	3.91E-02	0.12
K01491	[EC:1.5.1.5/3.5.4.9]	One carbon pool by folate; Carbon fixation pathways in prokaryotes	3.62E-02	0.11
K00602	[EC:2.1.2.3/3.5.4.10]	Biosynthesis of antibiotics	3.79E-02	0.11
K01953	[EC:6.3.5.4]	Alanine, aspartate and glutamate metabolism	4.43E-02	0.09
K03593	ATP-binding protein involved in chromosome partitioning	Biosynthesis of amino acids	9.66E-03	0.08
K01840	[EC:5.4.2.8]	Fructose and mannose metabolism	1.88E-02	0.08
K00334	[EC:1.6.5.3]	Oxidative phosphorylation	3.40E-03	0.08
K00974	[EC:2.7.7.72/3.1.3.4]	RNA transport	4.84E-02	0.08
K03568	TldD protein	no pathway	4.33E-02	0.07
K01783	[EC:5.1.3.1]	Pentose phosphate pathway; Pentose and glucuronate interconversions	7.82E-03	0.07
K00335	[EC:1.6.5.3]	Oxidative phosphorylation	5.12E-03	0.07
K00616	[EC:2.2.1.2]	Biosynthesis of antibiotics	9.16E-03	0.07
K03555	DNA mismatch repair protein MutS	Mismatch repair	8.84E-03	0.07
K00302	[EC:1.5.3.1]	Glycine, serine and threonine metabolism	4.45E-02	0.07
K09808	Lipoprotein-releasing system permease protein	ABC transporters	6.92E-03	0.07
K11175	[EC:2.1.2.2]	Biosynthesis of antibiotics	9.37E-03	0.07
K00605	[EC:2.1.2.10]	Glycine, serine and threonine metabolism; Glyoxylate and dicarboxylate metabolism	3.83E-05	0.07
K00332	[EC:1.6.5.3]	Oxidative phosphorylation	2.85E-02	0.06
K01745	[EC:4.3.1.3]	Histidine metabolism	4.67E-02	0.06
K01572	[EC:4.1.1.3]	Pyruvate metabolism	4.26E-02	0.06
K00303	[EC:1.5.3.1]	Glycine, serine and threonine metabolism	3.88E-02	0.06
K01893	[EC:6.1.1.22]	Aminoacyl-tRNA biosynthesis	6.77E-04	0.06
K00339	[EC:1.6.5.3]	Oxidative phosphorylation	8.14E-03	0.06
K01560	[EC:3.8.1.2]	Microbial metabolism in diverse environments; Phlorocyclohexane and chlorobenzene degradation; Chloroalkane and chloroalkene degradation	4.88E-02	0.06
K08978	Bacterial/archaeal transporter family protein	Biosynthesis of antibiotics	3.75E-02	0.06
K04567	[EC:6.1.1.6]	Aminoacyl-tRNA biosynthesis	1.60E-02	0.06
K11717	[EC:2.8.1.7 4.4.1.16]	Selenocompound metabolism	9.53E-03	0.06

PICRUSt v.1.0.0^52^ was used to predict the functional potential of bacteria found on naturally spawned brown trout eggs at the late-eyed developmental stage compared to two corresponding water samples. This table contains all the predicted KEGG pathways (at the third hierarchical level) that varied significantly (*p*-value <0.05 with Benjamini – Hochberg multiple testing correction controlling the FDR; comparisons were done in STAMP v.2.1.2^53^) between trout eggs and water samples and that showed a mean difference between means of >5%. A complete list of 69 significantly different pathways is shown in the Supplemental Information (Fig. S6, Table S5).
