# Supplementary material for: Increased diversity of egg-associated bacteria on brown trout (Salmo trutta) at elevated temperatures
Source: Sci Rep. 2015 Nov 27;5:17084. doi: 10.1038/srep17084 (PMC4661462; doi:10.1038/srep17084)
Supplement: Supplementary Information [file srep17084-s1.doc]

**Supplemental Information**

**Increased diversity of egg-associated bacteria on brown trout (*Salmo trutta*) at elevated temperatures**

Laetitia G.E. Wilkins^1,*^, Aude Rogivue^1,2^, Frédéric Schütz^3,4^, Luca Fumagalli^1,§^, and Claus Wedekind^1,§^

^1^Department of Ecology and Evolution, Biophore, University of Lausanne, 1015 Lausanne, Switzerland

^2^WSL Swiss Federal Research Institute, Zürcherstrasse 111, 8903 Birmensdorf, Switzerland

^3^Center for Integrative Genomics, Génopode, University of Lausanne, 1015 Lausanne, Switzerland

^4^SIB Swiss Institute of Bioinformatics, Génopode, 1015 Lausanne, Switzerland

^§^Joint senior authors

^*^Correspondence and requests for materials should be addressed to L. Wilkins, Tel: +41 21 692 42 19; Fax: +41 21 692 42 65; email: [laetitia.wilkins@unil.ch](mailto:claus.wedekind@unil.ch)

**Inventory of Supplemental Information**

**Supplemental Methods**

**Supplemental Results**

**Table S1** Number of bacterial sequence reads before and after quality control

**Table S2** Bacterial taxa found in the negative control

**Table S3** Bacterial sequences on brown trout eggs found in this study

**Table S4** Spawning locations and unfiltered alpha diversity measures of bacterial communities on naturally spawned brown trout eggs

**Table S5** KEGG legend

**Table S6** Distance matrix showing pairwise distances between all spawning locations

**Table S7** Bootstrapping approach showing the effect of sample size within groups on non-parametric analysis of variance estimates

**Table S8** Multiple regression analysis testing the association of average water temperatures of five equal time periods from spawning until sampling and alpha diversities of bacterial communities on brown trout eggs

**Figure S1** Relative composition of main bacterial families on brown trout eggs at different spawning places and in corresponding water samples

**Figure S2** Heatmaps of the most abundant 120 bacterial taxa on brown trout eggs at different spawning places and in corresponding water samples

**Figure S3** Rarefaction curves for filtered alpha diversity measures of bacterial communities on brown trout eggs and in corresponding water samples

**Figure S4** Rarefaction curves for unfiltered alpha diversity measures of bacterial communities on brown trout eggs and in corresponding water samples

**Figure S5** Number of bacterial taxa that could be found at different fractions of samples

**Figure S6** PICRUSt metagenome prediction predicted higher abundance of genes in 69 pathways of brown trout egg-associated bacterial communities relative to corresponding water samples

**Figure S7** Spatial pattern of unfiltered bacterial community composition on brown trout eggs in the Aare system

**Figure S8** Two-dimensional PCoA plot of observed and simulated, unweighted UniFrac distances of brown trout egg-associated bacteria between the river Aare and a spawning place in the river Inn

**Figure S9** Three-dimensional PCoA plot of unfiltered, unweighted UniFrac distances between bacterial communities on brown trout eggs and in water samples

**Figure S10** Relationship of water temperature and unfiltered bacterial alpha diversities on brown trout eggs at natural spawning places

**Supplemental References**

Supplemental Methods

*Detailed sample acquisition*

Naturally spawned brown trout eggs were collected from five different locations within the river Aare (belonging to the Rhine basin that feeds the North Sea), three locations in tributaries of the river Aare (Amletebach, Gürbe, and Worble), and one location in the river Inn (belonging to the Danube Basin that feeds the Black sea; Fig. 1, Table 1, main manuscript). A rake was used to open the riverbed and eggs were collected by hand with the help of a sieve. To minimize the effects of contamination, sieves were rinsed with bleach and alcohol between different spawning locations, and sterile gloves were worn. After collection, eggs were immediately rinsed with 2 L of autoclaved and filtered water (0.2 µm, Millipore, Zug, Switzerland). Embryo developmental stage was determined on a glass slide under a field light microscope (Motic Microscopes 1820, Wetzlar, Germany). Eggs were then frozen in liquid nitrogen and later stored at -80°C without a storage buffer. Only fertilized eggs at the late-eyed stage were used for further analysis (developmental stage 6 in Luczynski and Kirklewska^1^; sampled between 12.02.-02.03.2010, Table 1, main manuscript). Eight eggs per spawning location were sampled for individual DNA extractions. At two spawning locations (Gürbe and Lerbestrasse) 1.5 L water was collected in a sterile glass bottle as close as possible to the location where eggs had been found. This water was filtered (0.2 µm, Millipore, Zug, Switzerland), and the filters were stored at -80°C (without storage buffer) until bacterial DNA extraction. Temperature loggers (HOBO Water Temperature Pro v2 Data Logger – U22-001, Bakrona Zürich) were installed at every location around the beginning of the spawning season. They were retrieved by the end of the spawning season. Temperature was measured continuously at intervals of 10 minutes.

*Molecular genetic analyses*

Each individual egg was homogenized in a 2 mL Eppendorf tube (Sarstedt, Nümbrecht, Germany) in 1.6 mL of buffer ASL (Qiagen, Hombrechtikon, Switzerland) with a mixer mill (MM300; Retsch, Düsseldorf, Germany) for 2 x 30 seconds using six tungsten beads (3 mm, Qiagen), five silica beads (1.5 mm, Qiagen), and 0.4 g silica powder (0.2 mm, Qiagen). The homogenate was heated to 95°C for 5 minutes, then vortexed again for 15 seconds and centrifuged at 13,000 rpm for 1 minute. An InhibitEX® tablet (Qiagen) was added to each supernatant (vortexed until the tablet was completely suspended) to digest DNA extraction inhibitors (lipids and proteins from the egg), then the sample was centrifuged again at 13,000 rpm for 3 minutes. The new supernatant was treated with 25 µL proteinase K (Qiagen) per sample and incubated at 70°C for 10 minutes. This mix was purified with absolute ethanol and subjected to the QIAamp DNA micro kit (Qiagen) following the manufacturer’s protocol. DNA was eluted in 10 µL of DNase-free water (Milipore, Zug, Switzerland). The same protocol was used for water filters, with the extra step of adding carrier RNA (supplied in the kit).

PCR was performed with a bacterial-specific primer pair, 27F and 338R^2,3^ that amplifies a 311-bp fragment of the V1-V2 hypervariable region of bacterial 16S rRNA. This primer pair has widely been used in metagenomic studies and was therefore chosen to make our study comparable to other microbial characterizations of different habitats^2,4^. The 338R primer included a unique 10-bp long sequence tag to barcode each spawning location (Table S1). The barcodes had been tested *in silico* and empirically to show that they do not bias amplification^2,3,5^. Eight individual bacterial DNA extractions were pooled for every spawning location resulting in a total of nine PCR reactions representing the nine different spawning locations, two PCR reactions for water samples, and one PCR reaction for the negative control that consisted of all reagents except the extracted DNA. Each PCR was performed in a total volume of 25 µL consisting of 1-50 ng bacterial genomic DNA, 2.5 µL of 10X PCR buffer, 400 µM of each dNTP, 2.5 mM of MgCl_2_, 0.6 µM of each primer, and 0.625U of Taq polymerase (Life Technologies, Zug, Switzerland). The thermal profile was modified from Berry *et al.* (2011)^5^ and consisted of a two-step PCR. Step I with primer pair 27F and 338R (without tag): 3 minutes at 94°C, 25 cycles of 94°C for 30 seconds, 56°C for 30 seconds, 72°C for 30 seconds, and a final extension at 72°C for 10 minutes. Step II: 1 µL of the product of step I, 2.5 µL of 10X PCR buffer, 400 µM of each dNTP, 2.5 mM of MgCl_2_, 0.6 µM individually MID-tagged fusion primers 27F and individually tagged 338R (Table S1), and 0.625U of Taq polymerase (Life Technologies) in a total volume of 50 µL (1:50 dilution). The following conditions were used for step II: 3 minutes at 94^o^ C, 5 cycles of 94°C for 30 seconds, 56°C for 30 seconds, 72°C for 30 seconds, and a final extension at 72°C for 10 minutes. Amplified products were purified with the Wizard® SV Gel and PCR Clean-Up System (Promega, Dübendorf, Switzerland). For every sample five PCR reactions were pooled from PCR step I into PCR step II to avoid amplification bias^5^. Cleaned PCR products were run on an agarose gel (1.5%, 100 V, 45 minutes), cut out, purified, and quantified using a Qubit ® 2.0 Fluorometer (Life Technologies) and on a bioanalyzer using the DNA 1000 kit (Agilent, Morges, Switzerland). PCR reactions of 15 ng/µL DNA were pooled in equimolar amounts into a 10 mM Tris-HCl buffer at pH 8.5 and sent for 454 pyrosequencing (Microsynth, Balgach, Switzerland) on a Genome Sequencer FLX System (Roche, Basel, Switzerland).

*Quality control*

All steps of the 454 pyrosequencing data quality control were done in the QIIME framework v.1.6.0^6^. Raw reads were split based on their 10-bp long barcode. Forward and reverse primers were removed, and sequences produced by sequencing errors were filtered out^7^. To pass, a sequence read needed to: (i) have a perfect match to the barcode and the 16S rRNA primer, (ii) be at least 300-bp long, (iii) have no more than two undetermined bases, and (iv) have at least a 60% match to a 16S rRNA gene sequence from the Greengenes database^8,9^. The resulting clean reads were screened for chimeras using the UCHIME algorithm^10^. As a last step of quality control, all sequences were removed that had been detected in the blank control sample of only beads and buffers. All resulting reads (Table S3) were used for further analysis.

*Corrected geographic distances between spawning places*

Geographic distances between spawning places might not be an accurate distance estimate due to the variable water flow in a river system^11^. The impact of water flow in the river is strong enough that it affects the energy investment of a migrating fish^12^. A simple estimation of flow rates and the resulting energy a fish has to invest into swimming against the stream is very difficult^11,13,14^. A common approach to take water flow into account is to correct for the slope between two distances using altitude differences^15-17^. Accordingly, all the geographic distances against the stream were increased by altitude differences. The resulting distance is the hypotenuse according to Pythagorean theorem with the altitude difference and the geographic distance as the perpendicular legs to obtain a measure of the slope of the stream*.* The relationship of resulting asymmetric distance matrices and pairwise, unweighted UniFrac bacterial community distances between different spawning places in the river Aare system were investigated using correspondence analysis with the ‘ape’ package in R^18,19^. A coefficient of concordance between the two asymmetric distance matrices was calculated through 99’999 permutations. To estimate *p*-values an *a posteriori* permutation test of the contributions of individual distances was carried out. Potential migration barriers were not included in this analysis.

*Non-parametric analyses of variance*

Adonis is a nonparametric statistical method that can be used to compare bacterial communities^20^. It takes into account a categorical variable that groups bacterial communities and calculates the percentage of variation in unweighted UniFrac distances (R^2^) that is explained by the supplied categorical variable. It creates a set by first identifying the relevant centroids of each group and then calculating the squared deviations from these points. Significance tests are performed using *F*-tests based on sequential sums of squares from permutations (n = 99,999) of all bacterial communities irrespective of the grouping. ANOSIM is another nonparametric statistical method that tests whether two or more groups of bacterial communities are significantly different from each other. It differs from adonis in how its R^2^s are calculated^6^.

*Bootstrapping of bacterial distributions*

Applying the statistical procedure of re-sampling with replacement, we tested if the bacterial community at a spawning location in the river Inn was different from the bacterial communities at four spawning places in the main river Aare system. Bootstrapping was done in R and worked as follows: One thousand individual bacterial communities were simulated for the river Inn spawning location in order to obtain a density of its bacterial community composition and corresponding confidence intervals. To simulate a bacterial community at a spawning location in the river Inn, bacterial sequences were randomly sampled with replacement from the bacterial communities of four river Aare spawning locations (locations 3-6, Fig. 1, main manuscript). The same number of sequences that had been found in the true river Inn location was sampled. This was done 1,000 times. The observed, unweighted UniFrac distance between the observed river Inn bacterial community and the bacterial communities of locations 3-6 was then compared to the simulated mean unweighted UniFrac distances between the simulated Inn bacterial community and the Aare bacterial communities. Finally, the probability of obtaining the observed, unweighted UniFrac distance between the two groups in the simulations was calculated. The same analysis was applied to compare bacterial communities of water samples to their corresponding bacterial communities on brown trout egg samples.

We also applied a bootstrapping approach to infer the effect of sample size within groups on the comparison of bacterial communities on brown trout eggs in the main river Aare (n = 4) with its tributaries (n = 3). Using sampling with replacement from the distribution of bacterial sequences in the two respective groups, we increased the number of samples within groups sequentially. Then we tested with adonis whether the two groups turn out significantly different with regard to their bacterial communities. For each increase in sample size 1,000 adonis tests were run. Sample size, *p*-values and power estimates are shown in table S7.

Supplemental Results

*Analyses utilizing unfiltered bacterial community compositions*

In order to investigate the effect of our quality control including the removal of all bacterial taxa that had been found in the negative control of only beads and buffers, we also tested for a significant correlation of alpha diversity measures of brown trout egg-associated bacterial communities including all bacterial taxa and average water temperature at the spawning location during the whole incubation time. All three unfiltered alpha diversity measures of brown trout egg-associated bacterial communities significantly increased with the average water temperature: Chao 1: *τ* = 0.59, *z* = 1.92, *p* = 0.03; observed number of species: *τ* = 0.54, *z* = 1.68, *p* = 0.04; and phylogenetic distance: *τ* = 0.68, *z* = 2.49, *p* = 0.02 (Fig. S10). Unfiltered bacterial community distances (unweighted UniFrac) were significantly correlated with variations in water temperature while controlling for geographic distances among spawning locations within the Aare river system (*r* = 0.44, *p* = 0.001). All unfiltered alpha diversity estimates are also shown in Table S4.

Geographic distances among spawning locations within the Aare system were not correlated with unfiltered phylogenetic distances of bacterial communities (unweighted UniFrac) on brown trout eggs using neither Mantel statistics (*r* = -0.32, *p* = 0.15) nor partial Mantel statistics while controlling for variation in water temperature (*r* =- 0.21, *p* = 0.89). No sign of isolation-by-distance could be detected. Corrected geographic distances and unfiltered, unweighted UniFrac distances of bacterial communities did not result in any significant improvement of the spatial relationship (*r* = -0.18, *p* = 0.11; Fig. S7a). Correspondence analysis of genetic differentiation among different brown trout populations (*D_est_*) and unfiltered, unweighted UniFrac bacterial community distances did not result in a significant association (*r* = -0.06, *p* = 0.69; Fig. S7b). All distance matrices are listed in Table S6.

Table S1: Number of bacterial sequence reads before and after quality control.

The first column corresponds to spawning locations in Fig. 1 and Table 1 (in the main manuscript). The second column shows the number of reads after 454 pyrosequencing before quality control. “Denoising” gives the number of reads after exclusion of PCR-artifacts and chimera, while “Bead-Filtering” gives the remaining number of reads after filtering out all sequences, that had been found in the negative control sample. The number of different operational taxonomic units of bacteria (OTUs) is shown, which could be assigned with the RDP classifier 2.2^21^ and the Greengenes reference database version 12.10^8^. Individual barcodes were used to pool samples for 454 pyrosequencing.

| **Sample-ID** | **Reads (454)** | **Denoising** | **Bead-Filtering** | **Number of OTUs** | **Barcodes used** |
| --- | --- | --- | --- | --- | --- |
| 1 | 4970 | 4616 | 4584 | 91 | ATCAGACACG |
| 2 | 5150 | 4736 | 4708 | 205 | ACGAGTGCGT |
| 3 | 4844 | 4486 | 4435 | 340 | CTCGCGTGTC |
| 4 | 6287 | 5851 | 5825 | 133 | TAGTATCAGC |
| 5 | 5030 | 4624 | 4572 | 187 | CGTGTCTCTA |
| 6 | 1361 | 1224 | 1195 | 190 | ATATCGCGAG |
| 7 | 4466 | 4163 | 4092 | 422 | AGACGCACTC |
| 8 | 3358 | 2964 | 2948 | 171 | ACGCTCGACA |
| 9 | 6215 | 5761 | 5690 | 294 | AGCACTGTAG |
| 10^a^ | 5260 | 4837 | 0(16) | 0 | ACGACTACAG |
| 11^b^ | 6748 | 6219 | 5952 | 342 | ACTACTATGT |
| 12^c^ | 3372 | 3112 | 3013 | 343 | ACGCGAGTAT |

^a^Negative control sample: 78 different OTUs had been found in the negative control, they were filtered out in all other samples. ^b^Water sample at Gürbe (location 9), and ^c^water sample at Aare-Lerbestrasse (location 5).

Table S2: Bacterial taxa found in the negative control.

| **Sequences** | **Consensus lineage** | **Level^a^** | **Habitat^b^** |
| --- | --- | --- | --- |
| 602 | *Lactococcus* |  | Milk^22^ |
| 539 | Leuconostocaceae | F |  |
| 482 | *Leuconostoc* |  | Milk^22^ |
| 420 | *Lactococcus* |  | Milk^22^ |
| 401 | *Lactococcus* |  | Milk^22^ |
| 340 | *Citrobacter* |  | Generalist^22^ |
| 127 | *Acinetobacter johnsonii* |  | Soil, Freshwater^23^ |
| 119 | *Chryseobacterium* |  | Milk, cold-tolerant^24, 25^ |
| 119 | *Acinetobacter* |  | Soil^22, 26^ |
| 86 | Aeromonadaceae | F |  |
| 81 | *Arcobacter* |  | Generalist^22^ |
| 77 | *Veillonella* |  | Oral mucosa of humans^22, 27^ |
| 73 | Gammaproteobacteria | C |  |
| 71 | *Staphylococcus epidermidis* |  | Human skin & mucus^28, 29^ |
| 66 | *Lactococcus* |  | Milk^22^ |
| 63 | *Acidovorax* |  | Crops^30^ |
| 58 | *Lactococcus* |  | Milk^22^ |
| 53 | Enterobacteriaceae | F |  |
| 52 | TM7-3 | NA |  |
| 51 | *Enhydrobacter* |  | Freshwater^31, 32^ |
| 41 | *Enterococcus faecalis* |  | Intestines^33^ |
| 41 | *Microvirgula aerodenitrificans* |  | Sludge^34^ |
| 41 | *Acinetobacter* |  | Soil^22^ |
| 34 | *Acinetobacter* |  | Soil^22^ |
| 33 | *Acinetobacter* |  | Soil^22^ |
| 29 | *Propionibacterium acnes* |  | Human skin^35^ |
| 28 | Betaproteobacteria | C |  |
| 27 | *Flavobacterium* |  | Potential fish pathogen^36^ |
| 22 | *Acinetobacter* |  | Soil^22^ |
| 20 | Streptococcaceae | F |  |
| 20 | *Lactococcus* |  | Milk^22^ |
| 17 | Streptococcaceae | F |  |
| 17 | *Comamonas* |  | Generalist^37^ |
| 17 | *Comamonas* |  | Generalist^37^ |
| 15 | *Veillonella dispar* |  | Oral mucosa of humans^38^ |
| 13 | Propionibacteriacea | F |  |
| 12 | *Chryseobacterium* |  | Milk, cold-tolerant^39^ |
| 12 | *Comamonas* |  | Generalist^37^ |
| 12 | *Vogesella* |  | Freshwater^40^ |
| 12 | *Acinetobacter* |  | Soil^22^ |
| 11 | Actinomycetales | O |  |
| 11 | *Lactobacillus delbrueckii* |  | Milk^41^ |
| 10 | *Corynebacterium* |  | Saprophyt^22^ |
| 10 | Propionibacteriacea | F |  |
| 10 | *Lactococcus* |  | Milk^22^ |
| 9 | *Novosphingobium* |  | Estuarines^42^ |
| 8 | Actinomycetales | O |  |
| 8 | Flavobacteriaceae | F |  |
| 8 | Neisseriaceae | F |  |
| 6 | Actinomycetaceae | F |  |
| 6 | *Actinomyces* |  | Commensal in endotherms^22, 43^ |
| 6 | Comamonadaceae | F |  |
| 6 | Comamonadaceae | F |  |
| 5 | *Lactococcus* |  | Milk^22^ |
| 5 | *Lactococcus* |  | Milk^22^ |
| 5 | *Lactococcus* |  | Milk^22^ |
| 5 | *Enhydrobacter* |  | Freshwater^31, 32^ |
| 4 | *Chryseobacterium* |  | Milk, cold-tolerant^24, 25^ |
| 4 | Streptophyta | O |  |
| 4 | Leuconostocaceae | F |  |
| 4 | Rhodospirillaceae | F |  |
| 4 | *Comamonas* |  | Generalist^37^ |
| 3 | Streptococcaceae | F |  |
| 2 | *Flavobacterium succinicans* |  | Potential fish pathogen^36^ |
| 2 | *Streptococcus pseudopneumoniae* |  | Human lungs^44^ |
| 2 | Rickettsiales | O |  |
| 1 | Acidimicrobiales | O |  |
| 1 | *Flavobacterium* |  | Potential fish pathogen^36^ |
| 1 | *Prochlorococcus marina* |  | Marine, very abundant^45^ |
| 1 | Rhizobiales | O |  |
| 1 | Rhodobacteraceae | F |  |
| 1 | *Sphingobium* |  | Soil^46, 47^ |
| 1 | *Limnohabitans* |  | Freshwater^48^ |
| 1 | *Limnohabitans* |  | Freshwater^48^ |
| 1 | *Janthinobacterium* |  | Soil^49^ |
| 1 | *Janthinobacterium lividum* |  | Soil^50^ |
| 1 | Aeromonadaceae | F |  |
| 1 | Enterobacteriaceae | F |  |

Seventy-eight different consensus lineages could be found in the negative control with only beads and buffers. They are either linked to contaminations or to systematic errors produced by the pyrosequencing technique. The table gives the number of reads for each bacterial taxon that were filtered out from the remaining samples ‘Sequences’, as well as the names for the consensus lineages of the sequences found based on an open blast search against the RDP database ‘Consensus lineage’. ^a^For sequences that could not be described down to the genus or species origin, the level of description is given (‘C’ = class, ‘F’ = family, and ‘O’ = order). ^b^Habitat type of a consensus lineage was derived from a search in Web Of Knowledge^TM^ v.5.12 (Thomson Reuters) or according to Austin & Austin (2007). Bacterial taxa that are typically found on humans are shaded in grey.

Table S3: Bacterial sequences on brown trout eggs found in this study.

Names are shown for bacterial consensus lineages of the sequences found in this study after the removal of all sequences that had been found in the negative control of only beads and buffers. Bacterial names were inferred by comparing individual sequences with an online database (RDP classifier 2.2^21^ and Greengenes reference database version 12.10^8^). Multiples of the same names represent different consensus sequences matching with at least 97% sequence similarity to the same reference in the RDP database (due to *de novo* OTU picking algorithm and open reference blast search, see main manuscript). Counts represent how many sequences were found matching a specific reference. Only matches with more than 3 reads are shown in this table.

| **Counts** | **Consensus lineages** | |  |  |  |  |
| --- | --- | --- | --- | --- | --- | --- |
| 4 | Gemmatimonadetes** | Gemmatimonadetes | Gemmatimonadales | A1-B1 |  |  |
| 4 | Proteobacteria | Alphaproteobacteria | Rhodospirillales | Acetobacteraceae | Roseomonas |  |
| 4 | Actinobacteria | Actinobacteria | Actinomycetales | Actinomycetaceae | |  |
| 4 | Proteobacteria* | Deltaproteobacteria | Bdellovibrionales | Bacteriovoracaceae | |  |
| 4 | Bacteroidetes | Sphingobacteriia | Sphingobacteriales | Chitinophagaceae |  |  |
| 4 | Bacteroidetes | Sphingobacteriia | Sphingobacteriales | Chitinophagaceae |  |  |
| 4 | Proteobacteria | Betaproteobacteria | Burkholderiales | Comamonadaceae |  |  |
| 4 | Proteobacteria | Betaproteobacteria | Burkholderiales | Comamonadaceae | Hydrogenophaga |  |
| 4 | Proteobacteria | Betaproteobacteria | Burkholderiales | Comamonadaceae | Hylemonella |  |
| 4 | Proteobacteria* | Betaproteobacteria | Burkholderiales | Comamonadaceae | Variovorax | paradoxus |
| 4 | Thermi** | Deinococci | Deinococcales | Deinococcaceae | Deinococcus |  |
| 4 | Proteobacteria | Gammaproteobacteria | Enterobacteriales | Enterobacteriaceae | Klebsiella |  |
| 4 | Firmicutes | Bacilli | Lactobacillales | Enterococcaceae | Vagococcus |  |
| 4 | Firmicutes | Erysipelotrichi | Erysipelotrichales | Erysipelotrichaceae | Erysipelothrix |  |
| 4 | Bacteroidetes | Flavobacteriia | Flavobacteriales | Flavobacteriaceae | Flavobacterium |  |
| 4 | Bacteroidetes | Flavobacteriia | Flavobacteriales | Flavobacteriaceae | Flavobacterium | gelidilacus |
| 4 | Bacteroidetes | Flavobacteriia | Flavobacteriales | Flavobacteriaceae | Flavobacterium |  |
| 4 | Bacteroidetes* | Sphingobacteriia | Sphingobacteriales | Flexibacteraceae | Runella |  |
| 4 | Bacteroidetes* | Sphingobacteriia | Sphingobacteriales | Flexibacteraceae | Dyadobacter |  |
| 4 | Bacteroidetes* | Sphingobacteriia | Sphingobacteriales | Flexibacteraceae |  |  |
| 4 | Bacteroidetes* | Sphingobacteriia | Sphingobacteriales | Flexibacteraceae |  |  |
| 4 | Firmicutes* | Bacilli | Gemellales | Gemellaceae |  |  |
| 4 | Proteobacteria** | Deltaproteobacteria | Myxococcales | Haliangiaceae |  |  |
| 4 | Proteobacteria | Alphaproteobacteria | Rhizobiales | Hyphomicrobiaceae | |  |
| 4 | Proteobacteria | Alphaproteobacteria | Rhizobiales | Hyphomicrobiaceae | |  |
| 4 | Firmicutes | Clostridia | Clostridiales | Lachnospiraceae |  |  |
| 4 | Acidobacteria* | Acidobacteria-6 | iii1-15 | mb2424 |  |  |
| 4 | Proteobacteria | Betaproteobacteria | Methylophilales | Methylophilaceae | Methylotenera | mobilis |
| 4 | Proteobacteria | Betaproteobacteria | Methylophilales | Methylophilaceae | Methylotenera |  |
| 4 | Actinobacteria** | Actinobacteria | Actinomycetales | Microbacteriaceae | Leucobacter |  |
| 4 | Proteobacteria | Betaproteobacteria | Neisseriales | Neisseriaceae | Deefgea |  |
| 4 | Proteobacteria | Alphaproteobacteria | Rhodobacterales | Rhodobacteraceae | Rhodobacter |  |
| 4 | Proteobacteria | Alphaproteobacteria | Rhodobacterales | Rhodobacteraceae |  |  |
| 4 | Proteobacteria | Alphaproteobacteria | Rhodobacterales | Rhodobacteraceae |  |  |
| 4 | Proteobacteria | Betaproteobacteria | Rhodocyclales | Rhodocyclaceae |  |  |
| 4 | TM7 | TM7-3 | I025 | Rs-045 |  |  |
| 4 | Bacteroidetes | Sphingobacteriia | Sphingobacteriales | Saprospiraceae |  |  |
| 4 | Bacteroidetes | Sphingobacteriia | Sphingobacteriales | Saprospiraceae |  |  |
| 4 | Bacteroidetes* | Sphingobacteriia | Sphingobacteriales | Saprospiraceae |  |  |
| 4 | Bacteroidetes** | Sphingobacteriia | Sphingobacteriales | Sphingobacteriaceae | Pedobacter |  |
| 4 | Proteobacteria* | Gammaproteobacteria | Xanthomonadales | Xanthomonadaceae | |  |
| 4 | Proteobacteria** | Betaproteobacteria | SC-I-84 |  |  |  |
| 4 | Acidobacteria | Chloracidobacteria | |  |  |  |
| 4 | Cyanobacteria | Chloroplast | Streptophyta |  |  |  |
| 4 | GN02 | BD1-5 |  |  |  |  |
| 4 | Proteobacteria | Alphaproteobacteria | Rhizobiales |  |  |  |
| 4 | Bacteroidetes | Sphingobacteriia | Sphingobacteriales | |  |  |
| 4 | Proteobacteria* | Alphaproteobacteria | |  |  |  |
| 4 | Proteobacteria* | Betaproteobacteria | Burkholderiales |  |  |  |
| 4 | OD1* | ZB2 |  |  |  |  |
| 4 | Bacteroidetes* | Sphingobacteriia | Sphingobacteriales | |  |  |
| 5 | Proteobacteria | Deltaproteobacteria | Bdellovibrionales | Bacteriovoracaceae | |  |
| 5 | Actinobacteria | Acidimicrobiia | Acidimicrobiales | C111 |  |  |
| 5 | Proteobacteria** | Betaproteobacteria | Burkholderiales | Comamonadaceae | Comamonas |  |
| 5 | Proteobacteria | Betaproteobacteria | Burkholderiales | Comamonadaceae |  |  |
| 5 | Proteobacteria | Betaproteobacteria | Burkholderiales | Comamonadaceae | Rubrivivax |  |
| 5 | Bacteroidetes* | Sphingobacteriia | Sphingobacteriales | Flammeovirgaceae | A4 |  |
| 5 | Bacteroidetes** | Flavobacteriia | Flavobacteriales | Flavobacteriaceae | Flavobacterium |  |
| 5 | Bacteroidetes | Flavobacteriia | Flavobacteriales | Flavobacteriaceae | Flavobacterium |  |
| 5 | Bacteroidetes | Flavobacteriia | Flavobacteriales | Flavobacteriaceae | Flavobacterium |  |
| 5 | Bacteroidetes | Flavobacteriia | Flavobacteriales | Flavobacteriaceae |  |  |
| 5 | Proteobacteria | Deltaproteobacteria | Myxococcales | Haliangiaceae |  |  |
| 5 | Proteobacteria | Alphaproteobacteria | Rhizobiales | Hyphomicrobiaceae | |  |
| 5 | Actinobacteria* | Actinobacteria | Actinomycetales | Nakamurellaceae |  |  |
| 5 | Proteobacteria | Betaproteobacteria | Nitrosomonadales | Nitrosomonadaceae | |  |
| 5 | Proteobacteria | Alphaproteobacteria | Rhizobiales | Rhizobiaceae |  |  |
| 5 | Proteobacteria** | Alphaproteobacteria | Rhodobacterales | Rhodobacteraceae |  |  |
| 5 | Proteobacteria | Alphaproteobacteria | Rhodobacterales | Rhodobacteraceae | Rhodobacter |  |
| 5 | Proteobacteria | Alphaproteobacteria | Rhodobacterales | Rhodobacteraceae | Rhodobacter |  |
| 5 | Proteobacteria | Alphaproteobacteria | Rhodobacterales | Rhodobacteraceae |  |  |
| 5 | Bacteroidetes | Sphingobacteriia | Sphingobacteriales | Saprospiraceae |  |  |
| 5 | Acidobacteria* | Solibacteres | Solibacterales | Solibacteraceae | Candidatus | solibacter |
| 5 | Bacteroidetes** | Sphingobacteriia | Sphingobacteriales | Sphingobacteriaceae | Sphingobacterium | |
| 5 | Bacteroidetes | Sphingobacteriia | Sphingobacteriales | Sphingobacteriaceae | Pedobacter |  |
| 5 | Proteobacteria | Betaproteobacteria | Ellin6067 |  |  |  |
| 5 | Proteobacteria | Deltaproteobacteria | Myxococcales |  |  |  |
| 5 | Bacteroidetes | Flavobacteriia |  |  |  |  |
| 5 | Bacteroidetes* | Sphingobacteriia | Sphingobacteriales | |  |  |
| 5 | Proteobacteria* | Alphaproteobacteria | BD7-3 |  |  |  |
| 6 | Actinobacteria | Actinobacteria | Actinomycetales | Actinomycetaceae | Actinomyces |  |
| 6 | Proteobacteria | Betaproteobacteria | Burkholderiales | Comamonadaceae | Limnohabitans |  |
| 6 | Proteobacteria | Gammaproteobacteria | Enterobacteriales | Enterobacteriaceae | Morganella |  |
| 6 | Bacteroidetes* | Flavobacteriia | Flavobacteriales | Flavobacteriaceae | Flavobacterium |  |
| 6 | Bacteroidetes | Sphingobacteriia | Sphingobacteriales | Flexibacteraceae | Emticicia |  |
| 6 | Bacteroidetes | Sphingobacteriia | Sphingobacteriales | Flexibacteraceae |  |  |
| 6 | Proteobacteria | Alphaproteobacteria | Rhizobiales | Hyphomicrobiaceae | Devosia |  |
| 6 | Firmicutes | Bacilli | Lactobacillales | Lactobacillaceae | Lactobacillus | delbrueckii |
| 6 | Actinobacteria | Actinobacteria | Actinomycetales | Micrococcaceae | Rothia | mucilaginosa |
| 6 | Proteobacteria | Gammaproteobacteria | Pseudomonadales | Moraxellaceae | Acinetobacter |  |
| 6 | Proteobacteria** | Betaproteobacteria | Neisseriales | Neisseriaceae | Aquitalea | magnusonii |
| 6 | Proteobacteria | Betaproteobacteria | Burkholderiales | Oxalobacteraceae |  |  |
| 6 | Bacteroidetes* | Bacteroidia | Bacteroidales | Prevotellaceae | Prevotella |  |
| 6 | Proteobacteria | Alphaproteobacteria | Rhodobacterales | Rhodobacteraceae | Rhodobacter |  |
| 6 | Proteobacteria | Alphaproteobacteria | Sphingomonadales | Sphingomonadaceae | Novosphingobium | |
| 6 | Firmicutes* | Bacilli | Bacillales | Staphylococcaceae | Staphylococcus |  |
| 6 | Firmicutes | Bacilli | Lactobacillales | Streptococcaceae |  |  |
| 6 | GN02 | BD1-5 |  |  |  |  |
| 6 | OD1* | ZB2 |  |  |  |  |
| 7 | Proteobacteria** | Alphaproteobacteria | Rhizobiales | Brucellaceae | Pseudochrobactrum | |
| 7 | Proteobacteria* | Betaproteobacteria | Burkholderiales | Comamonadaceae |  |  |
| 7 | Bacteroidetes | Flavobacteriia | Flavobacteriales | Flavobacteriaceae | Flavobacterium |  |
| 7 | Bacteroidetes* | Flavobacteriia | Flavobacteriales | Flavobacteriaceae | Flavobacterium |  |
| 7 | Bacteroidetes | Sphingobacteriia | Sphingobacteriales | Flexibacteraceae |  |  |
| 7 | Bacteroidetes* | Sphingobacteriia | Sphingobacteriales | Flexibacteraceae | Runella |  |
| 7 | Proteobacteria* | Alphaproteobacteria | Rhizobiales | Hyphomicrobiaceae | |  |
| 7 | Proteobacteria* | Alphaproteobacteria | Rhodobacterales | Rhodobacteraceae |  |  |
| 7 | Proteobacteria** | Betaproteobacteria | Rhodocyclales | Rhodocyclaceae |  |  |
| 7 | Bacteroidetes | Sphingobacteriia | Sphingobacteriales | Saprospiraceae |  |  |
| 7 | Bacteroidetes | Sphingobacteriia | Sphingobacteriales | Saprospiraceae |  |  |
| 7 | Bacteroidetes** | Sphingobacteriia | Sphingobacteriales | Sphingobacteriaceae | Pedobacter |  |
| 7 | Bacteroidetes | Sphingobacteriia | Sphingobacteriales | Sphingobacteriaceae | Pedobacter |  |
| 7 | Proteobacteria | Alphaproteobacteria | Sphingomonadales | Sphingomonadaceae | Novosphingobium | |
| 7 | Proteobacteria | Alphaproteobacteria | Sphingomonadales | Sphingomonadaceae | Novosphingobium | |
| 7 | Proteobacteria | Alphaproteobacteria | Sphingomonadales | Sphingomonadaceae | Novosphingobium | |
| 7 | Proteobacteria | Deltaproteobacteria | Myxococcales |  |  |  |
| 7 | Acidobacteria | Chloracidobacteria | |  |  |  |
| 7 | Acidobacteria | Chloracidobacteria | |  |  |  |
| 7 | Proteobacteria | Betaproteobacteria | YCC11 |  |  |  |
| 7 | Actinobacteria | Actinobacteria | Actinomycetales |  |  |  |
| 7 | Bacteroidetes | Sphingobacteriia | Sphingobacteriales | |  |  |
| 8 | Actinobacteria | Actinobacteria | Actinomycetales | Actinomycetaceae | |  |
| 8 | Proteobacteria | Betaproteobacteria | Burkholderiales | Comamonadaceae |  |  |
| 8 | Thermi* | Deinococci | Deinococcales | Deinococcaceae | Deinococcus |  |
| 8 | Bacteroidetes | Flavobacteriia | Flavobacteriales | Flavobacteriaceae | Flavobacterium |  |
| 8 | Bacteroidetes | Flavobacteriia | Flavobacteriales | Flavobacteriaceae | Flavobacterium |  |
| 8 | Proteobacteria | Alphaproteobacteria | Rhizobiales | Hyphomicrobiaceae | |  |
| 8 | Actinobacteria | Actinobacteria | Actinomycetales | Microbacteriaceae | Salinibacterium |  |
| 8 | Actinobacteria | Actinobacteria | Actinomycetales | Propionibacteriaceae | |  |
| 8 | Proteobacteria | Alphaproteobacteria | Rhizobiales | Rhizobiaceae | Agrobacterium |  |
| 8 | Bacteroidetes | Sphingobacteriia | Sphingobacteriales | Saprospiraceae |  |  |
| 8 | Firmicutes | Bacilli | Bacillales | Staphylococcaceae | Staphylococcus | epidermidis |
| 8 | Proteobacteria** | Alphaproteobacteria | Rhizobiales |  |  |  |
| 8 | Proteobacteria |  |  |  |  |  |
| 8 | Proteobacteria | Alphaproteobacteria | Rhizobiales |  |  |  |
| 8 | Proteobacteria | Alphaproteobacteria | BD7-3 |  |  |  |
| 8 | Bacteroidetes* | Sphingobacteriia | Sphingobacteriales | |  |  |
| 9 | Proteobacteria | Alphaproteobacteria | Rhizobiales | Bradyrhizobiaceae |  |  |
| 9 | Proteobacteria | Epsilonproteobacteria | Campylobacterales | Campylobacteraceae | Arcobacter |  |
| 9 | Bacteroidetes | Sphingobacteriia | Sphingobacteriales | Chitinophagaceae |  |  |
| 9 | Proteobacteria | Betaproteobacteria | Burkholderiales | Comamonadaceae |  |  |
| 9 | Actinobacteria* | Actinobacteria | Actinomycetales | Nocardioidaceae | Nocardioides |  |
| 9 | Proteobacteria** | Betaproteobacteria | Burkholderiales | Oxalobacteraceae |  |  |
| 9 | Firmicutes* | Bacilli | Lactobacillales | Streptococcaceae |  |  |
| 9 | Proteobacteria | Gammaproteobacteria | Xanthomonadales | Xanthomonadaceae | |  |
| 9 | Proteobacteria* | Alphaproteobacteria | Sphingomonadales | |  |  |
| 10 | Bacteroidetes | Sphingobacteriia | Sphingobacteriales | Chitinophagaceae |  |  |
| 10 | Actinobacteria** | Actinobacteria | Actinomycetales | Corynebacteriaceae | Corynebacterium | |
| 10 | Proteobacteria** | Gammaproteobacteria | Pasteurellales | Pasteurellaceae | Haemophilus |  |
| 10 | Proteobacteria | Betaproteobacteria | Burkholderiales | Comamonadaceae | Methylibium |  |
| 10 | Proteobacteria | Gammaproteobacteria | Enterobacteriales | Enterobacteriaceae | |  |
| 10 | Bacteroidetes | Flavobacteriia | Flavobacteriales | Flavobacteriaceae | Flavobacterium |  |
| 10 | Proteobacteria | Alphaproteobacteria | Rhodobacterales | Hyphomonadaceae | |  |
| 10 | Actinobacteria* | Actinobacteria | Actinomycetales | Microbacteriaceae |  |  |
| 10 | Proteobacteria | Betaproteobacteria | Burkholderiales | Oxalobacteraceae |  |  |
| 10 | Proteobacteria | Alphaproteobacteria | Rhodobacterales | Rhodobacteraceae |  |  |
| 10 | Proteobacteria | Alphaproteobacteria | Rhodobacterales | Rhodobacteraceae |  |  |
| 10 | Proteobacteria | Alphaproteobacteria | Rhodobacterales | Rhodobacteraceae | Rhodobacter |  |
| 10 | Proteobacteria** | Betaproteobacteria | Rhodocyclales | Rhodocyclaceae | Dechloromonas |  |
| 11 | Proteobacteria | Alphaproteobacteria | Rhizobiales | Beijerinckiaceae |  |  |
| 11 | Bacteroidetes | Sphingobacteriia | Sphingobacteriales | Chitinophagaceae |  |  |
| 11 | Proteobacteria | Betaproteobacteria | Burkholderiales | Comamonadaceae | Polaromonas |  |
| 11 | Proteobacteria | Betaproteobacteria | Burkholderiales | Comamonadaceae |  |  |
| 11 | Proteobacteria* | Betaproteobacteria | Burkholderiales | Comamonadaceae |  |  |
| 11 | Proteobacteria | Alphaproteobacteria | Rhodobacterales | Hyphomonadaceae | |  |
| 11 | Bacteroidetes | Sphingobacteriia | Sphingobacteriales | Saprospiraceae |  |  |
| 11 | Proteobacteria | Gammaproteobacteria | Xanthomonadales | Xanthomonadaceae | |  |
| 11 | Proteobacteria | Gammaproteobacteria | Xanthomonadales | Xanthomonadaceae | |  |
| 11 | Proteobacteria | Alphaproteobacteria | Rhizobiales |  |  |  |
| 12 | Actinobacteria | Actinobacteria | Actinomycetales | Actinomycetaceae | Actinomyces |  |
| 12 | Actinobacteria | Acidimicrobiia | Acidimicrobiales | C111 |  |  |
| 12 | Proteobacteria** | Alphaproteobacteria | Caulobacterales | Caulobacteraceae Caulobacter | |  |
| 12 | Bacteroidetes** | Flavobacteriia | Flavobacteriales | Flavobacteriaceae | Flavobacterium |  |
| 12 | Bacteroidetes | Sphingobacteriia | Sphingobacteriales | Flexibacteraceae |  |  |
| 12 | Proteobacteria | Betaproteobacteria | Burkholderiales | Oxalobacteraceae |  |  |
| 12 | Actinobacteria | Actinobacteria | Actinomycetales | Propionibacteriaceae | |  |
| 12 | Bacteroidetes | Sphingobacteriia | Sphingobacteriales | Saprospiraceae | Haliscomenobacter | |
| 12 | Proteobacteria | Alphaproteobacteria | Sphingomonadales | Sphingomonadaceae | Sphingobium |  |
| 12 | Proteobacteria | Alphaproteobacteria | Sphingomonadales | |  |  |
| 13 | Proteobacteria | Epsilonproteobacteria | Campylobacterales | Campylobacteraceae | Campylobacter |  |
| 13 | Proteobacteria** | Betaproteobacteria | Burkholderiales | Burkholderiaceae Limnobacter | |  |
| 13 | Proteobacteria | Betaproteobacteria | Burkholderiales | Oxalobacteraceae |  |  |
| 13 | Proteobacteria | Gammaproteobacteria | Xanthomonadales | Xanthomonadaceae | Luteimonas |  |
| 13 | Proteobacteria | Deltaproteobacteria | Myxococcales |  |  |  |
| 13 | TM7 | TM7-2 |  |  |  |  |
| 13 | Proteobacteria | Alphaproteobacteria | |  |  |  |
| 13 | Proteobacteria | Alphaproteobacteria | |  |  |  |
| 14 | Proteobacteria | Deltaproteobacteria | Bdellovibrionales | Bdellovibrionaceae | Bdellovibrio |  |
| 14 | Proteobacteria | Gammaproteobacteria | Aeromonadales | Aeromonadaceae | Aeromonas |  |
| 14 | Proteobacteria | Betaproteobacteria | Burkholderiales | Comamonadaceae |  |  |
| 14 | Bacteroidetes | Flavobacteriia | Flavobacteriales | Flavobacteriaceae | Flavobacterium |  |
| 14 | Proteobacteria | Gammaproteobacteria | Pseudomonadales | Moraxellaceae | Enhydrobacter |  |
| 14 | Bacteroidetes** | Sphingobacteriia | Sphingobacteriales | Sphingobacteriaceae | Pedobacter |  |
| 14 | Proteobacteria | Betaproteobacteria | |  |  |  |
| 15 | Bacteroidetes | Flavobacteriia | Flavobacteriales | Flavobacteriaceae | Flavobacterium |  |
| 15 | Proteobacteria | Alphaproteobacteria | Rhizobiales | Hyphomicrobiaceae | Devosia |  |
| 15 | Firmicutes | Bacilli | Lactobacillales | Streptococcaceae |  |  |
| 15 | Bacteroidetes | Sphingobacteriia | Sphingobacteriales | |  |  |
| 15 | Proteobacteria | Betaproteobacteria | SBla14 |  |  |  |
| 16 | Proteobacteria | Betaproteobacteria | Burkholderiales | Comamonadaceae | Rhodoferax |  |
| 16 | Proteobacteria | Betaproteobacteria | Burkholderiales | Oxalobacteraceae |  |  |
| 17 | Bacteroidetes* | Flavobacteriia | Flavobacteriales | Flavobacteriaceae | Flavobacterium |  |
| 17 | Firmicutes | Bacilli | Lactobacillales | Streptococcaceae |  |  |
| 17 | Bacteroidetes | Sphingobacteriia | Sphingobacteriales | |  |  |
| 19 | Proteobacteria | Betaproteobacteria | Burkholderiales | Comamonadaceae | Hydrogenophaga |  |
| 19 | Proteobacteria | Alphaproteobacteria | Rhizobiales | Hyphomicrobiaceae | Devosia |  |
| 19 | Firmicutes | Bacilli | Lactobacillales | Lactobacillaceae | Lactobacillus |  |
| 19 | Bacteroidetes | Sphingobacteriia | Sphingobacteriales | |  |  |
| 20 | Proteobacteria | Alphaproteobacteria | Sphingomonadales | Sphingomonadaceae | |  |
| 20 | Proteobacteria | Gammaproteobacteria | Xanthomonadales | Xanthomonadaceae | Dokdonella |  |
| 20 | Acidobacteria | Chloracidobacteria | |  |  |  |
| 20 | OD1* | ZB2 |  |  |  |  |
| 21 | Proteobacteria | Betaproteobacteria | Burkholderiales | Comamonadaceae | Comamonas |  |
| 21 | Proteobacteria | Alphaproteobacteria | Rhizobiales | Hyphomicrobiaceae | Devosia |  |
| 21 | Bacteroidetes | Sphingobacteriia | Sphingobacteriales | |  |  |
| 22 | Proteobacteria | Betaproteobacteria | Burkholderiales | Comamonadaceae |  |  |
| 22 | Proteobacteria | Betaproteobacteria | Burkholderiales | Comamonadaceae | Comamonas |  |
| 22 | Proteobacteria | Betaproteobacteria | Methylophilales | Methylophilaceae | Methylotenera | mobilis |
| 22 | Proteobacteria | Alphaproteobacteria | Rhodobacterales | Rhodobacteraceae |  |  |
| 22 | Firmicutes | Bacilli | Lactobacillales | Streptococcaceae | Streptococcus |  |
| 23 | Bacteroidetes | Bacteroidia | Bacteroidales | Bacteroidaceae | Bacteroides |  |
| 23 | Bacteroidetes | Sphingobacteriia | Sphingobacteriales | Chitinophagaceae |  |  |
| 23 | Bacteroidetes | Flavobacteriia | Flavobacteriales | Flavobacteriaceae | Flavobacterium |  |
| 23 | Proteobacteria | Alphaproteobacteria | Rhizobiales | Hyphomicrobiaceae | Devosia |  |
| 23 | Proteobacteria | Alphaproteobacteria | Rhodobacterales | Rhodobacteraceae |  |  |
| 23 | Proteobacteria | Betaproteobacteria | Methylophilales |  |  |  |
| 24 | Bacteroidetes | Flavobacteriia | Flavobacteriales | Flavobacteriaceae | Chryseobacterium | |
| 24 | Proteobacteria | Betaproteobacteria | Neisseriales | Neisseriaceae |  |  |
| 24 | Proteobacteria | Deltaproteobacteria | Myxococcales |  |  |  |
| 25 | Proteobacteria** | Alphaproteobacteria | Rhizobiales | Brucellaceae | Pseudochrobactrum | |
| 25 | Bacteroidetes | Sphingobacteriia | Sphingobacteriales | Saprospiraceae |  |  |
| 25 | Bacteroidetes* | Sphingobacteriia | Sphingobacteriales | Sphingobacteriaceae | Pedobacter |  |
| 25 | Proteobacteria | Alphaproteobacteria | Rickettsiales |  |  |  |
| 26 | Proteobacteria | Alphaproteobacteria | Rhodobacterales | Rhodobacteraceae | Rhodobacter |  |
| 27 | Proteobacteria | Betaproteobacteria | Burkholderiales | Comamonadaceae | Hydrogenophaga |  |
| 27 | Proteobacteria | Alphaproteobacteria | Rhizobiales | Hyphomicrobiaceae | |  |
| 28 | Proteobacteria | Betaproteobacteria | Burkholderiales | Comamonadaceae | Paucibacter |  |
| 28 | Firmicutes | Bacilli | Lactobacillales | Leuconostocaceae | Carnobacterium |  |
| 28 | Proteobacteria | Betaproteobacteria | Neisseriales | Neisseriaceae | Vitreoscilla |  |
| 29 | Proteobacteria | Alphaproteobacteria | Rhodobacterales | Rhodobacteraceae |  |  |
| 30 | Proteobacteria | Epsilonproteobacteria | Campylobacterales | Campylobacteraceae | Sulfurospirillum |  |
| 30 | Proteobacteria | Betaproteobacteria | Burkholderiales | Comamonadaceae | Limnohabitans |  |
| 30 | Bacteroidetes | Sphingobacteriia | Sphingobacteriales | Flexibacteraceae | Emticicia |  |
| 30 | Bacteroidetes** | Sphingobacteriia | Sphingobacteriales | Sphingobacteriaceae | Sphingobacterium | faecium |
| 31 | Actinobacteria | Actinobacteria | Actinomycetales | Propionibacteriaceae | Propionibacterium | acnes |
| 31 | Bacteroidetes | Sphingobacteriia | Sphingobacteriales | Saprospiraceae |  |  |
| 32 | Proteobacteria | Alphaproteobacteria | Rhodobacterales | Rhodobacteraceae | Rhodobacter |  |
| 32 | Proteobacteria | Alphaproteobacteria | Rhodobacterales | Rhodobacteraceae | Rhodobacter |  |
| 32 | Proteobacteria | Alphaproteobacteria | Sphingomonadales | Sphingomonadaceae | Novosphingobium | |
| 33 | Cyanobacteria | Chloroplast | Streptophyta |  |  |  |
| 35 | Bacteroidetes | Sphingobacteriia | Sphingobacteriales | Chitinophagaceae |  |  |
| 35 | Bacteroidetes | Flavobacteriia | Flavobacteriales | Flavobacteriaceae |  |  |
| 35 | Bacteroidetes | Flavobacteriia | Flavobacteriales | Flavobacteriaceae | Flavobacterium |  |
| 35 | Proteobacteria | Alphaproteobacteria | Rhizobiales |  |  |  |
| 36 | Proteobacteria | Betaproteobacteria | Burkholderiales | Comamonadaceae | Rhodoferax |  |
| 36 | Proteobacteria | Betaproteobacteria | Burkholderiales | Comamonadaceae | Methylibium |  |
| 37 | Proteobacteria | Alphaproteobacteria | Rhodobacterales | Rhodobacteraceae | Rhodobacter |  |
| 38 | Proteobacteria | Alphaproteobacteria | Sphingomonadales | |  |  |
| 38 | Proteobacteria | Betaproteobacteria | YCC11 |  |  |  |
| 39 | Proteobacteria | Betaproteobacteria | Burkholderiales | Comamonadaceae |  |  |
| 39 | Proteobacteria | Betaproteobacteria | Burkholderiales | Comamonadaceae |  |  |
| 39 | Bacteroidetes | Flavobacteriia | Flavobacteriales | Flavobacteriaceae | Flavobacterium |  |
| 39 | Proteobacteria | Betaproteobacteria | Neisseriales | Neisseriaceae | Vogesella |  |
| 40 | Proteobacteria | Alphaproteobacteria | Caulobacterales | Caulobacteraceae | Mycoplana |  |
| 40 | Bacteroidetes | Flavobacteriia | Flavobacteriales | Flavobacteriaceae | Flavobacterium |  |
| 40 | TM7 | TM7-3 |  |  |  |  |
| 41 | Proteobacteria | Alphaproteobacteria | Rhizobiales |  |  |  |
| 42 | Proteobacteria | Alphaproteobacteria | Rhodobacterales | Rhodobacteraceae | Rhodobacter |  |
| 42 | Proteobacteria | Gammaproteobacteria | Xanthomonadales | Xanthomonadaceae | Thermomonas |  |
| 42 | Proteobacteria | Gammaproteobacteria | |  |  |  |
| 43 | Bacteroidetes | Sphingobacteriia | Sphingobacteriales | Chitinophagaceae |  |  |
| 44 | Proteobacteria | Alphaproteobacteria | Sphingomonadales | Sphingomonadaceae | |  |
| 45 | Proteobacteria | Gammaproteobacteria | Aeromonadales | Aeromonadaceae |  |  |
| 45 | Proteobacteria | Alphaproteobacteria | Rhodobacterales | Rhodobacteraceae | Rhodobacter |  |
| 45 | Proteobacteria | Alphaproteobacteria | Rhizobiales |  |  |  |
| 46 | Proteobacteria | Betaproteobacteria | Burkholderiales | Oxalobacteraceae |  |  |
| 46 | Proteobacteria | Alphaproteobacteria | Rhodobacterales | Rhodobacteraceae | Rhodobacter |  |
| 47 | Proteobacteria | Alphaproteobacteria | Rhizobiales | Hyphomicrobiaceae | Devosia |  |
| 48 | Bacteroidetes | Flavobacteriia | Flavobacteriales | Flavobacteriaceae | Chryseobacterium | |
| 51 | Bacteroidetes | Flavobacteriia | Flavobacteriales | Flavobacteriaceae | Flavobacterium |  |
| 52 | Bacteroidetes | Sphingobacteriia | Sphingobacteriales | Saprospiraceae | Haliscomenobacter | |
| 56 | Proteobacteria | Betaproteobacteria | |  |  |  |
| 57 | Bacteroidetes | Flavobacteriia | Flavobacteriales | Flavobacteriaceae | Flavobacterium |  |
| 59 | Bacteroidetes | Sphingobacteriia | Sphingobacteriales | Chitinophagaceae |  |  |
| 64 | Bacteroidetes | Flavobacteriia | Flavobacteriales | Flavobacteriaceae | Flavobacterium |  |
| 64 | Proteobacteria | Alphaproteobacteria | Rhizobiales | Rhizobiaceae | Agrobacterium |  |
| 65 | Proteobacteria | Betaproteobacteria | Rhodocyclales | Rhodocyclaceae | KD1-23 |  |
| 69 | Proteobacteria | Betaproteobacteria | Burkholderiales | Comamonadaceae | Comamonas |  |
| 69 | Proteobacteria | Alphaproteobacteria | Sphingomonadales | |  |  |
| 70 | Proteobacteria | Alphaproteobacteria | Rhodobacterales | Rhodobacteraceae | Rhodobacter |  |
| 71 | Proteobacteria | Gammaproteobacteria | Xanthomonadales | Xanthomonadaceae | Stenotrophomonas maltophilia | |
| 75 | Proteobacteria | Betaproteobacteria | Burkholderiales | Comamonadaceae |  |  |
| 80 | Bacteroidetes | Sphingobacteriia | Sphingobacteriales | Chitinophagaceae |  |  |
| 82 | Bacteroidetes | Flavobacteriia | Flavobacteriales | Cryomorphaceae |  |  |
| 83 | Proteobacteria | Betaproteobacteria | Burkholderiales | Comamonadaceae |  |  |
| 84 | Bacteroidetes | Sphingobacteriia | Sphingobacteriales | Flexibacteraceae |  |  |
| 87 | Firmicutes | Clostridia | Clostridiales | Veillonellaceae | Veillonella | dispar |
| 91 | Bacteroidetes | Sphingobacteriia | Sphingobacteriales | Chitinophagaceae |  |  |
| 97 | Proteobacteria | Alphaproteobacteria | Rhodobacterales | Rhodobacteraceae | Rhodobacter |  |
| 106 | Bacteroidetes | Flavobacteriia | Flavobacteriales | Flavobacteriaceae | Flavobacterium |  |
| 107 | Actinobacteria | Actinobacteria | Actinomycetales | Microbacteriaceae | Leucobacter |  |
| 108 | Bacteroidetes | Sphingobacteriia | Sphingobacteriales | Sphingobacteriaceae | Pedobacter |  |
| 111 | Proteobacteria | Gammaproteobacteria | Pseudomonadales | Moraxellaceae | Acinetobacter |  |
| 114 | Bacteroidetes | Flavobacteriia | Flavobacteriales | Flavobacteriaceae | Chryseobacterium | |
| 117 | Proteobacteria | Gammaproteobacteria | Pseudomonadales | Pseudomonadaceae Pseudomonas | | fluorescens |
| 118 | Bacteroidetes | Sphingobacteriia | Sphingobacteriales | Sphingobacteriaceae | Pedobacter |  |
| 119 | Proteobacteria | Alphaproteobacteria | Rhodobacterales | Rhodobacteraceae | Rhodobacter |  |
| 123 | Bacteroidetes | Flavobacteriia | Flavobacteriales | Flavobacteriaceae | Flavobacterium |  |
| 124 | Proteobacteria | Gammaproteobacteria | Pseudomonadales | Moraxellaceae | Acinetobacter |  |
| 125 | Proteobacteria | Betaproteobacteria | Burkholderiales | Comamonadaceae | Comamonas |  |
| 126 | Proteobacteria | Alphaproteobacteria | Rhodobacterales | Rhodobacteraceae | Rhodobacter |  |
| 138 | Firmicutes | Bacilli | Lactobacillales | Enterococcaceae | Enterococcus |  |
| 145 | Proteobacteria | Betaproteobacteria | Burkholderiales | Oxalobacteraceae |  |  |
| 146 | Proteobacteria | Alphaproteobacteria | Sphingomonadales | Sphingomonadaceae | |  |
| 151 | Proteobacteria | Betaproteobacteria | Burkholderiales | Comamonadaceae |  |  |
| 161 | Proteobacteria | Gammaproteobacteria | Xanthomonadales | Xanthomonadaceae | |  |
| 190 | Bacteroidetes | Sphingobacteriia | Sphingobacteriales | Chitinophagaceae |  |  |
| 192 | Proteobacteria | Gammaproteobacteria | Pseudomonadales | Moraxellaceae | Enhydrobacter |  |
| 194 | Bacteroidetes** | Flavobacteriia | Flavobacteriales | Flavobacteriaceae | Flavobacterium | psychrophilum |
| 216 | Firmicutes | Clostridia | Clostridiales | Veillonellaceae | Veillonella |  |
| 221 | Proteobacteria | Betaproteobacteria | Neisseriales | Neisseriaceae | Microvirgula | aerodenitrificans |
| 228 | Proteobacteria | Epsilonproteobacteria | Campylobacterales | Campylobacteraceae | Arcobacter |  |
| 229 | Proteobacteria | Betaproteobacteria | Burkholderiales | Oxalobacteraceae | Janthinobacterium | lividum |
| 231 | Proteobacteria | Betaproteobacteria | Burkholderiales | Comamonadaceae | Rubrivivax |  |
| 247 | Proteobacteria | Gammaproteobacteria | Aeromonadales | Aeromonadaceae |  |  |
| 248 | Firmicutes | Bacilli | Lactobacillales | Streptococcaceae | Streptococcus | luteciae |
| 253 | Proteobacteria | Gammaproteobacteria | Enterobacteriales | Enterobacteriaceae | |  |
| 260 | Proteobacteria | Betaproteobacteria | Burkholderiales | Comamonadaceae | Acidovorax |  |
| 272 | Proteobacteria | Gammaproteobacteria | |  |  |  |
| 284 | Proteobacteria | Betaproteobacteria | Burkholderiales | Comamonadaceae | Methylibium |  |
| 286 | Proteobacteria | Betaproteobacteria | Burkholderiales | Comamonadaceae | Limnohabitans |  |
| 295 | Bacteroidetes | Flavobacteriia | Flavobacteriales | Flavobacteriaceae | Chryseobacterium | |
| 383 | Proteobacteria | Gammaproteobacteria | Pseudomonadales | Moraxellaceae | Acinetobacter |  |
| 397 | Bacteroidetes | Sphingobacteriia | Sphingobacteriales | |  |  |
| 406 | Bacteroidetes | Flavobacteriia | Flavobacteriales | Flavobacteriaceae | Flavobacterium |  |
| 416 | Bacteroidetes | Sphingobacteriia | Sphingobacteriales | Chitinophagaceae |  |  |
| 427 | Proteobacteria | Alphaproteobacteria | Rhizobiales |  |  |  |
| 452 | Proteobacteria | Gammaproteobacteria | Pseudomonadales | Moraxellaceae | Acinetobacter |  |
| 517 | Bacteroidetes | Flavobacteriia | Flavobacteriales | Flavobacteriaceae |  |  |
| 540 | Proteobacteria | Betaproteobacteria | Burkholderiales | Comamonadaceae | Rhodoferax |  |
| 609 | Proteobacteria | Gammaproteobacteria | Pseudomonadales | Moraxellaceae | Acinetobacter | johnsonii |
| 612 | Proteobacteria | Betaproteobacteria | Burkholderiales | Comamonadaceae | Limnohabitans |  |
| 697 | Proteobacteria | Betaproteobacteria | Burkholderiales | Comamonadaceae | Hydrogenophaga |  |
| 700 | Bacteroidetes | Sphingobacteriia | Sphingobacteriales | |  |  |
| 1160 | Proteobacteria | Betaproteobacteria | Burkholderiales | Comamonadaceae | Delftia |  |
| 1176 | Firmicutes | Bacilli | Lactobacillales | Streptococcaceae | Lactococcus | garvieae |
| 1189 | Firmicutes | Bacilli | Lactobacillales | Leuconostocaceae | Leuconostoc |  |
| 1303 | Proteobacteria | Betaproteobacteria | Burkholderiales | Oxalobacteraceae | Janthinobacterium | |
| 1413 | Firmicutes | Bacilli | Lactobacillales | Streptococcaceae | Lactococcus | garvieae |
| 1427 | Proteobacteria | Gammaproteobacteria | Enterobacteriales | Enterobacteriaceae | Citrobacter |  |
| 1514 | Firmicutes | Bacilli | Lactobacillales | Leuconostocaceae |  |  |
| 1605 | Proteobacteria | Gammaproteobacteria | Pseudomonadales | Moraxellaceae | Acinetobacter |  |
| 3481 | Proteobacteria | Betaproteobacteria | Burkholderiales | Comamonadaceae | Comamonas |  |
| 4286 | Bacteroidetes | Flavobacteriia | Flavobacteriales | Flavobacteriaceae | Flavobacterium |  |

* Bacterial sequences that were only found on eggs incubated at a mean temperature of < 4.5° Celsius.

** Bacterial sequences that were only found on eggs incubated at a mean temperature of > 4.5° Celsius.

Table S4: Spawning locations and unfiltered alpha diversity measures of bacterial communities on naturally spawned brown trout eggs.

| **Sample-ID** | **Location** | **Mean T^a^** | **PD^b^** | **Chao 1^c^** | **OS^d^** |
| --- | --- | --- | --- | --- | --- |
| 1 | Inn | 2.00/0.60 | 10.34 | 179 | 140 |
|
| 2 | Aare-Innertkirchen | 3.51/0.22 | 15.87 | 290.66 | 204 |
|
| 3 | Aare-Wichtrach | 4.75/0.29 | 24.56 | 464.66 | 344 |
|
| 4 | Aare-Belp | 4.73/0.30 | 12.73 | 226 | 181 |
|
| 5 | ^*^Aare-Lerbestrasse | 4.85/1.40 | 15.84 | 257.39 | 188 |
|
| 6 | Aare-Zehndermätteli | 4.00/1.91 | 15.92 | 333.55 | 223 |
|
| 7 | Worble | 5.79/0.73 | 29.64 | 562.04 | 423 |
|
| 8 | Amletebach | 3.01/2.11 | 14.04 | 277 | 200 |
|
| 9 | ^*^Gürbe | 2.67/1.94 | 19.25 | 427.23 | 330 |
|

^a^Mean water temperature during the incubation of the eggs at natural spawning places and its standart deviation. ^b^Mean alpha diversity measure “phylogenetic distance”, ^c^”Chao 1”, and ^d^“observed number of species”. All three alpha diversity measures were estimated without filtering out bacterial taxa that were found in the negative control of beads and buffers. ^*^At these two locations water samples had been collected simultaneously.

Table S5: KEGG legend.

PICRUSt v.1.0.0^57^ metagenome prediction predicted higher abundance of genes in 69 pathways of brown trout egg-associated bacterial communities relative to corresponding water samples. Here we show annotated KEGG pathways at the third hierarchical level with detailed descriptions extending Table 2 in the main manuscript and Fig. S6.

| **KEGG Orthologs** | **KEGG genes / enzymes^a^** | **KEGG pathways** |
| --- | --- | --- |
| K00605 | gcvT; aminomethyltransferase [EC:2.1.2.10] | Metabolic pathways |
| K00605 | gcvT; aminomethyltransferase [EC:2.1.2.10] | Biosynthesis of secondary metabolites |
| K00605 | gcvT; aminomethyltransferase [EC:2.1.2.10] | Biosynthesis of antibiotics |
| K00605 | gcvT; aminomethyltransferase [EC:2.1.2.10] | Carbon metabolism |
| K00605 | gcvT; aminomethyltransferase [EC:2.1.2.10] | One carbon pool by folate |
| K00605 | gcvT; aminomethyltransferase [EC:2.1.2.10] | Glycine, serine and threonine metabolism |
| K00605 | gcvT; aminomethyltransferase [EC:2.1.2.10] | Glyoxylate and dicarboxylate metabolism |
| K07444 | ypsC; putative N6-adenine-specific DNA methylase [EC:2.1.1.-] | no pathway |
| K01893 | NARS; asparaginyl-tRNA synthetase [EC:6.1.1.22] | Aminoacyl-tRNA biosynthesis |
| K03306 | TC.PIT; inorganic phosphate transporter, PiT family | no pathway |
| K00334 | nuoE; NADH-quinone oxidoreductase subunit E [EC:1.6.5.3] | Metabolic pathways |
| K00334 | nuoE; NADH-quinone oxidoreductase subunit E [EC:1.6.5.3] | Oxidative phosphorylation |
| K05520 | pfpI; protease I [EC:3.2.-.-] | no pathway |
| K00335 | nuoF; NADH-quinone oxidoreductase subunit F [EC:1.6.5.3] | Metabolic pathways |
| K00335 | nuoF; NADH-quinone oxidoreductase subunit F [EC:1.6.5.3] | Oxidative phosphorylation |
| K04653 | hypC; hydrogenase expression/formation protein HypC | no pathway |
| K09808 | ABC.LPT.P; lipoprotein-releasing system permease protein | ABC transporters |
| K01515 | nudF; ADP-ribose pyrophosphatase [EC:3.6.1.13] | Purine metabolism |
| K09014 | sufB; Fe-S cluster assembly protein SufB | no pathway |
| K07568 | queA; S-adenosylmethionine:tRNA ribosyltransferase-isomerase [EC:2.4.99.17] | no pathway |
| K03529 | smc; chromosome segregation protein | no pathway |
| K01783 | rpe; ribulose-phosphate 3-epimerase [EC:5.1.3.1] | Metabolic pathways |
| K01783 | rpe; ribulose-phosphate 3-epimerase [EC:5.1.3.1] | Biosynthesis of secondary metabolites |
| K01783 | rpe; ribulose-phosphate 3-epimerase [EC:5.1.3.1] | Biosynthesis of antibiotics |
| K01783 | rpe; ribulose-phosphate 3-epimerase [EC:5.1.3.1] | Carbon metabolism |
| K01783 | rpe; ribulose-phosphate 3-epimerase [EC:5.1.3.1] | Microbial metabolism in diverse environments |
| K01783 | rpe; ribulose-phosphate 3-epimerase [EC:5.1.3.1] | Biosynthesis of amino acids |
| K01783 | rpe; ribulose-phosphate 3-epimerase [EC:5.1.3.1] | Pentose phosphate pathway |
| K01783 | rpe; ribulose-phosphate 3-epimerase [EC:5.1.3.1] | Pentose and glucuronate interconversions |
| K01783 | rpe; ribulose-phosphate 3-epimerase [EC:5.1.3.1] | Carbon fixation in photosynthetic organisms |
| K08963 | mtnA; methylthioribose-1-phosphate isomerase [EC:5.3.1.23] | Metabolic pathways |
| K08963 | mtnA; methylthioribose-1-phosphate isomerase [EC:5.3.1.23] | Cysteine and methionine metabolism |
| K00339 | nuoJ; NADH-quinone oxidoreductase subunit J [EC:1.6.5.3] | Metabolic pathways |
| K00339 | nuoJ; NADH-quinone oxidoreductase subunit J [EC:1.6.5.3] | Oxidative phosphorylation |
| K06997 | no entry | no pathway |
| K06883 | no entry | no pathway |
| K03555 | mutS; DNA mismatch repair protein MutS | Mismatch repair |
| K00616 | transaldolase [EC:2.2.1.2] | Metabolic pathways |
| K00616 | transaldolase [EC:2.2.1.2] | Biosynthesis of secondary metabolites |
| K00616 | transaldolase [EC:2.2.1.2] | Biosynthesis of antibiotics |
| K00616 | transaldolase [EC:2.2.1.2] | Carbon metabolism |
| K00616 | transaldolase [EC:2.2.1.2] | Microbial metabolism in diverse environments |
| K00616 | transaldolase [EC:2.2.1.2] | Biosynthesis of amino acids |
| K00616 | transaldolase [EC:2.2.1.2] | Pentose phosphate pathway |
| K05896 | scpA; segregation and condensation protein A | no pathway |
| K11175 | purN; phosphoribosylglycinamide formyltransferase 1 [EC:2.1.2.2] | Metabolic pathways |
| K11175 | purN; phosphoribosylglycinamide formyltransferase 1 [EC:2.1.2.2] | Biosynthesis of secondary metabolites |
| K11175 | purN; phosphoribosylglycinamide formyltransferase 1 [EC:2.1.2.2] | Biosynthesis of antibiotics |
| K11175 | purN; phosphoribosylglycinamide formyltransferase 1 [EC:2.1.2.2] | One carbon pool by folate |
| K11175 | purN; phosphoribosylglycinamide formyltransferase 1 [EC:2.1.2.2] | Purine metabolism |
| K12410 | npdA; NAD-dependent deacetylase [EC:3.5.1.-] | no pathway |
| K01644 | citE; citrate lyase subunit beta / citryl-CoA lyase [EC:4.1.3.34] | Two-component system |
| K01126 | glycerophosphoryl diester phosphodiesterase [EC:3.1.4.46] | Glycerophospholipid metabolism |
| K11717 | sufS; cysteine desulfurase / selenocysteine lyase [EC:2.8.1.7/4.4.1.16] | Metabolic pathways |
| K11717 | sufS; cysteine desulfurase / selenocysteine lyase [EC:2.8.1.7 4.4.1.16] | Selenocompound metabolism |
| K00111 | glpA; glycerol-3-phosphate dehydrogenase [EC:1.1.5.3] | Biosynthesis of secondary metabolites |
| K00111 | glpA; glycerol-3-phosphate dehydrogenase [EC:1.1.5.3] | Glycerophospholipid metabolism |
| K03593 | mrp, NUBPL; ATP-binding protein involved in chromosome partitioning | no pathway |
| K03638 | moaB; molybdenum cofactor biosynthesis protein B | no pathway |
| K07577 | putative mRNA 3-end processing factor | no pathway |
| K03530 | hupB; DNA-binding protein HU-beta | no pathway |
| K00573 | pcm; protein-L-isoaspartate(D-aspartate) O-methyltransferase [EC:2.1.1.77] | no pathway |
| K03782 | katG; catalase-peroxidase [EC:1.11.1.21] | Metabolic pathways |
| K03782 | katG; catalase-peroxidase [EC:1.11.1.21] | Biosynthesis of secondary metabolites |
| K03782 | katG; catalase-peroxidase [EC:1.11.1.21] | Tryptophan metabolism |
| K03782 | katG; catalase-peroxidase [EC:1.11.1.21] | Phenylalanine metabolism |
| K03782 | katG; catalase-peroxidase [EC:1.11.1.21] | Phenylpropanoid biosynthesis |
| K06941 | rlmN; 23S rRNA (adenine2503-C2)-methyltransferase [EC:2.1.1.192] | no pathway |
| K04656 | hypF; hydrogenase maturation protein HypF | no pathway |
| K01153 | hsdR; type I restriction enzyme, R subunit [EC:3.1.21.3] | no pathway |
| K04567 | KARS; lysyl-tRNA synthetase, class II [EC:6.1.1.6] | Aminoacyl-tRNA biosynthesis |
| K13038 | coaBC; phosphopantothenoylcysteine decarboxylase [EC:4.1.1.36/6.3.2.5] | Metabolic pathways |
| K11754 | folC; dihydrofolate synthase / folylpolyglutamate synthase [EC:6.3.2.12/6.3.2.17] | Metabolic pathways |
| K11754 | folC; dihydrofolate synthase / folylpolyglutamate synthase [EC:6.3.2.12/6.3.2.17] | Folate biosynthesis |
| K01840 | manB; phosphomannomutase [EC:5.4.2.8] | Metabolic pathways |
| K01840 | manB; phosphomannomutase [EC:5.4.2.8] | Biosynthesis of secondary metabolites |
| K01840 | manB; phosphomannomutase [EC:5.4.2.8] | Fructose and mannose metabolism |
| K01840 | manB; phosphomannomutase [EC:5.4.2.8] | Amino sugar and nucleotide sugar metabolism |
| K03427 | hsdM; type I restriction enzyme M protein [EC:2.1.1.72] | no pathway |
| K09013 | sufC; Fe-S cluster assembly ATP-binding protein | no pathway |
| K03637 | moaC; cyclic pyranopterin phosphate synthase [EC:4.1.99.18] | Metabolic pathways |
| K03637 | moaC; cyclic pyranopterin phosphate synthase [EC:4.1.99.18] | Folate biosynthesis |
| K03637 | moaC; cyclic pyranopterin phosphate synthase [EC:4.1.99.18] | Sulfur relay system |
| K04654 | hypD; hydrogenase expression/formation protein HypD | no pathway |
| K00332 | nuoC; NADH-quinone oxidoreductase subunit C [EC:1.6.5.3] | Metabolic pathways |
| K00332 | nuoC; NADH-quinone oxidoreductase subunit C [EC:1.6.5.3] | Oxidative phosphorylation |
| K01491 | folD; methylenetetrahydrofolate dehydrogenase (NADP+) [EC:1.5.1.5/3.5.4.9] | Metabolic pathways |
| K01491 | folD; methylenetetrahydrofolate dehydrogenase (NADP+) / methenyltetra hydrofolate cyclohydrolase [EC:1.5.1.5/3.5.4.9] | Carbon metabolism |
| K01491 | folD; methylenetetrahydrofolate dehydrogenase (NADP+) / methenyltetra hydrofolate cyclohydrolase [EC:1.5.1.5/3.5.4.9] | Microbial metabolism in diverse environments |
| K01491 | folD; methylenetetrahydrofolate dehydrogenase (NADP+) / methenyltetra hydrofolate cyclohydrolase [EC:1.5.1.5/3.5.4.9] | One carbon pool by folate |
| K01491 | folD; methylenetetrahydrofolate dehydrogenase (NADP+) / methenyltetra hydrofolate cyclohydrolase [EC:1.5.1.5/3.5.4.9] | Carbon fixation pathways in prokaryotes |
| K08978 | TC.BAT1; bacterial/archaeal transporter family protein | no pathway |
| K00602 | purH; phosphoribosylaminoimidazolecarboxamide formyltransferase, cyclohydrolase [EC:2.1.2.3/3.5.4.10] | Metabolic pathways |
| K00602 | purH; phosphoribosylaminoimidazolecarboxamide formyltransferase / IMP cyclohydrolase [EC:2.1.2.3/3.5.4.10] | Biosynthesis of secondary metabolites |
| K00602 | purH; phosphoribosylaminoimidazolecarboxamide formyltransferase / IMP cyclohydrolase [EC:2.1.2.3/3.5.4.10] | Biosynthesis of antibiotics |
| K00602 | purH; phosphoribosylaminoimidazolecarboxamide formyltransferase /IMP cyclohydrolase [EC:2.1.2.3/3.5.4.10] | One carbon pool by folate |
| K00602 | purH; phosphoribosylaminoimidazolecarboxamide formyltransferase / IMP cyclohydrolase [EC:2.1.2.3/3.5.4.10] | Purine metabolism |
| K04651 | hypA, hybF; hydrogenase nickel incorporation protein HypA/HybF | no pathway |
| K03686 | dnaJ; molecular chaperone DnaJ | no pathway |
| K00303 | soxB; sarcosine oxidase, subunit beta [EC:1.5.3.1] | Metabolic pathways |
| K00303 | soxB; sarcosine oxidase, subunit beta [EC:1.5.3.1] | Glycine, serine and threonine metabolism |
| K07047 | no entry | no pathway |
| K02032 | ABC.PE.A1; peptide/nickel transport system ATP-binding protein | no pathway |
| K01738 | cysK; cysteine synthase A [EC:2.5.1.47] | Metabolic pathways |
| K01738 | cysK; cysteine synthase A [EC:2.5.1.47] | Biosynthesis of secondary metabolites |
| K01738 | cysK; cysteine synthase A [EC:2.5.1.47] | Biosynthesis of antibiotics |
| K01738 | cysK; cysteine synthase A [EC:2.5.1.47] | Carbon metabolism |
| K01738 | cysK; cysteine synthase A [EC:2.5.1.47] | Microbial metabolism in diverse environments |
| K01738 | cysK; cysteine synthase A [EC:2.5.1.47] | Biosynthesis of amino acids |
| K01738 | cysK; cysteine synthase A [EC:2.5.1.47] | Cysteine and methionine metabolism |
| K01738 | cysK; cysteine synthase A [EC:2.5.1.47] | Sulfur metabolism |
| K03639 | MOCS1; cyclic pyranopterin phosphate synthase [EC:4.1.99.18] | Metabolic pathways |
| K03639 | MOCS1; cyclic pyranopterin phosphate synthase [EC:4.1.99.18] | Folate biosynthesis |
| K03639 | MOCS1; cyclic pyranopterin phosphate synthase [EC:4.1.99.18] | Sulfur relay system |
| K01572 | oadB; oxaloacetate decarboxylase, beta subunit [EC:4.1.1.3] | Metabolic pathways |
| K01572 | oadB; oxaloacetate decarboxylase, beta subunit [EC:4.1.1.3] | Pyruvate metabolism |
| K03568 | tldD; TldD protein | no pathway |
| K05592 | deaD; ATP-dependent RNA helicase DeaD [EC:3.6.4.13] | RNA degradation |
| K01953 | asnB; asparagine synthase (glutamine-hydrolysing) [EC:6.3.5.4] | Metabolic pathways |
| K01953 | asnB; asparagine synthase (glutamine-hydrolysing) [EC:6.3.5.4] | Biosynthesis of secondary metabolites |
| K01953 | asnB; asparagine synthase (glutamine-hydrolysing) [EC:6.3.5.4] | Alanine, aspartate and glutamate metabolism |
| K01953 | asnB; asparagine synthase (glutamine-hydrolysing) [EC:6.3.5.4] | [Alanine, aspartate and glutamate metabolism](http://www.genome.jp/kegg-bin/show_pathway?14349011478630/ko00250.args) |
| K01953 | asnB; asparagine synthase (glutamine-hydrolysing) [EC:6.3.5.4] | [Biosynthesis of secondary metabolites](http://www.genome.jp/kegg-bin/show_pathway?14349011478630/ko01110.args) |
| K01953 | asparagine synthase (glutamine-hydrolysing) [EC:6.3.5.4] | [Metabolic pathways](http://www.genome.jp/kegg-bin/show_pathway?14349011478630/ko01100.args) |
| K09133 | no entry | no pathway |
| K00302 | soxA; sarcosine oxidase, subunit alpha [EC:1.5.3.1] | Metabolic pathways |
| K00302 | soxA; sarcosine oxidase, subunit alpha [EC:1.5.3.1] | Glycine, serine and threonine metabolism |
| K07087 | no entry | no pathway |
| K01745 | hutH; histidine ammonia-lyase [EC:4.3.1.3] | Metabolic pathways |
| K01745 | hutH; histidine ammonia-lyase [EC:4.3.1.3] | Histidine metabolism |
| K09155 | no entry | no pathway |
| K11928 | putP; sodium/proline symporter | no pathway |
| K06218 | relE, stbE; mRNA interferase RelE/StbE | no pathway |
| K07397 | yhfA; putative redox protein | no pathway |
| K00974 | cca; tRNA nucleotidyltransferase (CCA-adding enzyme) [EC:2.7.7.72/3.1.3.4] | RNA transport |
| K01560 | 2-haloacid dehalogenase [EC:3.8.1.2] | Metabolic pathways |
| K01560 | 2-haloacid dehalogenase [EC:3.8.1.2] | Microbial metabolism in diverse environments |
| K01560 | 2-haloacid dehalogenase [EC:3.8.1.2] | Phlorocyclohexane and chlorobenzene degradation |
| K01560 | 2-haloacid dehalogenase [EC:3.8.1.2] | Chloroalkane and chloroalkene degradation |
| K03592 | pmbA; PmbA protein | no pathway |
| K13038 | coaBC; phosphopantothenoylcysteine decarboxylase / phosphopantothenate cysteine ligase [EC:4.1.1.36/6.3.2.5] | Pantothenate and CoA biosynthesis |

^a^Overrepresented genes and enzymes are shown with KEGG ID and abbreviation.

Table S6: Distance matrix showing pairwise distances between all spawning locations.

| **Sample names^a^** | **Temperature differences (°C)** | **UniFrac distances** | **UniFrac distances (unfiltered)** | **Geographic distances (km)** | **Corrected geographic distances (km)^b^** |
| --- | --- | --- | --- | --- | --- |
| 1 to 2 | 0.50 | 0.51 | 0.41 | 94.50 | 116.82 |
| 1 to 3 | 2.29 | 0.59 | 0.59 | 136.31 | 166.21 |
| 1 to 4 | 0.85 | 0.65 | 0.58 | 119.74 | 154.42 |
| 1 to 6 | 1.49 | 0.57 | 0.51 | 124.24 | 155.68 |
| 1 to 7 | 1.29 | 0.41 | 0.41 | 120.81 | 157.41 |
| 1 to 8 | 1.49 | 0.55 | 0.53 | 94.36 | 123.01 |
| 1 to 9 | 1.29 | 0.71 | 0.66 | 105.77 | 139.24 |
| 2 to 1 | 0.50 | 0.51 | 0.41 | 94.50 | 72.18 |
| 2 to 3 | 2.79 | 0.55 | 0.64 | 48.36 | 23.88 |
| 2 to 4 | 0.35 | 0.62 | 0.59 | 26.81 | 25.15 |
| 2 to 6 | 1.99 | 0.54 | 0.50 | 42.37 | 51.35 |
| 2 to 7 | 1.80 | 0.52 | 0.41 | 38.97 | 53.14 |
| 2 to 8 | 1.99 | 0.52 | 0.57 | 12.69 | 19.07 |
| 2 to 9 | 1.80 | 0.74 | 0.61 | 24.17 | 35.43 |
| 3 to 1 | 2.29 | 0.59 | 0.59 | 136.31 | 106.40 |
| 3 to 2 | 2.79 | 0.55 | 0.64 | 48.36 | 72.84 |
| 3 to 4 | 3.13 | 0.53 | 0.54 | 46.17 | 71.12 |
| 3 to 6 | 0.80 | 0.49 | 0.59 | 44.41 | 57.93 |
| 3 to 7 | 0.99 | 0.58 | 0.62 | 44.47 | 71.12 |
| 3 to 8 | 0.80 | 0.43 | 0.47 | 57.82 | 71.45 |
| 3 to 9 | 0.99 | 0.73 | 0.69 | 49.30 | 70.62 |
| 4 to 1 | 0.85 | 0.65 | 0.58 | 119.74 | 85.06 |
| 4 to 2 | 0.35 | 0.62 | 0.59 | 26.81 | 28.47 |
| 4 to 3 | 3.13 | 0.53 | 0.54 | 46.17 | 21.22 |
| 4 to 6 | 2.33 | 0.49 | 0.49 | 33.11 | 34.03 |
| 4 to 7 | 2.14 | 0.68 | 0.63 | 15.66 | 21.42 |
| 4 to 8 | 2.33 | 0.44 | 0.50 | 27.46 | 23.51 |
| 4 to 9 | 2.14 | 0.62 | 0.58 | 17.39 | 19.45 |
| 6 to 1 | 1.49 | 0.57 | 0.51 | 124.24 | 92.80 |
| 6 to 2 | 1.99 | 0.54 | 0.50 | 42.37 | 33.40 |
| 6 to 3 | 0.80 | 0.49 | 0.59 | 44.41 | 30.90 |
| 6 to 4 | 2.33 | 0.49 | 0.49 | 33.11 | 32.20 |
| 6 to 7 | 0.19 | 0.62 | 0.56 | 10.17 | 8.60 |
| 6 to 8 | 0.00 | 0.47 | 0.54 | 30.69 | 27.10 |
| 6 to 9 | 0.19 | 0.65 | 0.58 | 20.93 | 20.50 |
| 7 to 1 | 1.29 | 0.41 | 0.41 | 120.81 | 84.20 |
| 7 to 2 | 1.80 | 0.52 | 0.41 | 38.97 | 24.80 |
| 7 to 3 | 0.99 | 0.58 | 0.62 | 44.47 | 17.83 |
| 7 to 4 | 2.14 | 0.68 | 0.63 | 15.66 | 9.90 |
| 7 to 6 | 0.19 | 0.62 | 0.56 | 10.17 | 11.75 |
| 7 to 8 | 0.19 | 0.61 | 0.53 | 26.45 | 18.50 |
| 7 to 9 | 0.00 | 0.80 | 0.68 | 15.14 | 11.90 |
| 8 to 1 | 1.49 | 0.55 | 0.53 | 94.36 | 65.70 |
| 8 to 2 | 1.99 | 0.52 | 0.57 | 12.69 | 6.30 |
| 8 to 3 | 0.80 | 0.43 | 0.47 | 57.82 | 44.20 |
| 8 to 4 | 2.33 | 0.44 | 0.50 | 27.46 | 31.41 |
| 8 to 6 | 0.00 | 0.47 | 0.54 | 30.69 | 34.28 |
| 8 to 7 | 0.19 | 0.61 | 0.53 | 26.45 | 34.40 |
| 8 to 9 | 0.19 | 0.63 | 0.58 | 11.49 | 16.39 |
| 9 to 1 | 1.29 | 0.71 | 0.66 | 105.77 | 72.30 |
| 9 to 2 | 1.80 | 0.74 | 0.61 | 24.17 | 12.90 |
| 9 to 3 | 0.99 | 0.73 | 0.69 | 49.30 | 27.98 |
| 9 to 4 | 2.14 | 0.62 | 0.58 | 17.39 | 15.32 |
| 9 to 6 | 0.19 | 0.65 | 0.58 | 20.93 | 21.36 |
| 9 to 7 | 0.00 | 0.80 | 0.68 | 15.14 | 18.37 |
| 9 to 8 | 0.19 | 0.63 | 0.58 | 11.49 | 6.60 |

^a^Sample names correspond to Fig. 1 and Table 1 in the main manuscript and Table S1. For temperature, unweighted UniFrac distances and geographic distances, all distances were symmetric; *i.e.,* the distance between data point 1 and data point 2 is the same as the distance between data point 2 and data point 1. ^b^Unweighted UniFrac distances were also estimated from brown trout egg-associated bacterial communities without the removal of all bacterial taxa that had been found in the negative control of only beads and buffers. ^c^These geographic distances were increased uphill by including altitude differences between data points, therefore this distance matrix is asymmetric as the distance between data point 1 and data point 2 can be different from the distance between data point 2 and data point 1 depending on the slope of the river (see Supplemental Methods).

| **Sample size** | **Statistic** |  |  |
| --- | --- | --- | --- |
| (n Aare & n tributaries) | **Observed R^2^** | **Observed UniFrac distance** | |
| **4 & 3** | 0.178 | 0.556 |  |
|  | **Simulated mean R^2^** | **Simulated mean UniFrac distance [95% CI]** | **Percentage significant** |
| **5 & 4** | 0.255 | 0.522 [0.501-0.543] | 0.028 |
| **6 & 5** | 0.191 | 0.534 [0.527-0.541] | 0.052 |
| **7 & 6** | 0.238 | 0.524 [0.517-0.531] | 0.085 |
| **8 & 7** | 0.157 | 0.533 [0.528-0.539] | 0.047 |
| **9 & 8** | 0.157 | 0.529 [0.523-0.534] | 0.061 |
| **10 & 9** | 0.129 | 0.527 [0.521-0.532] | 0.066 |
| **11 & 10** | 0.121 | 0.530 [0.525-0.534] | 0.057 |
| **12 & 11** | 0.117 | 0.531 [0.527-0.535] | 0.047 |
| **13 & 12** | 0.111 | 0.528 [0.524-0.532] | 0.019 |
| **14 & 13** | 0.092 | 0.524 [0.522-0.526] | 0.028 |
| **15 & 14** | 0.082 | 0.526 [0.524-0.528] | 0.018 |

Table S7: Bootstrapping approach showing the effect of sample size within groups on non-parametric analysis of variance estimates

Four spawning sites in the main river Aare (sample ID 3 = Aare-Wichtrach, 4 = Aare-Belp, 5 = Aare-Lerbestrasse, and 6 = Aare-Zehndermätteli) were compared to three spawning sites in its tributaries (7 = Worble, 8 = Amletebach, and 9 = Gürbe). No significant difference could be detected between the two groups using non-parametric analyses of variance testing^20^ (adonis; see results main manuscript). Here, simulations were performed in order to investigate the effect of sample size within groups on significance of non-parametric analyses of variance testing. The number of samples within groups was increased in one step increments and 1,000 adonis tests were run for each increment to estimate mean R-square values, UniFrac distances, their 95% confidence intervals, and the percentage of tests that would turn out significantly different between the two groups. Synthetic bacterial communities were simulated by sampling with replacement (bootstrapping) from the distribution of bacterial taxa that had been found in Aare and its tributary samples, respectively (see Supplemental Material).

Table S8: Multiple regression analysis testing the association of average water temperatures of five equal time periods from spawning until sampling and alpha diversities of bacterial communities on brown trout eggs.

|  | **Parameter estimate (SE)** | ***t*** | ***p*** |
| --- | --- | --- | --- |
| a) Chao 1^a^ |  |  |  |
| Intercept | 316.3(54.9) | 5.7 | 0.001 |
| Period 1 | 429.9(76.9) | 5.6 | 0.001 |
| Period 2 | -439.2(89.0) | -4.9 | 0.002 |
| Period 3 | -328.7(288.8) | -1.2 | 0.56 |
| Period 4 | 233.2(276.6) | 0.8 | 0.46 |
| Period 5 | -98.9(137.2) | -0.7 | 0.52 |
| b) Observed number of species^a^ | |  |  |
| Intercept | 233.8(56.6) | 4.1 | 0.006 |
| Period 1 | 328.7(80.6) | 4.1 | 0.006 |
| Period 2 | -339.8(93.3) | -3.7 | 0.01 |
| Period 3 | -227.1(339.3) | -0.7 | 0.55 |
| Period 4 | 224.9(325.0) | 0.7 | 0.54 |
| Period 5 | -106.9(161.2) | -0.6 | 0.56 |
| c) Phylogenetic distance^a^ | |  |  |
| Intercept | 12.2(3.2) | 4.7 | 0.003 |
| Period 1 | 18.9(4.5) | 4.1 | 0.006 |
| Period 2 | -19.1(5.3) | -3.6 | 0.01 |
| Period 3 | -4.7(20.4) | -0.2 | 0.83 |
| Period 4 | 5.5(19.5) | 0.3 | 0.79 |
| Period 5 | -3.1(9.7) | -0.3 | 0.77 |

^a^Three different alpha diversity measures (Chao 1, observed number of species, and phylogenetic distance) were included in the analysis because they all rely on different approaches; *i.e*., they quantify bacterial alpha diversity based on different assumptions^55^.

Figure S1: Relative composition of main bacterial families on brown trout eggs at different spawning places and in corresponding water samples

Barplots for each sample are shown which include the relative abundance of the most abundant bacterial taxa (sequences comprise at least 1% in the whole dataset) on the y-axis and all 11 samples on the x-axis. Numbers correspond to spawning locations in Fig. 1 and Table 1 of the main manuscript and Table S1. ^a^At two locations water samples had been collected simultaneously with eggs: Gürbe (location 9) and Aare-Lerbestrasse (location 5).

Figure S2: Heatmaps of the most abundant 120 bacterial taxa on brown trout eggs at different spawning places and in corresponding water samples

Abundance of OTUs (Operational Taxonomic Units) is given in shades of blue (dark blue = low abundance, light blue = high abundance). Sample names of bacterial communities on brown trout eggs and corresponding water samples are given on the x-axis: numbers correspond to spawning locations in Fig. 1 and Table 1 of the main manuscript and Table S1. (At two locations water samples had been collected simultaneously with eggs: locations 9 and 5). Samples are ordered according to multidimensional scaling^56^. a) OTUs are shown at the family level and b) genus level, while all taxa that had been found in the negative control of only beads and buffers were filtered out in the remaining samples.

Figure S3: Rarefaction curves for filtered alpha diversity measures of bacterial communities on brown trout eggs and in corresponding water samples

Rarified mean alpha diversities for all nine sampling locations in this study (see Fig. 1 and Table 1 in the main manuscript for sample-IDs). Alpha diversities measured are (a) Chao 1, (b) observed number of species, and (c) phylogenetic distance.

Figure S4: Rarefaction curves for unfiltered alpha diversity measures of bacterial communities on brown trout eggs and in corresponding water samples

The negative control of only beads and buffers harbored bacterial sequences of 78 different bacterial taxa. These sequences were filtered out for downstream analysis in all other samples. In contrast to Fig. S3, this figures shows unfiltered rarified mean alpha diversities for all nine sampling locations in this study (see Fig. 1 and Table 1 in the main manuscript for sample-IDs). Alpha diversities measured are (a) Chao 1, (b) observed number of species, and (c) phylogenetic distance.

Figure S5: Number of bacterial taxa that could be found at different fractions of samples

In order to determine a core microbiome of brown trout eggs at their natural spawning places, OTUs (Operational Taxonomic Units) were ordered according to their presence at different fractions of samples. For downstream analysis OTUs had to be observed in seven out of nine samples (75 OTUs).

Figure S6: PICRUSt metagenome prediction predicted higher abundance of genes in 69 pathways of brown trout egg-associated bacterial communities relative to corresponding water samples

Metagenome prediction with PICRUSt v.1.0.0^57^ revealed gene functions that potentially differ significantly between the bacterial community of brown trout eggs at the late-eyed stage compared to two corresponding water samples, tested with a two-sample Welch’s test in STAMP v.2.1.2^58^ at a significance level of *p* < 0.05 (with Benjamini – Hochberg multiple testing correction controlling the FDR). Annotated KEGG pathways are shown at the third hierarchical level with detailed descriptions in Table S5, with genes in bacterial communities of blue = brown trout eggs, and yellow = two corresponding water samples. Mean difference with 95% confidence intervals is shown in middle and corrected *p*-values (*q*-values) on the right.

Figure S7: Spatial pattern of unfiltered bacterial community composition on brown trout eggs in the Aare system

Relationship of unfiltered pairwise, unweighted UniFrac bacterial community phylogenetic distances on brown trout eggs at natural spawning places in the Aare system and a) corrected geographic distances (km) or b) host genetic differentiation (*D_est_*). We did not find evidence for a significant relationship in both cases with *p* ≤ 0.05. In contrast to Fig. 3 in the main manuscript, this figure here was drawn without filtering out sequences that had been found in the negative control of only beads and buffers.

Figure S8: Two-dimensional PCoA plot of observed and simulated, unweighted UniFrac distances of brown trout egg-associated bacteria between the river Aare and a spawning place in the river Inn

In order to visualize the bootstrapping to create simulated trout egg-associated bacterial communities in the river Inn based on four bacterial communities in the river Aare, a Principal Coordinate Analysis plot was drawn. The four spawning locations that were used to sample from were Belp, Lerbestrasse, Wichtrach, and Zehndermätteli, (Innertkirchen was not included because it is relatively distant compared to the rest of the main river Aare spawning locations). Blue triangles = simulated bacterial community on brown trout eggs in the river Inn, green square = true bacterial community on brown trout eggs in the river Inn, red circle = mean of simulated bacterial communities on brown trout eggs in the river Inn.

Figure S9: Three-dimensional PCoA plot of unfiltered, unweighted UniFrac distances between bacterial communities on brown trout eggs and in water samples

A Principal Coordinate Analysis plot was drawn in order to visualize unfiltered phylogenetic distances between bacterial communities on brown trout eggs and two corresponding water samples. Numbers correspond to spawning locations in Fig. 1 of the main manuscript and Table S1, blue = water samples, red = brown trout egg samples, yellow = negative control of only beads and buffers. In contrast to Fig. 4 in the main manuscript, this figure here was drawn without filtering out sequences that had been found in the negative control of only beads and buffers.

Figure S10: Relationship of water temperature and unfiltered bacterial alpha diversities on brown trout eggs at natural spawning places

Circles = Chao 1, diamonds = observed number of species, and stars = phylogenetic distance (grey = river Inn, black = tributaries of the river Aare, and white = spawning locations in the main river Aare; see Fig. 1 and Table 1 in the main manuscript for location names). The three different unfiltered alpha diversity measures at each spawning location are connected by a dotted line while the mean incubation water temperature goes through the Chao 1 value. A solid horizontal line shows the upper and lower quartiles in mean water temperature. The range of Chao 1 and observed number of species is shown on the left y-axis and the range of phylogenetic distance is shown on the right y-axis. All spawning locations from both river systems are included. In contrast to Fig. 5 in the main manuscript, this figure here was drawn without filtering out sequences that had been found in the negative control of only beads and buffers.

Fig. S1


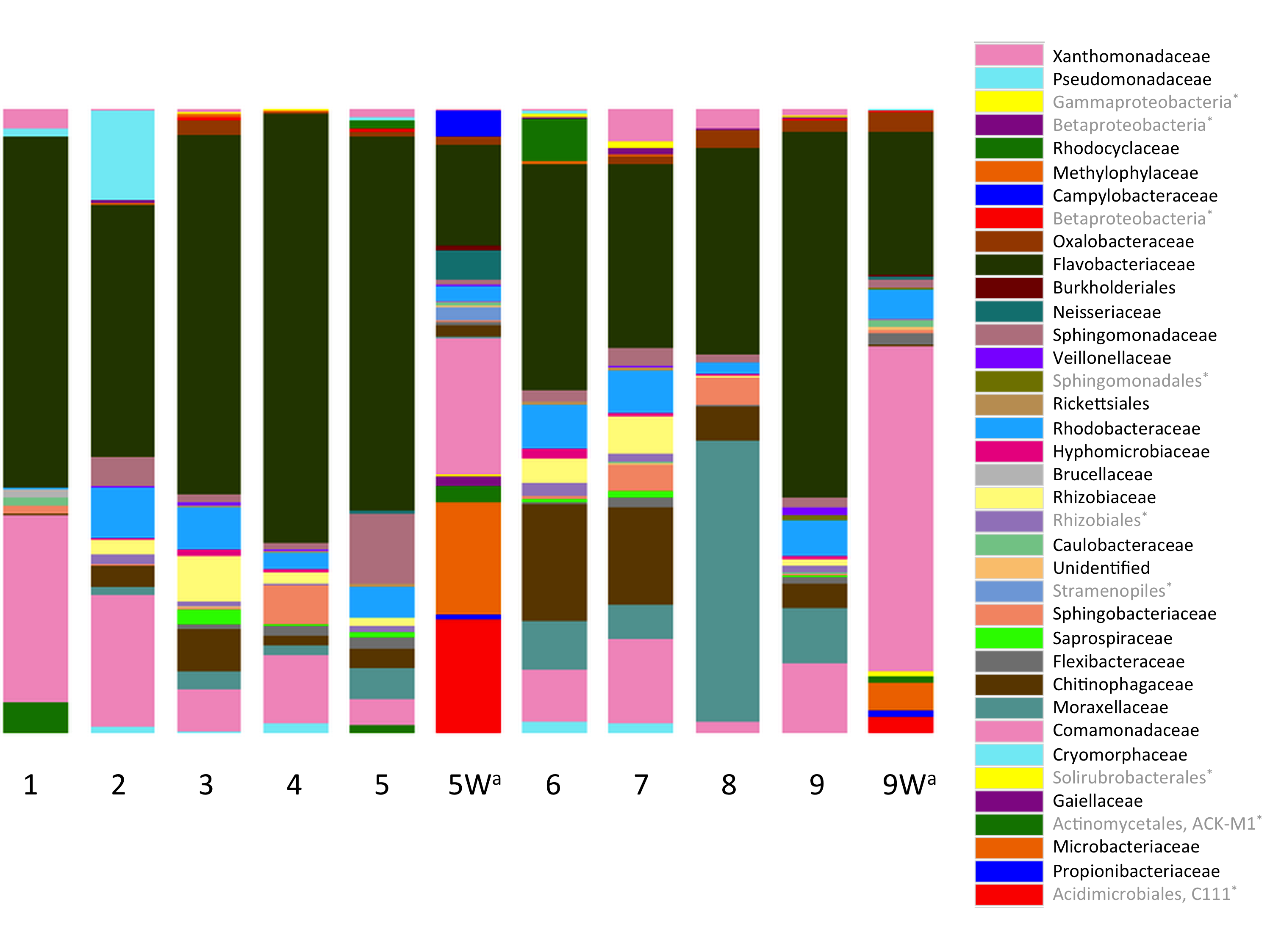


Fig. S2


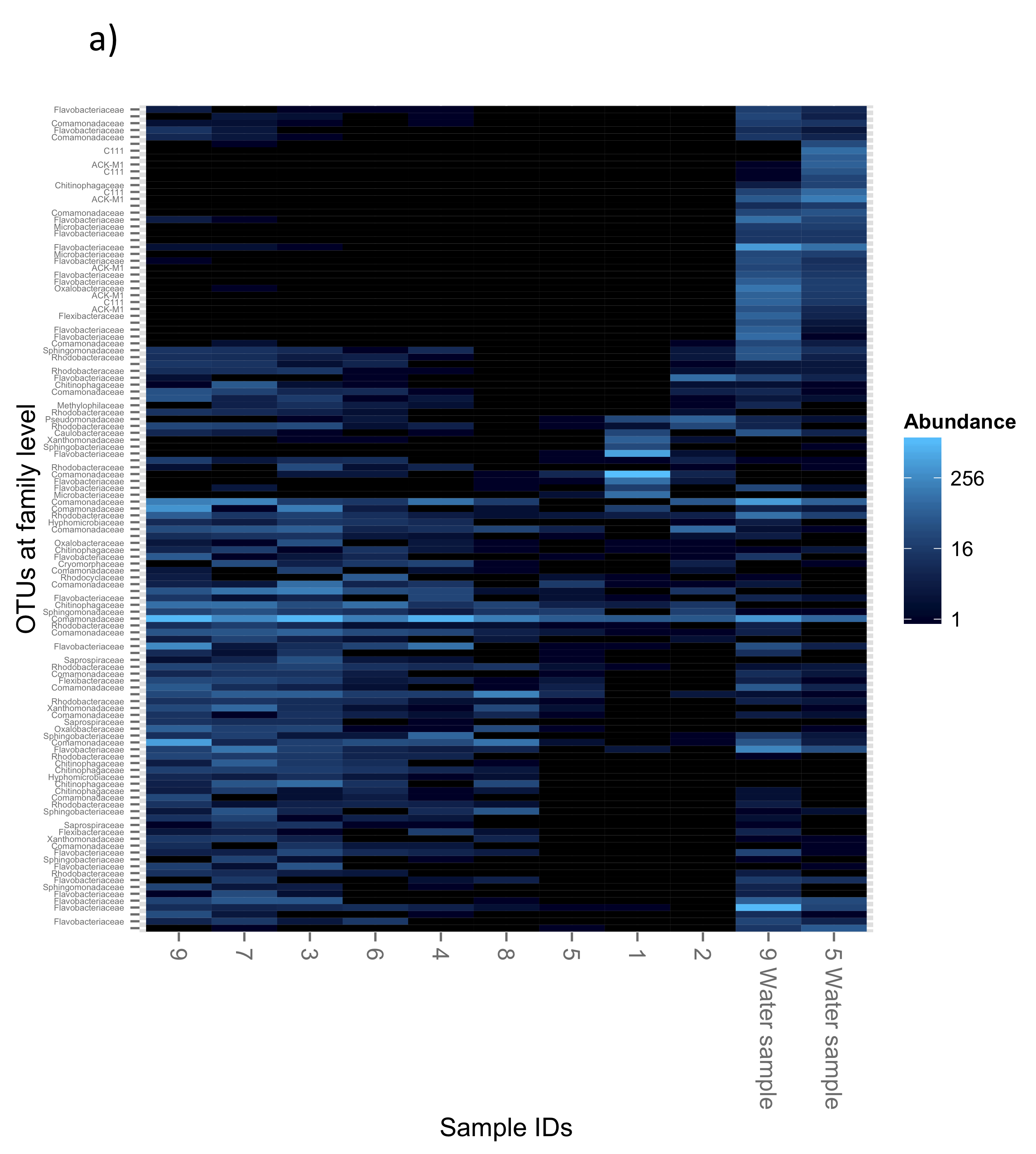


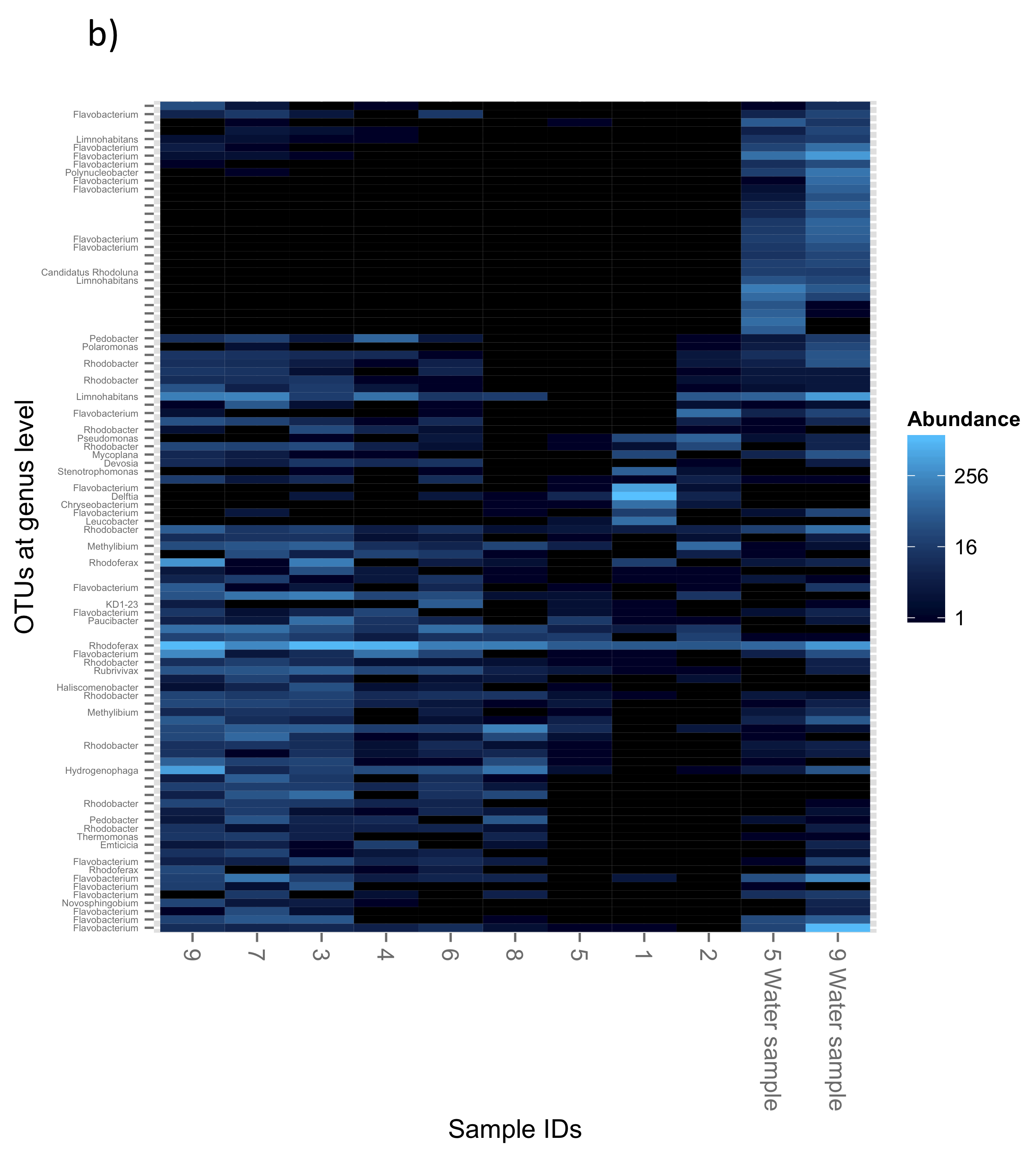


Fig. S3


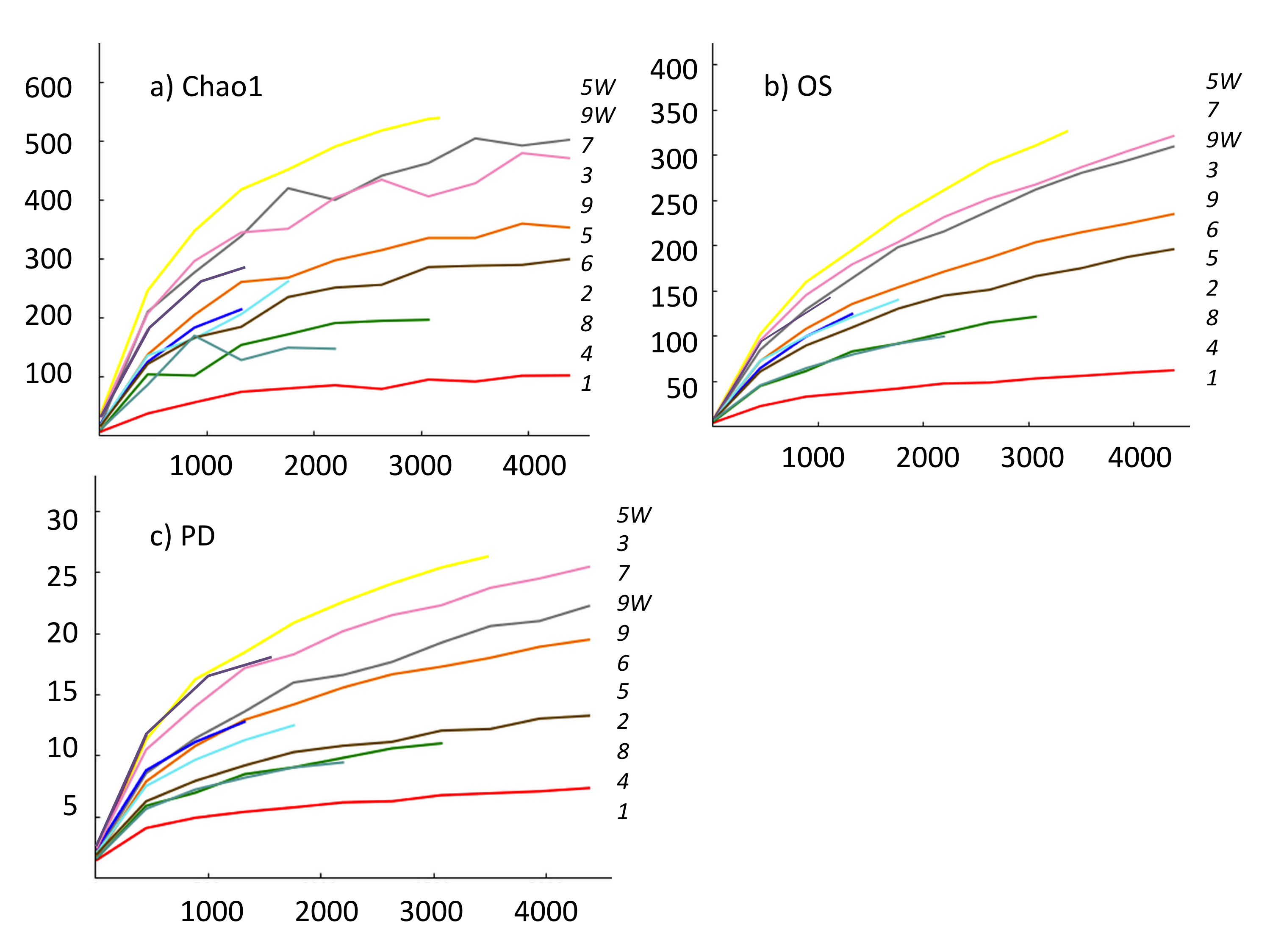


Fig. S4


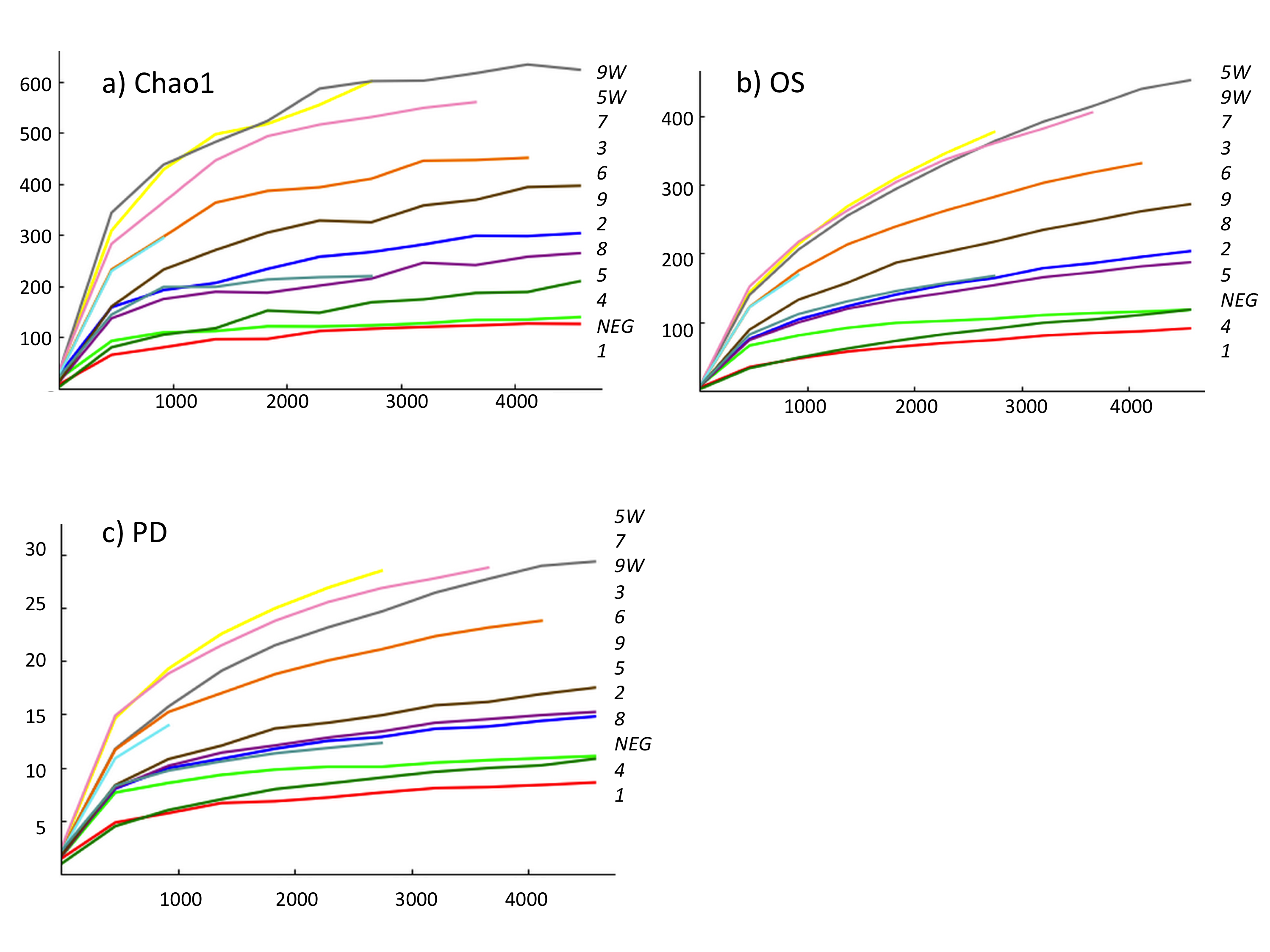


Fig. S5


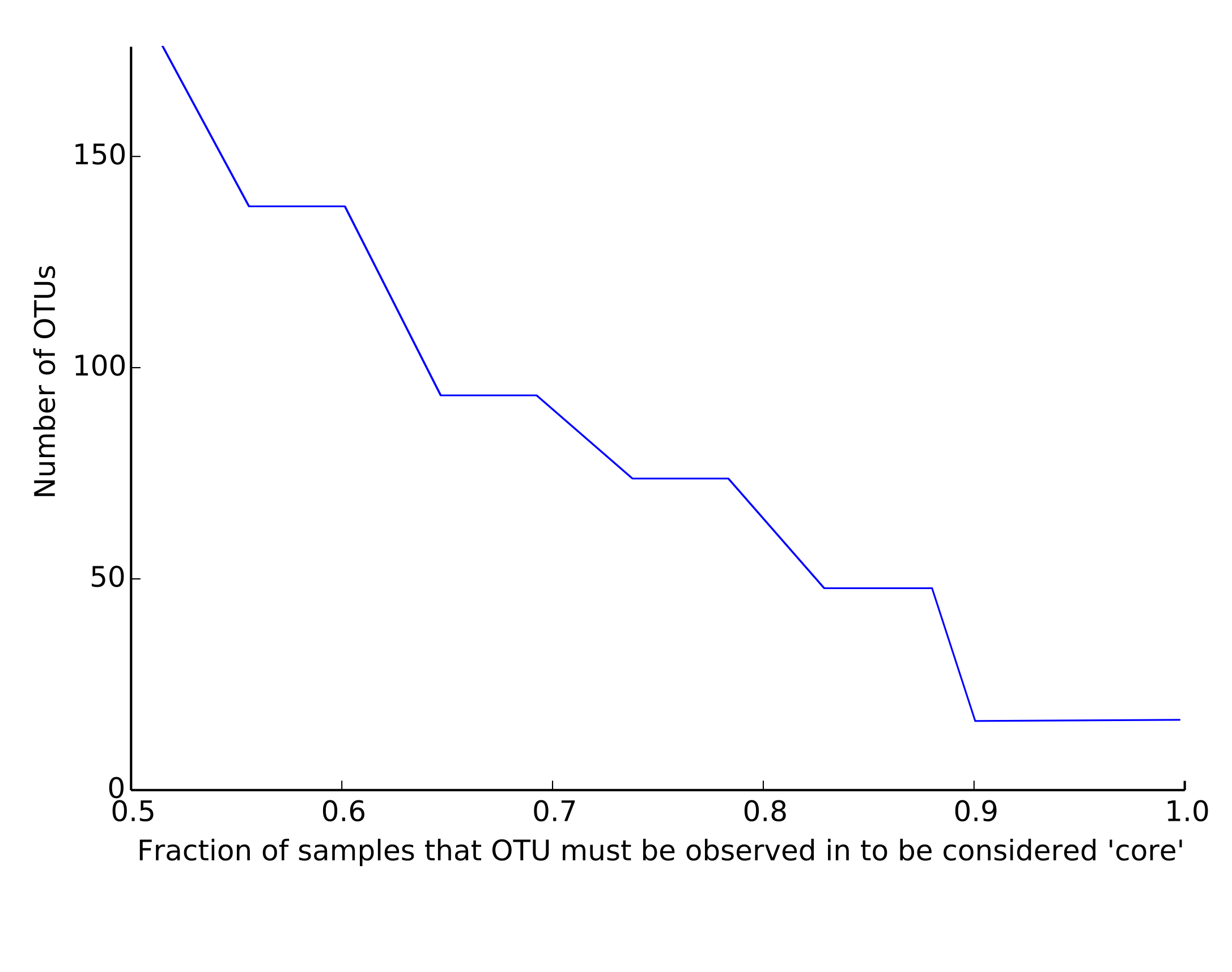


Fig. S6


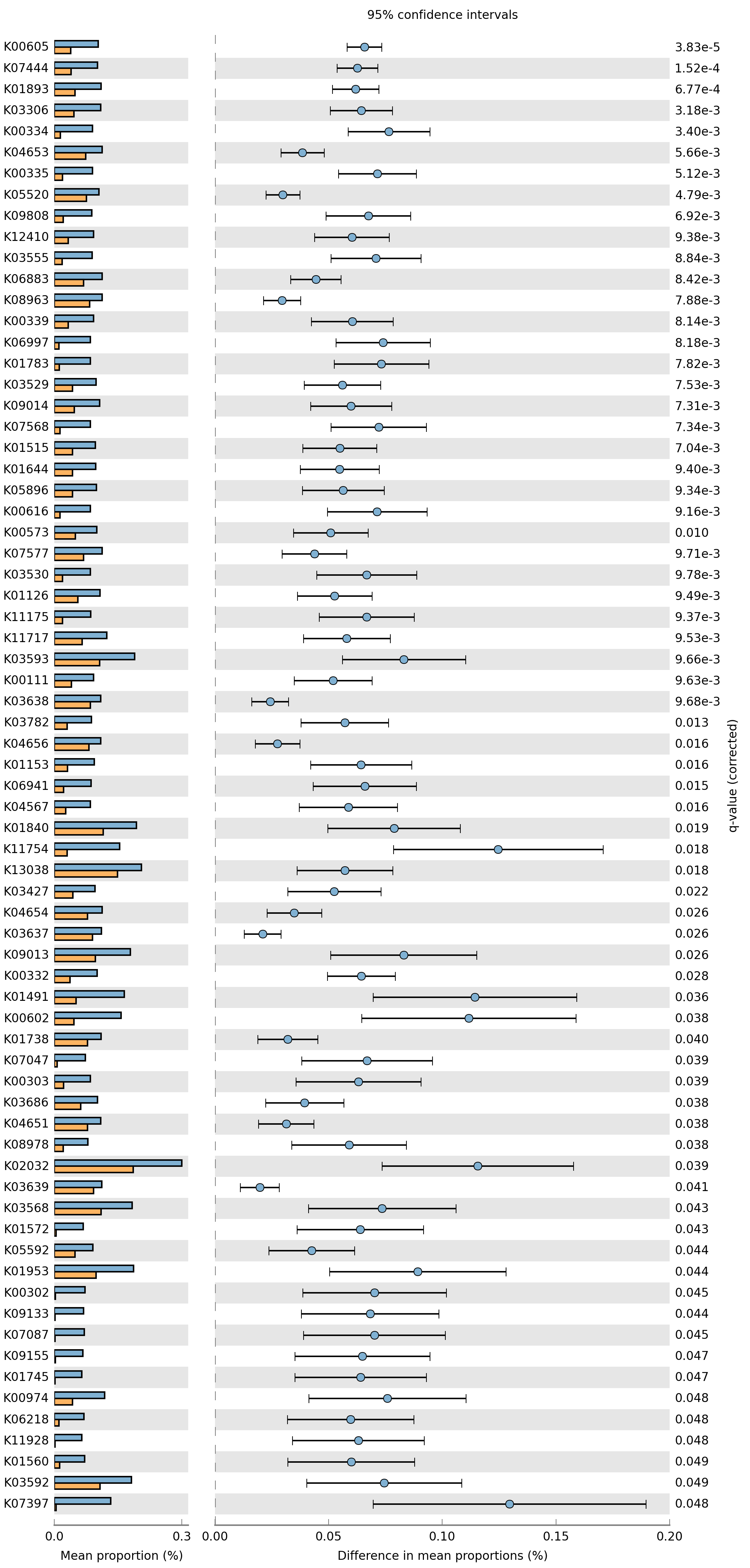


Fig. S7


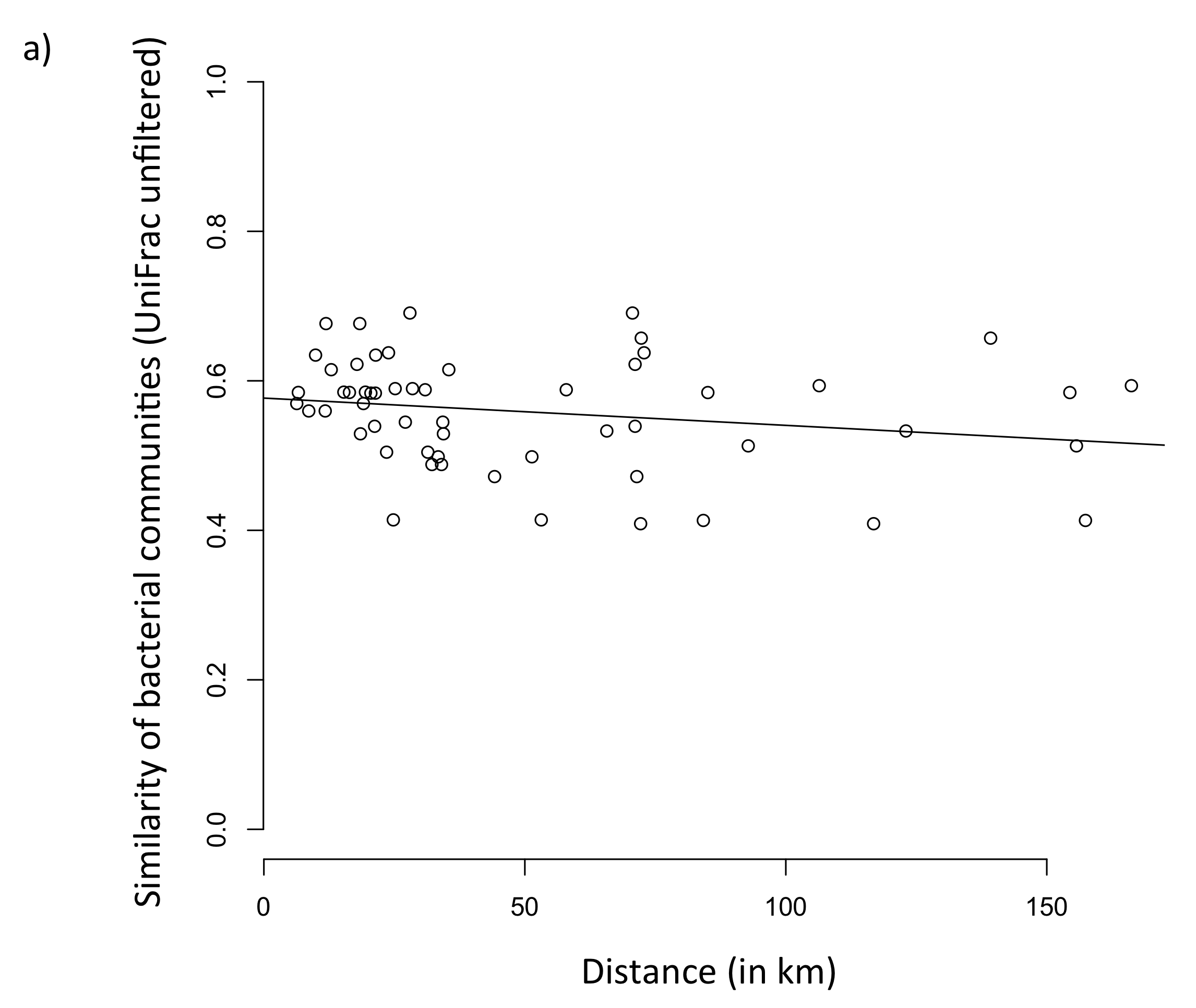


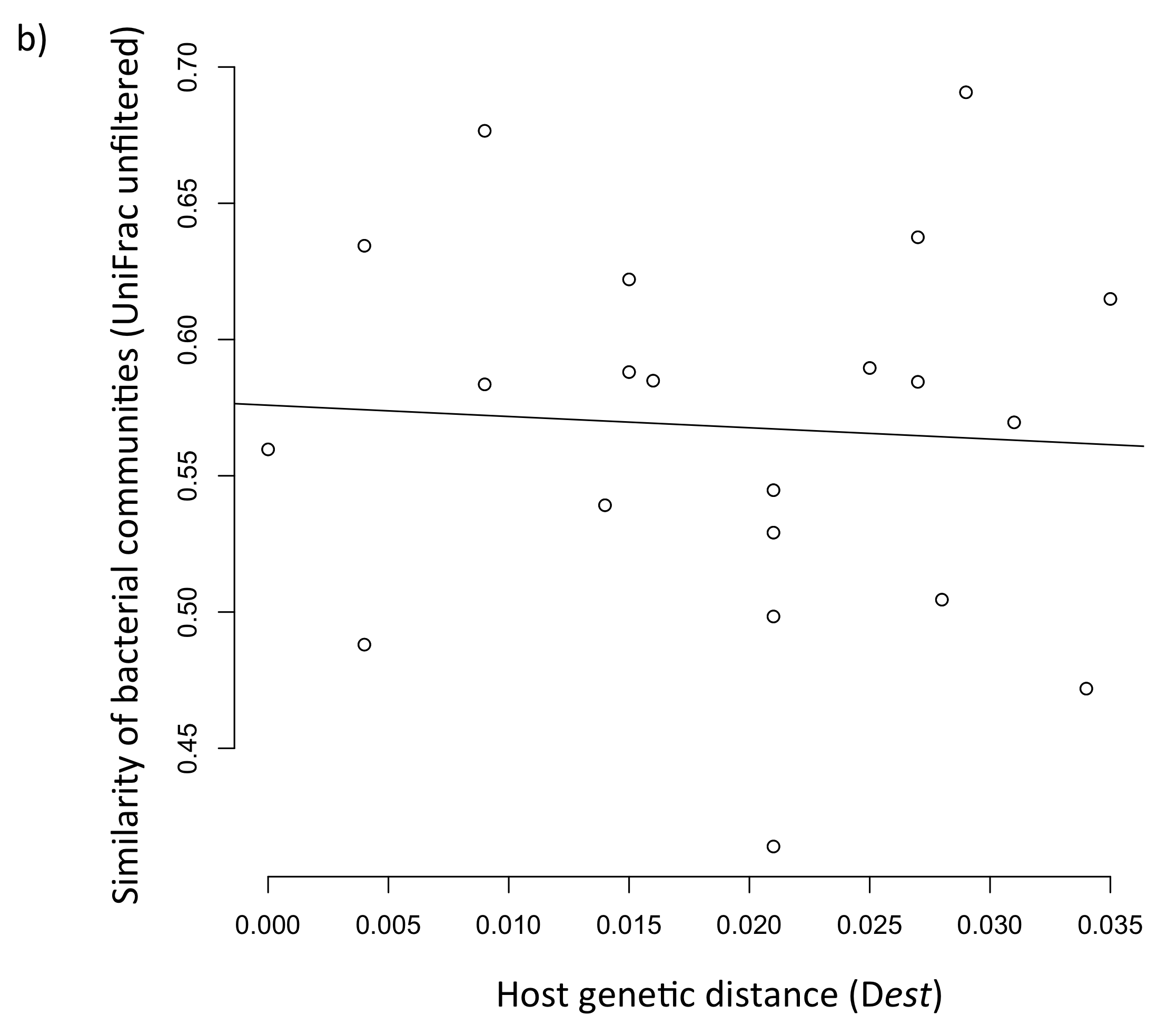


Fig. S8

**
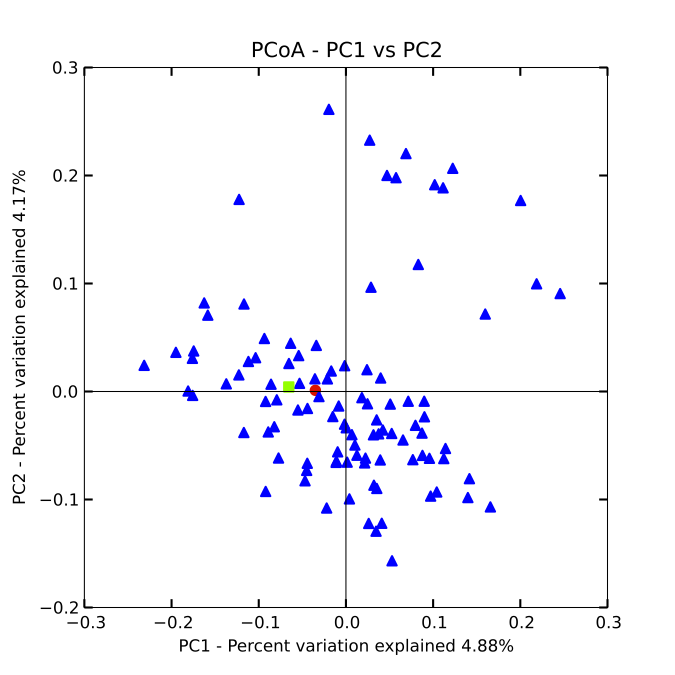
**

Fig. S9


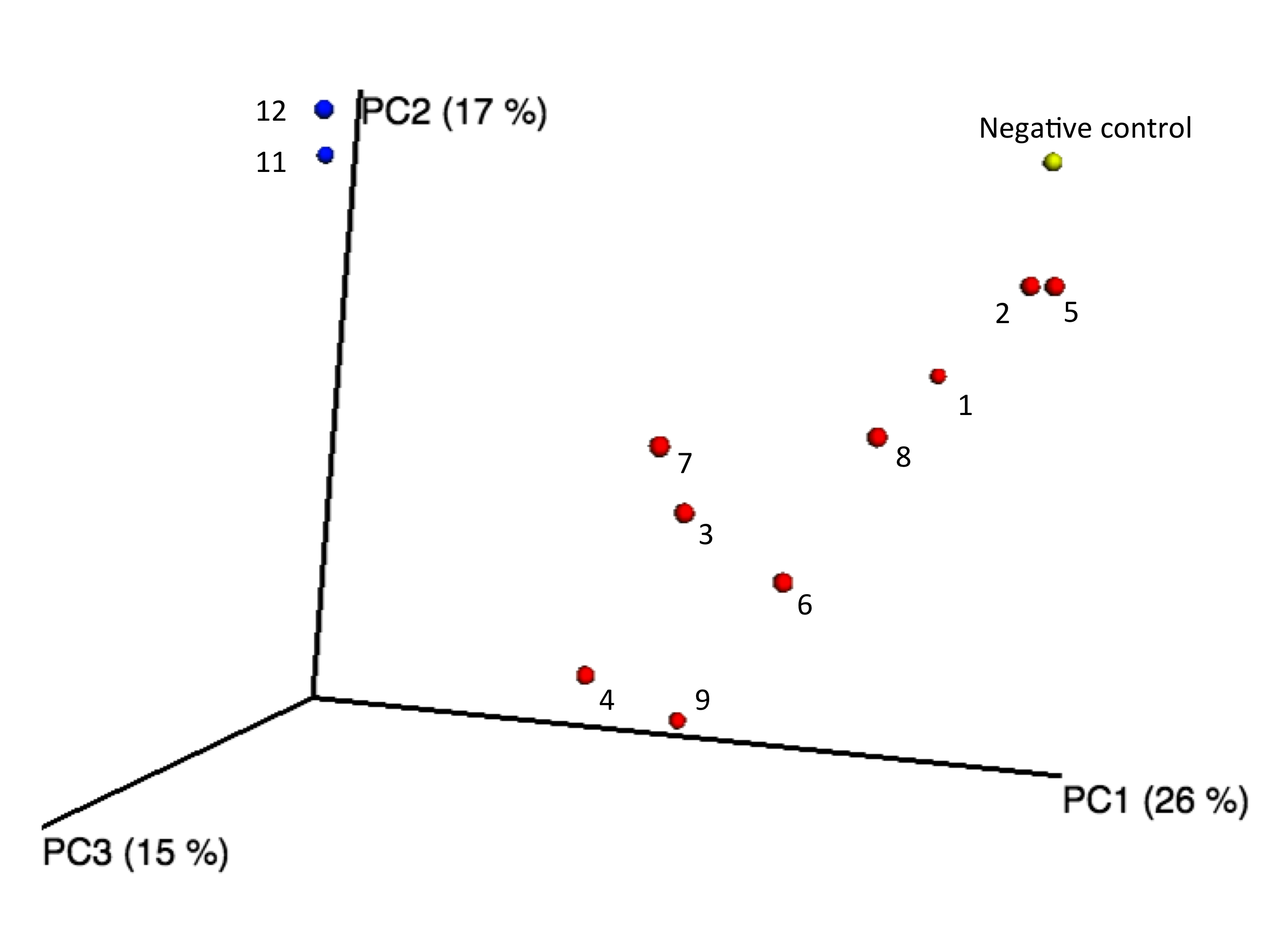


Fig. S10

**
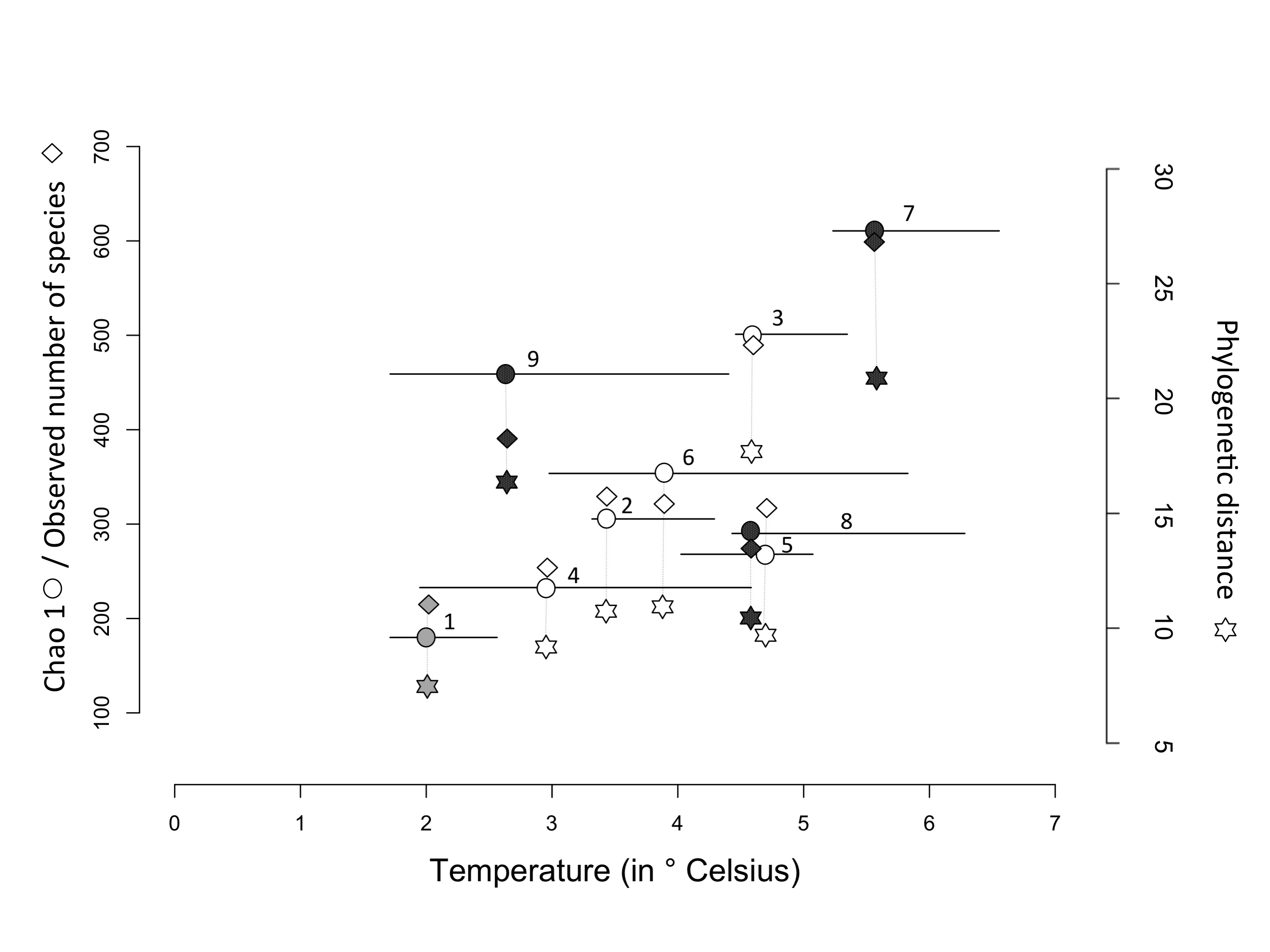
**

**Supplemental References**

1 Luczynski, M. & Kirklewska, A. Dependence of *Coregonus albula* embryogenesis rate on the incubation temperature. *Aquaculture* **42**, 43-55 (1984).

2 Hamady, M., Walker, J. J., Harris, J. K., Gold, N. J. & Knight, R. Error-correcting barcoded primers for pyrosequencing hundreds of samples in multiplex. *Nat. Methods* **5**, 235-237 (2008).

3 Wang, Y. & Qian, P. Y. Conservative fragments in bacterial 16S rRNA genes and primer design for 16S ribosomal DNA amplicons in metagenomic studies. *PLoS ONE* **4**, e7401 (2009).

4 Ley, R. E. *et al.* Evolution of mammals and their gut microbes. *Science* **320**, 1647-1651 (2008).

5 Berry, D., Ben Mahfoudh, K., Wagner, M. & Loy, A. Barcoded primers used in multiplex amplicon pyrosequencing bias amplification. *Appl. Environ. Microb.* **77**, 7846-7849 (2011).

6 Caporaso, J. G. *et al.* QIIME allows analysis of high-throughput community sequencing data. *Nat. Methods* **7**, 335-336 (2010).

7 Pantoja-Feliciano, I. G. *et al.* Biphasic assembly of the murine intestinal microbiota during early development. *ISME J.* **7**, 1112-1115 (2013).

8 McDonald, D. *et al.* An improved Greengenes taxonomy with explicit ranks for ecological and evolutionary analyses of bacteria and archaea. *ISME J.* **6**, 610-618 (2012).

9 Ong, S. H. *et al.* Species identification and profiling of complex microbial communities using shotgun illumina sequencing of 16S rRNA amplicon sequences. *PLoS ONE* **8**, e60811 (2013).

10 Edgar, R. C. Search and clustering orders of magnitude faster than BLAST. *Bioinformatics* **26**, 2460-2461 (2010).

11 McLaughlin, R. L. & Noakes, D. L. G. Going against the flow: an examination of the propulsive movements made by young brook trout in streams. *Can. J. Fish Aquat. Sci.* **55**, 853-860 (1998).

12 Neville, H. M., Dunham, J. B. & Peacock, M. M. Landscape attributes and life history variability shape genetic structure of trout populations in a stream network. *Landscape Ecol.* **21**, 901-916 (2006).

13 Thorpe, J. E. & Morgan, R. I. G. Periodicity in Atlantic salmon *Salmo salar* L smolt migration. *J. Fish Biol.* **12**, 541-548 (1978).

14 Salinger, D. H. & Anderson, J. J. Effects of water temperature and flow on adult salmon migration swim speed and delay. *T. Am. Fish. Soc.* **135**, 188-199 (2006).

15 Dionne, M., Caron, F., Dodson, J. J. & Bernatchez, L. Landscape genetics and hierarchical genetic structure in Atlantic salmon: the interaction of gene flow and local adaptation. *Mol. Ecol.* **17**, 2382-2396 (2008).

16 Meeuwig, M. H., Guy, C. S., Kalinowski, S. T. & Fredenberg, W. A. Landscape influences on genetic differentiation among bull trout populations in a stream-lake network. *Mol. Ecol.* **19**, 3620-3633 (2010).

17 Stelkens, R. B., Jaffuel, G., Escher, M. & Wedekind, C. Genetic and phenotypic population divergence on a microgeographic scale in brown trout. *Mol. Ecol.* **21**, 2896-2915 (2012).

18 Campbell, V., Legendre, P. & Lapointe, F. J. Assessing congruence among ultrametric distance matrices. *J. Classif.* **26**, 103-117 (2009).

19 Campbell, V., Legendre, P. & Lapointe, F. J. The performance of the congruence among distance matrices (CADM) test in phylogenetic analysis. *BMC Evol. Biol.* **11** (2011).

20 vegan: Community Ecology Package. R package version 2.0-8. (2013).

21 Wang, Q., Garrity, G. M., Tiedje, J. M. & Cole, J. R. Naive Bayesian classifier for rapid assignment of rRNA sequences into the new bacterial taxonomy. *Appl. Environ. Microb.* **73**, 5261-5267 (2007).

22 Madigan, M. T., Martinko, J. M., Stahl, D. & Clark, D. P. [*Brock Biology of Microorganisms*. 13th edn.] (Benjamin Cummings, San Francisco, CA, 2010).

23 Narciso-da-Rocha, C., Vaz-Moreira, I., Svensson-Stadler, L., Moore, E. R. B. & Manaia, C. M. Diversity and antibiotic resistance of *Acinetobacter* spp. in water from the source to the tap. *Appl. Microbiol. Biot.* **97**, 329-340 (2013).

24 Vandamme, P., Bernardet, J. F., Segers, P., Kersters, K. & Holmes, B. New perspectives in the classification of the Flavobacteria - Description of *Chryseobacterium* gen. nov., *Bergeyella* gen. nov., and *Empedobacter* nom. rev. *Int. J. Syst. Bacteriol.* **44**, 827-831 (1994).

25 *Microbe Wiki*, <https://microbewiki.kenyon.edu/index.php/Chryseobacterium_indologenes> (2015).

26 Hanski, I. *et al.* Environmental biodiversity, human microbiota, and allergy are interrelated. *P. Natl. Acad. Sci. USA* **109**, 8334-8339 (2012).

27 Mashima, I. & Nakazawa, F. The influence of oral *Veillonella* species on biofilms formed by *Streptococcus* species. *Anaerobe* **28**, 54-61 (2014).

28 Otto, M. *Staphylococcus epidermidis* - the 'accidental' pathogen. *Nat. Rev. Microbiol.* **7**, 555-567 (2009).

29 Schleifer, K. H. & Kloos, W. E. Isolation and characterization of Staphylococci from human skin .1. Amended descriptions of *Staphylococcus epidermidis* and *Staphylococcus saprophyticus* and descriptions of three new species - *Staphylococcus cohnii*, *Staphylococcus haemolyticus*, and *Staphylococcus xylosus*. *Int. J. Syst. Bacteriol.* **25**, 50-61 (1975).

30 Kubota, M., Hagiwara, N. & Shirakawa, T. Disinfection of seeds of cucurbit crops infested with *Acidovorax citrulli* with dry heat treatment. *J. Phytopathol.* **160**, 364-368 (2012).

31 Kawamura, Y. *et al.* Genus *Enhydrobacter* Staley *et al.* 1987 should be recognized as a member of the family Rhodospirillaceae within the class Alphaproteobacteria. *Microbiol. Immunol.* **56**, 21-26 (2012).

32 Staley, J. T., Irgens, R. L. & Brenner, D. J. *Enhydrobacter aerosaccus* gen. nov., sp. nov., a gas-vacuolated, facultatively anaerobic, heterotrophic rod. *Int. J. Syst. Bacteriol.* **37**, 289-291 (1987).

33 Murray, B. E. The Life and times of the *Enterococcus*. *Clin. Microbiol. Rev.* **3**, 46-65 (1990).

34 Patureau, D. *et al.* *Microvirgula aerodenitrificans* gen. nov., sp. nov., a new gram-negative bacterium exhibiting co-respiration of oxygen and nitrogen oxides up to oxygen-saturated conditions. *Int. J. Syst. Bacteriol.* **48**, 775-782 (1998).

35 Perry, A. & Lambert, P. *Propionibacterium acnes*: infection beyond the skin. *Expert. Rev. Anti-Infe.* **9**, 1149-1156 (2011).

36 Austin, B. & Austin, D. A. [*Bacterial Fish Pathogens, Disease of Farmed and Wild Fish*. Fifth edn.] (Springer, London, UK, 2007).

37 Willems, A. & de Vos, P. [Comamonas in *Prokaryotes: A Handbook on the Biology of Bacteria.* Vol 5, Third edn.] 723-736 (Springer, London, UK, 2006).

38 Sato, T., Matsuyama, J., Sato, M. & Hoshino, E. Differentiation of *Veillonella atypica*, *Veillonella dispar* and *Veillonella parvula* using restricted fragment-length polymorphism analysis of 16S rDNA amplified by polymerase chain reaction. *Oral. Microbiol. Immun.* **12**, 350-353 (1997).

39 Kirby, J. T., Sader, H. S., Walsh, T. R. & Jones, R. N. Antimicrobial susceptibility and epidemiology of a worldwide collection of *Chryseobacterium* spp.: Report from the SENTRY Antimicrobial Surveillance Program (1997-2001). *J. Clin. Microbiol.* **42**, 445-448 (2004).

40 Grimes, D. J., Woese, C. R., MacDonell, M. T. & Colwell, R. R. Systematic study of the genus *Vogesella* gen. nov. and its type species, *Vogesella indigofera* comb. nov. *Int. J. Syst. Bacteriol.* **47**, 19-27 (1997).

41 El Kafsi, H. *et al.* *Lactobacillus delbrueckii* ssp. *lactis* and ssp. *bulgaricus*: a chronicle of evolution in action. *BMC Genomics* **15** (2014).

42 Gan, H. M., Hudson, A. O., Rahman, A. Y. A., Chan, K. G. & Savka, M. A. Comparative genomic analysis of six bacteria belonging to the genus *Novosphingobium*: insights into marine adaptation, cell-cell signaling and bioremediation. *BMC Genomics* **14** (2013).

43 Petrova, M. I., Lievens, E., Malik, S., Imholz, N. & Lebeer, S. *Lactobacillus* species as biomarkers and agents that can promote various aspects of vaginal health. *Front. Physiol.* **6** (2015).

44 Keith, E. R., Podmore, R. G., Anderson, T. P. & Murdoch, D. R. Characteristics of *Streptococcus pseudopneumoniae* isolated from purulent sputum samples. *J. Clin. Microbiol.* **44**, 923-927 (2006).

45 Partensky, F., Hess, W. R. & Vaulot, D. *Prochlorococcus*, a marine photosynthetic prokaryote of global significance. *Microbiol. Mol. Biol. R.* **63**, 106-127 (1999).

46 Takeuchi, M., Hamana, K. & Hiraishi, A. Proposal of the genus *Sphingomonas sensu stricto* and three new genera, *Sphingobium*, *Novosphingobium* and *Sphingopyxis*, on the basis of phylogenetic and chemotaxonomic analyses. *Int. J. Syst. Evol. Micr.* **51**, 1405-1417 (2001).

47 Verma, H. *et al.* Comparative genomic analysis of nine *Sphingobium* strains: insights into their evolution and hexachlorocyclohexane (HCH) degradation pathways. *BMC Genomics* **15** (2014).

48 Newton, R. J., Jones, S. E., Eiler, A., McMahon, K. D. & Bertilsson, S. A guide to the natural history of freshwater lake bacteria. *Microbiol. Mol. Biol. R.* **75**, 14-49 (2011).

49 Gillis, M. & de Ley, J. [The Genera Chromobacterium and Janthinobacterium in *Prokaryotes: A Handbook on the Biology of Bacteria.* Vol 5, Third edn.] 737-746 (Springer, London, UK, 2006).

50 Pantanella, F. *et al.* Violacein and biofilm production in *Janthinobacterium lividum*. *J. Appl. Microbiol.* **102**, 992-999 (2007).

51 Leisner, J. J., Laursen, B. G., Prevost, H., Drider, D. & Dalgaard, P. *Carnobacterium*: positive and negative effects in the environment and in foods. *FEMS Microbiol. Rev.* **31**, 592-613 (2007).

52 Looney, W. J., Narita, M. & Muhlemann, K. *Stenotrophomonas maltophilia*: an emerging opportunist human pathogen. *Lancet. Infect. Dis.* **9**, 312-323 (2009).

53 von Siebenthal, B. A., Jacob, A. & Wedekind, C. Tolerance of whitefish embryos to *Pseudomonas fluorescens* linked to genetic and maternal effects, and reduced by previous exposure. *Fish Shellfish Immun.* **26**, 531–535 (2009).

54 Eldar, A. & Ghittino, C. *Lactococcus garvieae* and *Streptococcus iniae* infections in rainbow trout *Oncorhynchus mykiss*: similar, but different diseases. *Dis. Aquat. Organ.* **36**, 227-231 (1999).

55 Gotelli, N. J. & Chao, A. [*Encyclopedia of Biodiversity.* Vol. 5] [Levin, S.A. (ed.)]) 195-211 (Academic Press, Waltham, MA, 2013).

56 Rajaram, S. & Oono, Y. NeatMap - non-clustering heat map alternatives in R. *BMC Bioinformatics* **11** (2010).

57 Langille, M. G. I. *et al.* Predictive functional profiling of microbial communities using 16S rRNA marker gene sequences. *Nat. Biotechnol.* **31**, 814-823 (2013).

58 Parks, D. H., Tyson, G. W., Hugenholtz, P. & Beiko, R. G. STAMP: statistical analysis of taxonomic and functional profiles. *Bioinformatics* **30**, 3123-3124 (2014).
